# A cooperative network at the nuclear envelope counteracts LINC-mediated forces during oogenesis in *C. elegans*

**DOI:** 10.1126/sciadv.abn5709

**Published:** 2023-07-12

**Authors:** Chenshu Liu, Rachel Rex, Zoe Lung, John S. Wang, Fan Wu, Hyung Jun Kim, Liangyu Zhang, Lydia L. Sohn, Abby F. Dernburg

**Affiliations:** ^1^ California Institute for Quantitative Biosciences (QB3) and Department of Molecular and Cell Biology, University of California Berkeley, Berkeley, CA 94720, USA.; ^2^ Howard Hughes Medical Institute, Chevy Chase, MD 20815, USA.; ^3^ Department of Mechanical Engineering, University of California Berkeley, Berkeley, CA 94720, USA.; ^4^ Department of Biological Sciences and Engineering, Life Sciences Division, Lawrence Berkeley National Laboratory, Berkeley, CA 94720, USA.

## Abstract

Oogenesis involves transduction of mechanical forces from the cytoskeleton to the nuclear envelope (NE). In *Caenorhabditis elegans*, oocyte nuclei lacking the single lamin protein LMN-1 are vulnerable to collapse under forces mediated through LINC (linker of nucleoskeleton and cytoskeleton) complexes. Here, we use cytological analysis and in vivo imaging to investigate the balance of forces that drive this collapse and protect oocyte nuclei. We also use a mechano-node-pore sensing device to directly measure the effect of genetic mutations on oocyte nuclear stiffness. We find that nuclear collapse is not a consequence of apoptosis. It is promoted by dynein, which induces polarization of a LINC complex composed of Sad1 and UNC-84 homology 1 (SUN-1) and ZYGote defective 12 (ZYG-12). Lamins contribute to oocyte nuclear stiffness and cooperate with other inner nuclear membrane proteins to distribute LINC complexes and protect nuclei from collapse. We speculate that a similar network may protect oocyte integrity during extended oocyte arrest in mammals.

## Teaser

Nuclear lamins and other inner nuclear membrane proteins work together to protect oocyte nuclei from collapse.

## INTRODUCTION

Oogenesis produces haploid ova from diploid progenitors through meiosis and oocyte differentiation and maturation. During meiosis, homologous chromosomes must pair, synapse, and undergo controlled DNA double-strand breaks (DSBs) and repair (*1*, *2*). Oocyte nuclei are subjected to mechanical forces generated by the cytoskeleton and transmitted to the nuclear envelope (NE) through linker of nucleoskeleton and cytoskeleton (LINC) complexes (*3*–*11*). These forces are important for meiotic chromosome dynamics, nuclear positioning, and migration of maturing oocytes in the germ line (*12*, *13*). In mammals, mechanical forces are important to maintain ovarian reserve during the long period of dormancy (*14*, *15*), yet such forces can also jeopardize fertility if the stiffness of the NE is compromised by mutation (*16*). The mechanisms that protect oocyte nuclei from mechanical stress are not well studied.

The nuclear lamina is a meshwork composed of lamins, type V intermediate filament proteins (*17*–*19*). Lamins self-assemble into higher-order structures, which confer mechanical stability to the NE and protect nuclear contents (*20*, *21*). Lamin mutations cause diverse diseases, collectively called laminopathies (*22*, *23*). Defects in the nuclear lamina can cause nuclear rupture, DNA damage, alterations in chromosomal architecture, or loss of genome integrity in somatic tissues (*24*–*27*).

A lamin meshwork is present in metazoan oocytes and likely reinforces the NE during oogenesis (*28*, *29*). Consistent with this idea, women carrying *LMNA* mutations are at higher risks for infertility and obstetric complications (*30*). In mammals, investigation of the roles of the nuclear lamina during oogenesis is confounded by the presence of multiple lamin isoforms in the germ line (*29*). *Caenorhabditis elegans* has only one lamin protein (LMN-1). Null mutations in *lmn-1* result in near-complete sterility and embryonic lethality, with hypercondensed chromatin observed in oocyte nuclei (*31*–*34*). However, pleiotropic effects on both somatic and germline tissues in the *lmn-1*–null mutant, such as atrophic gonads and accelerated aging, have obscured the direct roles of LMN-1 during oogenesis. Here, we investigate the role of LMN-1 and other NE components in protecting nuclear integrity during oogenesis in *C. elegans*.

## RESULTS

### Induced degradation of LMN-1 in the *C. elegans* germ line causes apoptosis-independent nuclear collapse in maturing oocytes

**Fig. 1.**
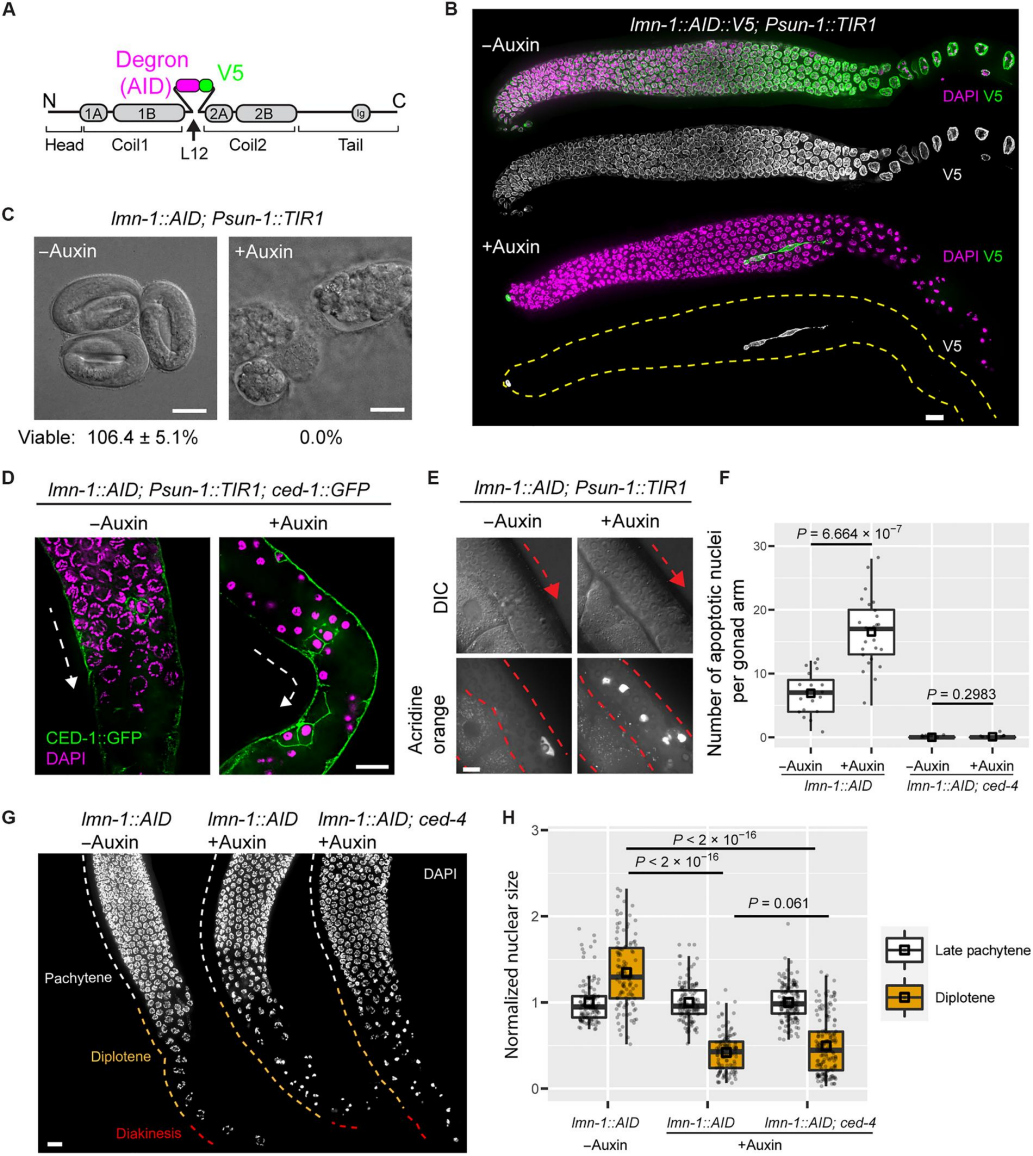
Induced degradation of LMN-1 in the *C. elegans* germ line causes apoptosis-independent nuclear collapse in maturing oocytes. (**A**) Domain organization of *C. elegans* LMN-1, indicating where we inserted a degron (AID) and V5 epitope. (**B**) LMN-1::V5::AID immunofluorescence in gonads dissected from adult hermaphrodites. Meiosis progresses from left to right. 4′,6-Diamidino-2-phenylindole (DAPI), magenta; anti-V5, green. Grayscale images show LMN-1::V5::AID alone. Following auxin treatment, LMN-1::V5::AID is retained in the somatic distal tip and sheath cells, which do not express TIR1. Images are maximum-intensity projections and were scaled identically. Scale bar, 10 μm. (**C**) Differential interference contrast (DIC) images of embryos laid by hermaphrodites following auxin treatment for 48 hours and (−)auxin controls. Scale bars, 20 μm. Some embryos are typically overlooked when counting, resulting in viability counts exceeding 100%. (**D**) Immunofluorescence of CED-1::GFP. Following depletion of LMN-1 depletion, nuclei in the proximal gonad have hypercondensed chromatin, and some are surrounded by CED-1::GFP, which marks engulfing cells. Dashed lines indicate the direction of meiotic progression. Scale bar, 10 μm. (**E**) Germ lines in living animals showing gonad morphology (DIC) and apoptotic nuclei stained with acridine orange. Red dashed lines and arrows indicate the contour of gonad and the direction of meiotic progression. Scale bar, 10 μm. (**F**) Quantification of apoptotic nuclei based on acridine orange staining. All worms were homozygous for *P_sun-1_::TIR1*. Each dot represents one animal. Medians (black crossbars) and means (black boxes) are shown. (**G**) Nuclear morphology during late meiotic prophase. All worms were homozygous for *P_sun-1_::TIR1*. Meiotic prophase progresses from top to bottom; stages are annotated on the basis of their anatomical positions within control gonads. Scale bar, 10 μm. (**H**) Quantification of nuclear size. Medians (black crossbars) and means (black boxes) are shown. Colors correspond to the regions shown in (G). All statistics are in Materials and Methods and data S1.

To focus on the functions of LMN-1 during gametogenesis, we created a conditional allele by inserting a degron to enable auxin-inducible degradation (*35*). Lamin protein-protein interactions are easily perturbed by the addition of epitope tags or other sequences (*36*). However, we identified a poorly conserved and highly flexible linker region (*37*) that tolerated insertion of the degron and V5 epitope without disrupting LMN-1 function. In a strain also expressing the F-box protein Transport inhibitor response 1 (TIR1) from *Arabidopsis thaliana* under germline-specific regulation (*35*), we found that animals homozygous for this degron-tagged *lmn-1* allele were fully viable and fertile in the absence of auxin (Fig. 1A, fig. S1A, and table S1). When exposed to indole acetic acid (IAA; auxin), we observed robust depletion of LMN-1 in the germ line; the immunofluorescence signal remaining in the somatic distal tip cell and germline sheath cells also provided a convenient control. All embryos laid on auxin-containing plates failed to develop (Fig. 1, B and C, and fig. S1B), consistent with an important role for maternally contributed LMN-1 in embryogenesis (*31*).

Depletion of LMN-1 recapitulated germline defects previously described in *lmn-1*–null mutants: marked compaction of nuclei in the proximal region of the gonad, near the uterus, the region corresponding to diplotene and diakinesis in wild-type hermaphrodites (*31*–*33*). This nuclear collapse is characterized by a marked reduction in nuclear volume, which normally increases gradually from late pachytene to diplotene (Fig. 1, B, G, and H, and figs. S2 and S3). We also observed persistent RADiation sensitivity abnormal 51 (RAD-51) foci, a marker for DSB repair intermediates (*38*). While most of these RAD-51 foci depended on the meiosis-specific endonuclease homolog of yeast SPOrulation gene 11 (SPO-11), some RAD-51 and GFP::COSA-1 (CrossOver Site Associated 1) foci, which mark designated crossover sites (*39*), were also detected in the absence of SPO-11 (fig. S4). This is consistent with previous evidence that disruption of the nuclear lamina can cause DNA damage (*24*, *25*).

Unrepaired DNA damage promotes apoptosis of *C. elegans* oocytes (*40*, *41*), resulting in hypercondensed chromatin morphology (*42*). We investigated whether the condensed nuclei observed following LMN-1 depletion were apoptotic using two different markers: We stained live worms with acridine orange (*40*) and also introduced a fluorescent CEll Death abnormality 1 (CED-1)::GFP marker, which localizes to the membranes of apoptotic cells undergoing engulfment (*43*). Both markers indicated a pronounced increase in apoptosis in hermaphrodite germ lines depleted of LMN-1 (Fig. 1, D to F). However, we observed similar effects on nuclear morphology and DNA condensation even in the absence of CED-4, which is essential for germline apoptosis (Fig. 1, F to H) (*43*). Together with prior evidence that various other mutations lead to elevated germline apoptosis without causing hypercondensed nuclei in the proximal gonad (*41*, *43*–*46*), this implies that nuclear collapse is not a consequence of apoptosis. We also tested whether nuclear collapse might be a consequence of autophagy but found that co-depleting the essential activator autophagy related 7 ortholog (ATG-7) (*47*, *48*) in the germ line did not rescue nuclear collapse because of LMN-1 depletion (fig. S5).

### Nuclear collapse is driven by force-induced destabilization of the NE

**Fig. 2.**
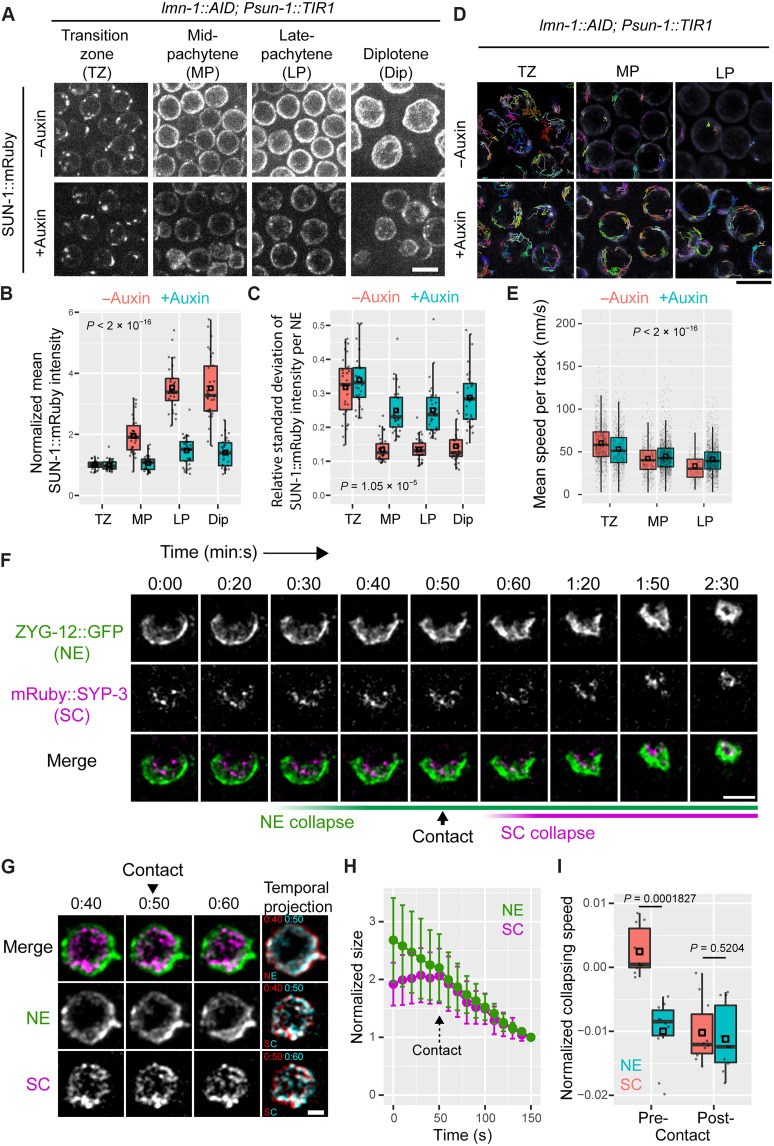
Dynamics of nuclear collapse. (**A** to **E**) Prolonged LINC complex clustering and mobility following LMN-1 depletion. (A) Maximum-intensity projection images of SUN-1::mRuby fluorescence at different stages of meiotic prophase. Images are scaled identically. Scale bar, 5 μm. (B) Mean SUN-1::mRuby intensity per nucleus, normalized against transition zone nuclei. The periphery of each nucleus was manually segmented and quantified from additive projection after background subtraction. TZ, transition zone; MP, mid-pachytene; LP, late pachytene; Dip, diplotene. (C) SUN-1::mRuby clustering, defined as the ratio of the standard deviation (SD) to the mean fluorescence intensity at the periphery of each nucleus, measured as in (B) (also see fig. S8A). (D) Grayscale images of SUN-1::mRuby overlaid with color-coded trajectories of SUN-1::mRuby patches. Scale bar, 5 μm. (E) Mean speed of individual SUN-1::mRuby patches at the NE. (**F**) Representative time-lapse images showing a collapsing diplotene nucleus after LMN-1 depletion. Maximum-intensity projections were scaled identically. The frame showing initial contact between NE and SC was shown. Note the asymmetric distribution of ZYG-12::GFP at the NE before and during collapse. Time stamps, min:s. Scale bar, 5 μm. (**G**) Another example of a collapsing late pachytene/early diplotene nucleus, displayed as temporal projections of three successive time points to highlight the shrinking of NE without concomitant contraction of SC from 40 to 50 s. Time stamps are min:s. Scale bar, 2 μm. (**H**) Quantification of NE and SC sizes measured from time-lapse images. The point of contact between NE and SC was used to align data, and the final frame was used to for normalization. Means ± SD are plotted. (**I**) Quantification of the collapse rate before (“pre-”) and after (“post-”) contact between NE and SC. Medians (black crossbars) and means (black boxes) are shown (B, C, E, and I). All statistics are in Materials and Methods and data S1.

To better understand the process leading to nuclear collapse, we introduced fluorescent NE markers SUN-1::mRuby or ZYG-12::GFP to enable live-cell imaging following LMN-1 depletion (movie S1). We also combined ZYG-12::GFP with a fluorescently labeled synaptonemal complex (SC) protein, mRuby::SYP-3, to monitor the nuclear distribution of chromosomes (Fig. 2) (*49*). The LINC complex components SUN-1 and its Klarsicht, ANC-1, Syne homology (KASH) partner ZYG-12 span the inner nuclear membrane (INM) and outer nuclear membrane (ONM), respectively, and play important roles in connecting chromosomes to the microtubule cytoskeleton during early meiosis in the distal region of the germline (*3*, *4*). During early prophase, SUN-1 and ZYG-12 connect specialized chromosome regions known as meiotic pairing centers to cytoplasmic dynein to promote movement of chromosomes along the NE. This movement is important for homolog pairing and synapsis and normally abates once chromosomes are fully synapsed (*4*). In germ lines depleted of LMN-1, although there was no delay in pairing or synapsis (fig. S6), clustered LINC complex persisted from early meiosis through the proximal region (Fig. 2, A to C, and figs. S7 and S8A). Particle tracking in live animals revealed that these patches remained highly mobile within the NE throughout meiotic prophase (Fig. 2, D and E, and movies S2 and S3). LMN-1 is normally phosphorylated during early meiosis and then dephosphorylated upon synapsis, as chromosome movements subside (*28*). Our observations indicate that the forces transmitted to the NE do not cease at this time but that the lamina restricts the movement of LINC complexes and pairing centers, presumably because dephosphorylation of LMN-1 leads to more extensive cross-linking.

**Fig. 3.**
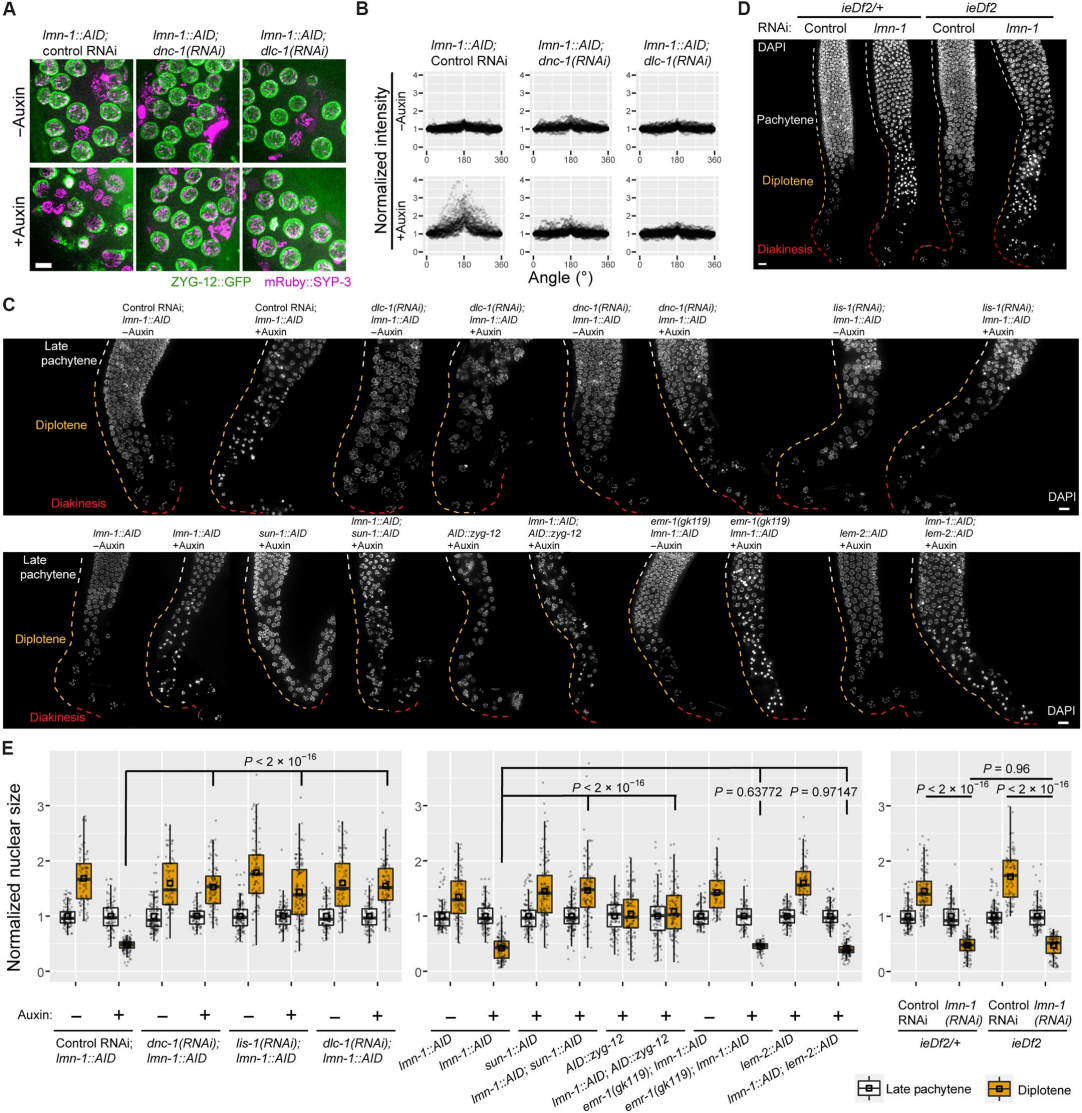
Nuclear collapse is rescued by disrupting dynein or LINC complexes, but not the connections between LINC complexes and pairing centers. (**A**) Composite images showing mRuby::SYP-3 (magenta) and ZYG-12::GFP (green) in diplotene nuclei of indicated genotypes or treatments. All images are maximum-intensity projections and were scaled identically. Scale bar, 5 μm. (**B**) Polarization of ZYG-12::GFP fluorescence intensities in diplotene nuclei, mapped to angular coordinates. The brightest point was designated as 180° and the intensity at 0° was normalized to 1 (see fig. S8B). For detailed statistics, see data S1. (**C**) Nuclear morphology at later stages of meiotic prophase. All worms were homozygous for *P_sun-1_::TIR1*. Colored dashed lines mark meiotic stages on the basis of the anatomical positions of gonads from control animals. Scale bars, 10 μm. (**D**) Nuclear morphology at later stages of meiotic prophase, from gonads dissected from animals of indicated genotypes or treatments. Pairing center proteins HIM-8, ZIM-1, ZIM-2, and ZIM-3 are expressed in *ieDf2/+* heterozygotes but absent in homozygotes. Colored dashed lines indicate meiotic stages on the basis of their distributions in controls. Scale bar, 10 μm. (**E**) Normalized nuclear size in worms of indicated genotypes or treatments. Medians (black crossbars) and means (black boxes) are shown. The colors correspond to dashed lines in (C) and (D). All statistics are in Materials and Methods and data S1.

During late prophase in LMN-1–depleted germ lines, we observed relocalization of SUN-1 and ZYG-12 to form an asymmetrical cap on many nuclei, shortly preceding nuclear collapse (Figs. 2F and 3A, fig. S8B, and movie S4). These NEs also often showed obvious deformations, indicative of active forces being exerted at these sites [see Fig. 2 (F and G) for examples]. By tracking individual nuclei over time, we observed that the nuclear volume shrank before a reduction in chromosomal volume (Fig. 2, F to I, and movies S4 and S5). This suggests that the collapse is driven by destabilization of the NE and associated proteins, rather than by compaction of the chromosomes.

### Dynein-mediated forces promote LINC complex polarization and nuclear collapse

Movement of LINC complexes in early meiosis is driven at least in part by their interaction with dynein motors moving along microtubules in the cytoplasm (*4*, *12*). In late prophase, dynein and ZYG-12 remain localized at the NE and play important roles in positioning of nuclei within oocytes, indicating that they continue to interact (*3*, *12*, *35*, *50*–*52*). We found that depletion of either dynein light chain DLC-1 or the dynein activator dynactin (DNC-1) by RNA interference (RNAi) largely eliminated the asymmetric localization of LINC factors during late prophase (Fig. 3, A and B, and data S1). Nuclear collapse induced by LMN-1 depletion was also rescued by co-depletion of DNC-1, DLC-1, or LIS-1 (LISsencephaly 1, another dynein activator), but not DYLT-1 [DYnein Light chain (Tctex type) 1], which is dispensable for some dynein functions (Fig. 3, C and E, and figs. S9 and S10) (*53*, *54*). Co-depletion of dynein heavy chain (DHC-1) or light intermediate chain (DLI-1) also prevented apparent collapse in diplotene, although nuclear positioning was markedly perturbed (fig. S9). Co-depletion of SUN-1 or ZYG-12 using auxin-inducible degradation (figs. S1B, S10, S11, and S12) also rescued nuclear collapse, further indicating that this process is driven by asymmetric forces acting on nuclei through the LINC complex (Fig. 3, C and E). Consistent with these findings, mutations in *sun-1* and *zyg-12* partially restore the sterility seen in *lmn-1*–null mutants (*33*).

Notably, co-depletion of SUN-1 did not suppress the elevated and persistent RAD-51 foci in animals depleted of both LMN-1 and SPO-11, reinforcing the conclusion that SPO-11–independent DNA damage does not trigger collapse (fig. S4). Notably, depletion of SUN-1 and its KASH partner ZYG-12 in the germ line had distinct effects on apoptosis. While depletion of either protein rescued nuclear collapse caused by LMN-1 depletion (Fig. 3, C and E), depletion of ZYG-12 led to elevated apoptosis, while depletion of SUN-1 did not (fig. S13, A and B). This was somewhat unexpected because loss of either protein causes synapsis defects and persistent DSBs (fig. S4, B and C, and fig. S13, C and D) (*3*, *40*). However, previous studies have also revealed different effects of *sun-1* and *zyg-12* mutations, e.g., on nuclear positioning in the germ line (*3*, *12*, *40*). Together, these observations indicate that SUN-1, but not ZYG-12, is required for damage-induced apoptosis during meiosis. We also observed that wild-type nuclei undergoing “physiological” apoptosis (*42*) have no ZYG-12 at their NEs but retain SUN-1 (fig. S13E). Despite this, SUN-1 is not required for physiological apoptosis, because the number of apoptotic nuclei was unaffected by SUN-1 depletion (fig. S13B). In contrast, prior evidence has suggested that SUN-1 is required for developmentally programmed cell death in *C. elegans* embryos (*55*), indicating that physiological cell death in the germ line is distinct from developmentally programmed apoptosis, although both depend on CED-4.

As described above, pairing centers interact with the NE during early meiotic prophase, when chromosomes undergo pairing and synapsis. These interactions require a family of four zinc finger proteins encoded by adjacent genes arranged in an operon (*56*). To test whether connections between pairing centers and the NE contribute to nuclear collapse, we used RNAi to deplete LMN-1 in a strain in which all four zing finger genes were deleted (*57*). We observed collapse of late prophase nuclei following LMN-1 depletion, indicating that asymmetric forces acting on late meiotic nuclei do not require attachment of LINC complexes to pairing centers (Fig. 3, D and E). We also observed nuclear collapse in the absence of MJL-1 (MAJIN-like-1), a recently identified INM protein that is essential for interactions between pairing centers and LINC complexes (fig. S14) (*58*). However, it is possible that nuclear collapse depends on chromosome-NE interactions mediated through other sites or mechanisms (*59*, *60*).

### The lamin meshwork cooperates with the INM proteins SAMP-1 and EMR-1/LEM-2 to stabilize oocyte nuclei

**Fig. 4.**
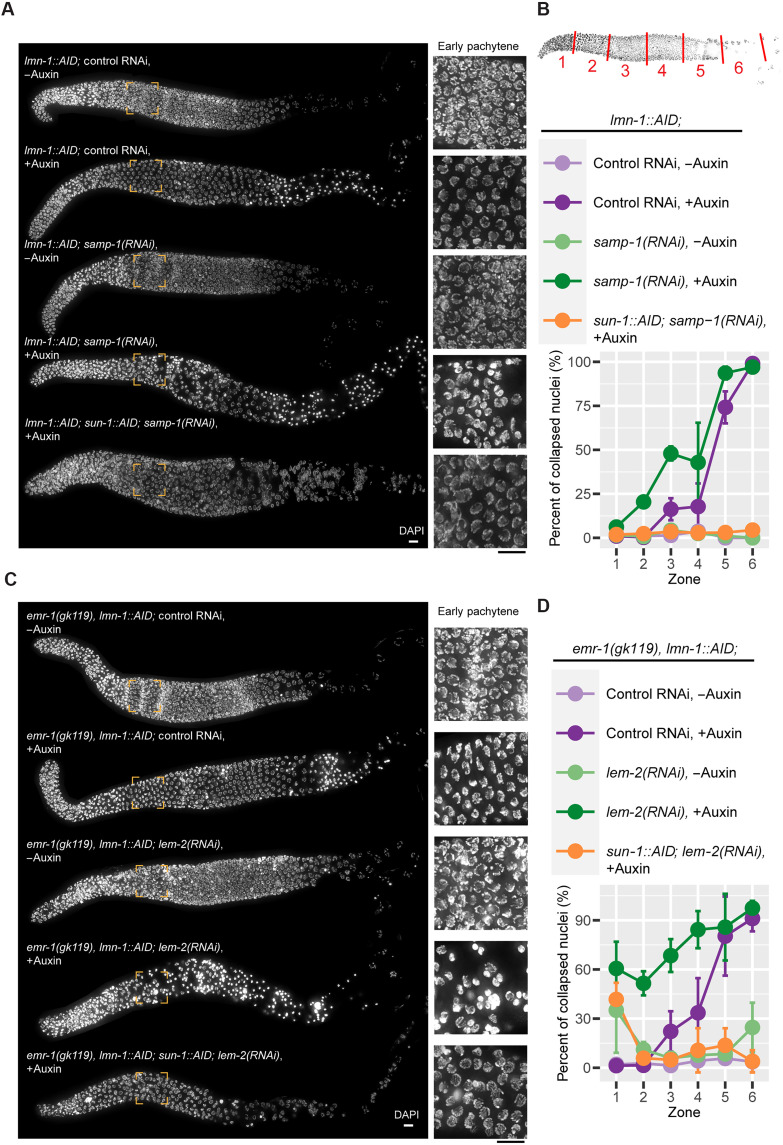
Nuclear collapse due to lack of LMN-1 is exacerbated by co-depletion of SAMP-1 or EMR-1/LEM-2. (**A** and **C**) Nuclear morphology throughout meiosis. All worms were homozygous for *P_sun-1_::TIR1* or *P_gld-1_::TIR1* (omitted in the figure). Scale bars, 10 μm. Insets for early pachytene are 3× magnified. (**B** and **D**) Fraction of collapsed nuclei as a function of meiotic progression. Schematic at the top shows distal gonad being divided into six zones of equal lengths, so that the percent of nuclei with collapsed/condensed chromatin in each zone can be quantified. Three worms were measured per condition. Means ± SD are plotted. Pairwise comparisons for proportions were used to compute the *P* values (adjusted by the Benjamini-Hochberg method; see data S1).

We also tested the effects of depleting three other NE proteins implicated in LINC-mediated mechanotransduction: human emerin homolog 1 (EMR-1), LEM domain protein 2 (LEM-2), and Spindle-Associated Membrane Protein 1 (SAMP-1) (*61*–*65*). Meiosis and germline nuclear morphology appeared normal following depletion of each of these three proteins, and their depletion did not rescue nuclear collapse caused by LMN-1 depletion (Fig. 3, C and E; Fig. 4, A and B; and figs. S10 and S15). However, depletion of SAMP-1 exacerbated the effects of LMN-1 depletion, in that collapsed nuclei were observed in a more distal (earlier) region of the germ line, as early as early pachytene (Fig. 4, A and B). Because LEM-2 and EMR-1 have partially overlapping functions (*66*), we tested the effects of co-depleting them and found that this also sensitized nuclei to LMN-1 depletion (Fig. 4, C and D, and fig. S16). LINC complexes were also more strongly polarized in LMN-1–depleted early pachytene nuclei when EMR-1 and LEM-2 (or SAMP-1) were co-depleted (fig. S17 and data S1). Co-depletion of SUN-1 rescued the earlier nuclear collapse, indicating a common cause of meiotic nuclear collapse during oogenesis (Fig. 4). Thus, we conclude that EMR-1/LEM-2 and SAMP-1 play LMN-1–independent roles in stabilizing nuclei against LINC-mediated forces.

### LMN-1 depletion reduces the stiffness of the oocyte NE

**Fig. 5.**
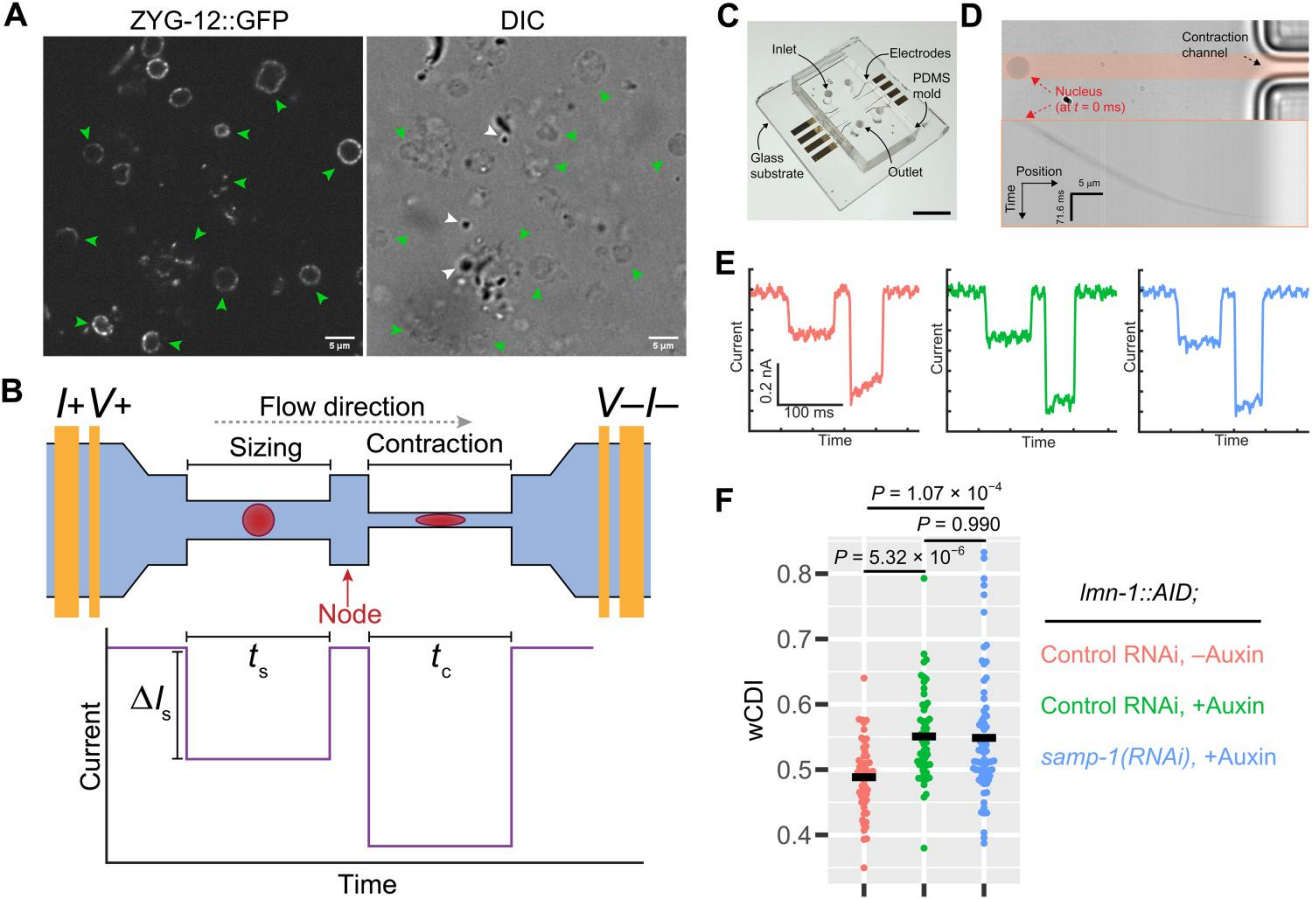
NE stiffness is reduced following LMN-1 depletion. (**A**) Meiotic nuclei isolated from adult hermaphrodites expressing ZYG-12::GFP. Green arrowheads indicate nuclei, and white arrowheads indicate debris. Scale bars, 5 μm. (**B**) The mechano-NPS platform consists of a microfluidic channel segmented by a node. A pair of electrodes at either end of the channel enables a four-terminal measurement of the current. Nuclear diameter is measured within the sizing segment, and nuclei are compressed under a constant applied strain in the contraction segment. The expected current pulse caused by a nucleus transiting the channel is shown. Δ*I*_S_ corresponds to the initial current drop caused by a nucleus transiting the sizing segment. *t*_s_ and *t*_c_ correspond to the transit time of a nucleus passing through the sizing and contraction segments, respectively. Nuclear size is determined by the magnitude of Δ*I*_S_ (eq. S1). Nuclear stiffness is determined by the transit time of a nucleus passing through the contraction segment (*t*_c_): stiffer nuclei take longer than softer ones. To normalize with respect to nuclear size, the whole-cell deformability index, wCDI, which is inversely related to the Young’s modulus, is used (eqs. S2 and S3). (**C**) A mechano-NPS device. The microfluidic channel corresponding to that shown in the schematic in (B) is located between the inlet and the outlet. Scale bar, 6 mm. (**D**) A nucleus about to enter the contraction segment. The pink shaded area was used to make the kymograph aligned below the snapshot. Scale bars, 5 μm and 71.6 ms. (**E**) Representative current pulses of a control (pink), LMN-1–depleted (green), and LMN-1 and SAMP-1 co-depleted (blue) nucleus transiting the sizing and the contraction segments. Scale bars, 100 ms and 0.2 nA. (**F**) Quantification of the wCDI. Black cross bars indicate means. All statistics are in Materials and Methods and data S1.

The nuclear lamina resists pushing forces from outside the NE (*16*, *21*). To test whether LMN-1 is important for the stiffness of meiotic NE, we adapted a mechanophenotyping assay to measure NE stiffness. Mechano-node-pore sensing (mechano-NPS) is a recently developed technique for measuring the stiffness of single cells (*67*, *68*). This method uses a four-terminal current measurement to detect the current pulse caused by a particle traversing across a microfluidic channel segmented by at least one “node” and squeezing through a “contraction” segment of the channel (Fig. 5B). Information on the particles’ biophysical properties, including diameter and stiffness, can be obtained through analysis of the recorded current pulses. We isolated meiotic nuclei from dissected gonads (mainly pachytene nuclei; Fig. 5A and fig. S18) and used mechano-NPS to measure their stiffness (Fig. 5, B to E). The whole-cell deformability index (wCDI) is a dimensionless parameter used in mechano-NPS and is inversely related to stiffness (*67*). Quantitative wCDI analyses of nuclei revealed that NE stiffness was significantly reduced following LMN-1 depletion. Co-depletion of LMN-1 and SAMP-1 did not reduce NE stiffness compared to LMN-1 depletion alone (Fig. 5F). Thus, SAMP-1 may not contribute directly to stiffness of the NE but may help to distribute LINC complexes along the nuclear surface to prevent nuclear disruption (fig. S17).

## DISCUSSION

**Fig. 6.**
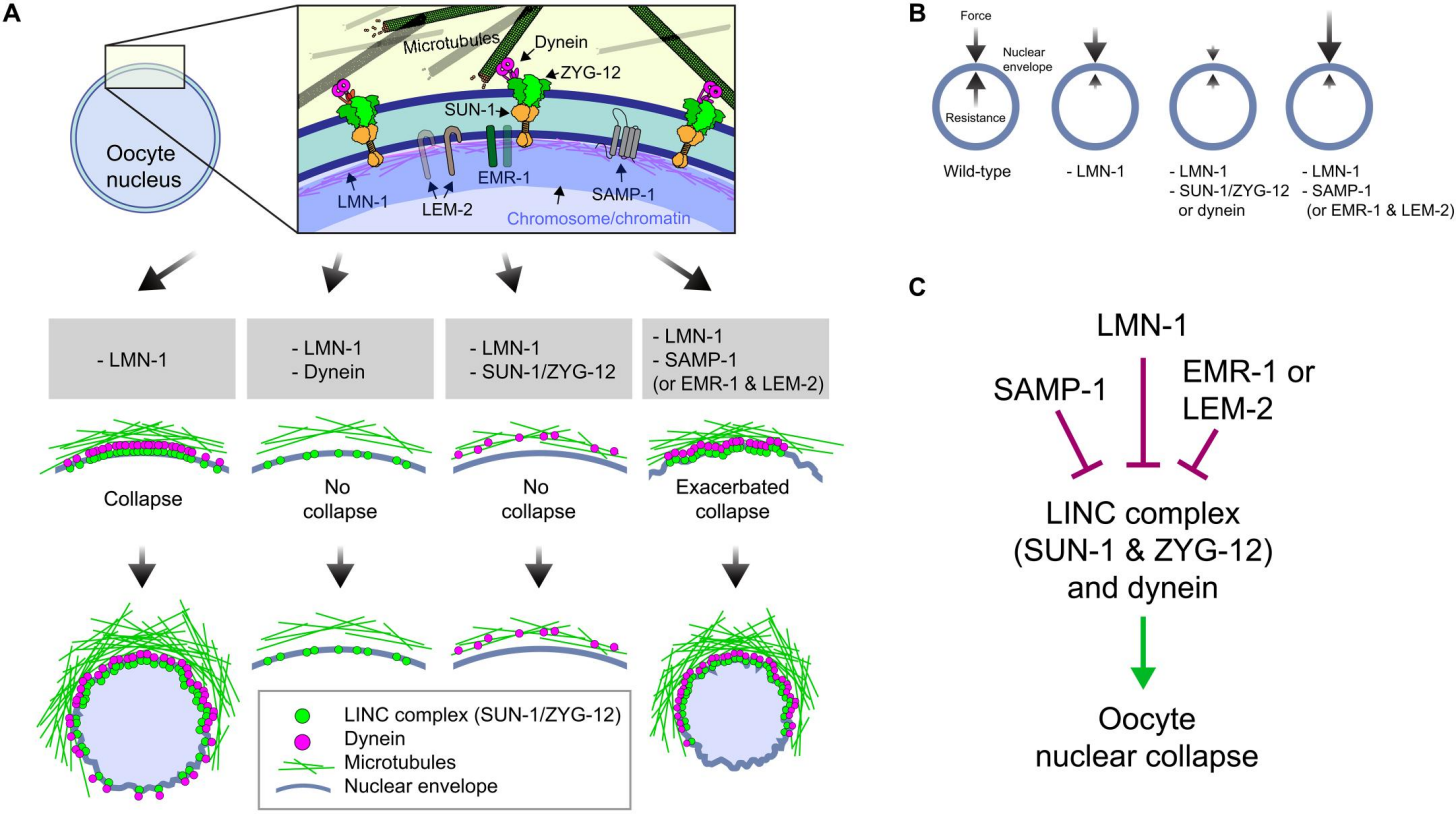
A cooperative network stabilizes oocyte nucleus against mechanical forces. (**A**) Illustration of components influencing nuclear stability during oogenesis. LMN-1 (magenta) and INM proteins (EMR-1, LEM-2, and SAMP-1) likely contribute to the mechanical stability of the nucleus either by providing rigidity at the INM that withstand forces generated by microtubule/dynein and transmitted by the LINC complex or by spreading LINC-mediated forces over a larger area of the NE. Depletion of LMN-1 leads to polarization of LINC complexes that transmits unbalanced forces, leading to nuclear collapse in diplotene. Co-depletion of components/regulators of the dynein motor, SUN-1 or ZYG-12, rescues nuclear collapse. Simultaneous depletion of EMR-1 and LEM-2 or of SAMP-1 leads to exacerbated, earlier nuclear collapse. (**B**) Force balance at the nuclear envelope. External pushing forces are depicted by arrows pointing inward to the nucleus, and resisting forces are depicted by arrows pointing outward from inside the nuclear envelope. Schematics show balanced forces across the NE in a wild-type nucleus and unbalanced forces due to LMN-1 depletion leading to diplotene collapse. Upon LMN-1 depletion, force balance can be restored by reducing LINC-mediated mechanotransduction. However, the imbalance can be exacerbated by co-depleting SAMP-1 (or EMR-1 and LEM-2), which likely causes increased forces applied on the NE from outside because of more polarized LINC complex distribution. (**C**) Regulation of nuclear stability during *C. elegans* oogenesis. Factors inhibiting collapse are depicted with blunt arrows, while those that promote collapse are depicted with pointed arrows.

Our data reveal that forces that can destabilize cell nuclei act throughout meiotic prophase, and a network of INM proteins cooperate with the nuclear lamina to enable successful reproduction by resisting these forces (Fig. 6). LMN-1–deficient nuclei collapse as oocytes undergo massive growth and cellularization, which lead to hydrodynamic destabilization (*69*, *70*). However, depletion of DNC-1 rescued nuclear collapse without impeding oocyte growth; similarly, depletion of abnormal OOCyte formation 5 (OOC-5) was found to rescue collapse without obvious effects on oocyte nuclear volume (*33*). Thus, growth alone is not sufficient to induce collapse, but growth-mediated destabilization may synergize with the effects of forces acting on the NE.

In the absence of LMN-1 and SAMP-1 or EMR-1/LEM-2, nuclei collapse during early meiosis. The lamina is thought to be destabilized at this stage of meiosis through phosphorylation of LMN-1, which makes the protein more sensitive to detergent extraction (*28*). In other metazoans, lamins are replaced by more labile isoforms or completely dismantled during early meiotic prophase to enable chromosome movements that promote homolog pairing and synapsis (*71*). Thus, lamin-independent mechanisms likely play a conserved role in reinforcing the NE during meiosis. While orthologs of EMR-1, LEM-2, and SAMP-1 have each been reported to interact with lamins (*64*, *72*–*75*), our findings that depletion of SAMP-1 did not make LMN-1–depeleted nuclei less stiff, yet resulted in more polarized LINC complex distribution, suggests that SAMP-1 either counteracts forces exerted by LINC complexes or somehow spreads these forces over a larger area of the nucleus (Fig. 6B).

In some cells, the lamina is important to anchor LINC complexes at the NE. For example, in migrating mouse fibroblasts, transmembrane actin-associated nuclear (TAN) lines containing LINC complexes are required for nuclear movement (*76*). In the absence of functional LMNA, TAN lines cannot be anchored properly in the NE and consequently “slip over” the nucleus, which fails to move (*77*). This seems analogous to the hyperpolarized LINC complex “cap” we observe at the NE following LMN-1 depletion. Our observations are also consistent with previous findings that a disease-causing *lmn-1* allele leads to dynein-dependent NE rupture during pronuclear migration in *C. elegans* (*78*); that defective nuclear positioning caused by progerin (the pathological prelamin A variant that causes Hutchinson-Gilford progeria syndrome) can be rescued by inhibiting dynein or reducing SUN-1–mediated microtubule-nuclear connections (*79*); and that NE rupture, viability, and function of *Lmna* knockout myotubes in mice can be rescued by reducing microtubule-based force via depletion of a kinesin or expression of a dominant-negative KASH protein (*25*). These observations support the idea that the nuclear lamina is important to stabilize nuclei by resisting microtubule/LINC-mediated forces.

Oocyte nuclei experience a range of unusual forces, including those arising from chromosome movements during meiosis, large-scale cell and nuclear growth during maturation, and extracellular forces both within the ovarian cortex and during ovulation. In mice, compressive forces from the surrounding granulosa cells are important to maintain the dormant state of oocytes arrested at the diplotene (dictyate) stage, through dynein-based nuclear rotation (*15*). Given that LINC complexes are required for nuclear rotation (*5*, *80*) and our finding that the lamina enables LINC proteins to resist hyperpolarization because of dynein-based motility, the nuclear lamina may be important for maintaining the dormant state of primordial oocytes in mammals. Polymorphisms in the emerin-associated NE protein Nemp1, which confers stiffness to the NE, have been associated with early menopause in humans. Nemp1 is also essential for fertility across metazoans, including in *C. elegans* (*16*), adding another component to the network of factors that contribute to NE integrity in oocytes. Our work adds to a growing body of evidence that multiple pathways contribute to the resilience of oocyte nuclei during metazoan reproduction.

## MATERIALS AND METHODS

### Generation of worm strains

All *C. elegans* strains were maintained at 20°C under standard conditions (*81*). Details of alleles generated in this study are listed in table S2. A complete list of *C. elegans* strains generated in this study is also provided in table S3. All alleles were generated using CRISPR-Cas9 genome editing [Alt-R CRISPR-Cas9 guide RNA (gRNA) products, Integrated DNA Technologies (IDT)], as previously described (*82*). Briefly, duplexed gRNA-Cas9 ribonucleoprotein complex was injected into the gonad of young adults, with *dpy-10* co-CRISPR to facilitate the screen of edited progeny (*83*, *84*). Custom-designed CRISPR RNAs (crRNAs) targeting genes of interest (200 μM; IDT; sequences can be found in table S4) were mixed with crRNA targeting *dpy-10* (50 μM) at 3:1 (v:v) ratio and then combined with transactivating crRNA (tracrRNA) (200 μM; IDT) at 1:1 (v:v) ratio, annealed at 95°C for 5 min followed by room temperature for 5 min. The duplexed gRNAs (2 μl) were then complexed with Cas9-NLS (40 μM; UC Berkeley MacroLab; 4 μl) at room temperature for 5 min. Repair templates, including dsDNA repair template [purified polymerase chain reaction (PCR) product of gBlock from IDT] and single-stranded DNA repair template for *dpy-10* (IDT), were added to the injection mix (10 μl of final volume), which was centrifuged at 13,200 rpm in 4°C for 10 min before loading quartz needles (Sutter Instrument) for microinjection (Eppendorf). Three to four injected hermaphrodites per plate were incubated at 20°C for 4 days, before individual F_1_ Dumpy or Roller progeny were transferred to fresh plates. After 3 days at 20°C, the F_1_ hermaphrodite from each plate was lysed and genotyped by PCR.

Tagging the N or C terminus of endogenous LMN-1 with the auxin-inducible degron (AID) resulted in a loss-of-function phenotype. N-terminal tagging of SUN-1 with the degron sequence also resulted in 100% inviable progeny. C-terminal tagging of SUN-1 with AID did not disrupt SUN-1 function, but the protein was refractory to auxin-inducible degradation. Tagging of both LMN-1 and LEM-2 in *emr-1(gk119)* mutants resulted in embryonic lethality.

### Fertility and viability

Brood size, viability, and males among the self-progeny of individual hermaphrodites were quantified as described (*82*). Briefly, L4 hermaphrodites (P_0_) were plated individually onto freshly spread, thin lawns of OP50 on 60-mm nematode growth medium (NGM) plates. Animals were transferred to a fresh plate every 24 hours for a duration of 4 to 5 days until egg-laying ceased. Eggs were counted after removal of the parents, and viable progeny were counted 2 to 3 days later. To quantify brood size, viability, and male self-progeny in the presence of auxin, age-matched hermaphrodites at L1 or L4 stages were transferred onto auxin plates following the same procedures. When counting eggs on non-auxin plates, only those eggs that appeared firm with intact eggshells were counted; when counting eggs on auxin plates, however, because of the many dead and deformed eggs, only eggs with intact eggshells were counted.

### Auxin-induced degradation

Plates containing 2 mM IAA (auxin) were prepared as previously described (*82*). An overnight culture of OP50 in LB was concentrated 10-fold and supplemented with auxin before seeding the auxin plates. Unless otherwise specified, hermaphrodites were picked at the L4 stage to regular plates and transferred 12 hours later to plates containing 2 mM IAA (auxin) to initiate degradation or to control plates lacking auxin. Worms were left on auxin plates for 12 hours before dissection (for immunofluorescence) or live imaging (of acridine orange–stained nuclei in live animals or in vivo imaging of fluorescent proteins). Auxin-induced co-degradation (of AID-tagged LMN-1 and other proteins) was carried out under identical conditions as single degradation (of AID-tagged LMN-1 alone), and both showed equal efficiency in LMN-1 depletion, ruling out that any rescue by co-degradation was due to incomplete LMN-1 depletion (fig. S12). All experiments were performed at 20°C.

### RNA interference

RNAi experiments were carried out according to established protocols with minor modifications (*85*, *86*). Briefly, frozen stocks of HT115 bacteria carrying L4440 constructs from the Ahringer *C. elegans* RNAi feeding library (see table S5) (*87*, *88*) were streaked for individual colonies onto 10-cm LB agar plate supplemented with carbenicillin (final concentration of 100 μg/ml) and tetracycline hydrochloride (topically spread; 2 μl of 25 mg/ml stock aqueous solution diluted in 400 μl of LB). RNAi constructs used in this work were validated by Nanopore whole-plasmid sequencing (Primordium). A single colony was inoculated into 5 ml of LB supplemented with carbenicillin (final concentration of 50 μg/ml) and tetracycline hydrochloride (final concentration of 12.5 μg/ml) and cultured for about 16 hours overnight at 37°C at 250 rpm. NGM agar plates for RNAi were made with the following supplements freshly added before pouring: carbenicillin (final concentration of 50 μg/ml), tetracycline hydrochloride (final concentration of 12.5 μg/ml), and isopropyl-β-d-thiogalactopyranoside (IPTG; final concentration of 1 mM). Auxin was also added to plates for experiments combining RNAi and auxin-inducible degradation. These RNAi plates were seeded with 300 μl of overnight bacterial culture, allowed to dry at room temperature, and moved to a dark 30°C incubator for 48 hours for IPTG induction. All RNAi plates were stored at 4°C, protected from light. HT115 bacteria carrying empty L4440 vectors were used as RNAi controls. For RNAi experiments, L4 hermaphrodites were transferred onto RNAi plates and dissected 48 hours later; when combined with inducible degradation, animals were transferred to plates containing both IPTG and auxin for 12 hours before dissection. All experiments were performed at 20°C. Effective depletion of target proteins were confirmed by immunofluorescence (e.g., LEM-2 and LMN-1), live imaging of worms expressing fluorescently tagged target proteins (e.g., DNC-1::GFP) (*89*), or phenotypes consistent with known essential functions (e.g., dynein components or regulatory proteins). For SAMP-1 depletion, *samp-1(RNAi)* was used to restrict the effects to the adult germ line. We did not observe embryonic lethality following *samp-1(RNAi)*.

### Immunofluorescence

Immunofluorescence was carried out as previously described (*90*) with minor modifications. Dissected gonads were transferred to low-retention microcentrifuge tubes, and subsequent steps (e.g., fixation, permeabilization, blocking, and staining) were performed in suspension. The following antibodies were used: anti-V5 (mouse, P/N 46-0705, Invitrogen), anti-hemagglutinin (HA) (mouse monoclonal, #26183, Invitrogen), anti-GFP (1:400; mouse monoclonal, Roche), anti–SYP-1 [1:400; goat, affinity purified (*57*)], anti-HTP-3 [1:400; chicken (*91*)], anti-HIM-8 [1:400; rat (*92*)], anti-NPP-7 (1:1000; rabbit polyclonal, SDIX, SDQ0870), anti-NPP-10 (1:1000; rabbit polyclonal, SDIX, SDQ0828), anti–RAD-51 [1:20,000; rabbit polyclonal, SDIX, catalog no. 29480002 (*82*)], and secondary antibodies conjugated to Alexa Fluor 488, Cy3, or Cy5/AF647 (1:400; Jackson ImmunoResearch or Life Technologies).

All images of fixed samples were acquired using a DeltaVision Elite system (GE) equipped with a 100× 1.45 numerical aperture (NA) oil immersion objective (Olympus). Wide-field images were deconvolved using the SoftWoRx Suite (Applied Precision, GE). To compare between individual gonads or slides, the same exposure times were used for all samples. All fixed gonads were imaged as three-dimensional (3D) image stacks at intervals of 0.2 μm. Maximum intensity projections of deconvolved 3D images were assembled using Fiji and Illustrator (Adobe) for figures, unless otherwise noted.

### Acridine orange assay

Acridine orange staining and washing were carried out according to established protocols with minor modifications (*93*). The assay was timed so that the entire duration of auxin treatment was consistent with the other auxin-induced degradation experiments (i.e., the time required for acridine orange staining and washing was included in the total time of auxin treatment). Briefly, acridine orange staining solution was prepared by diluting stock solution (10 mg/ml; Molecular Probes, A-3568) 1:200 into M9 buffer. Age-matched adult hermaphrodites from control or auxin plates were picked to the middle of OP50 lawn of fresh control and auxin plates. A total of 900 μl of staining solution was gently pipetted onto each plate until the entire surface (including the OP50 lawn) was covered. Animals remained on top of the OP50 lawn to ensure active feeding behavior, which is important for the uptake of acridine orange. Plates were incubated for 1 hour at room temperature in the dark. Worms were then washed off the plate with 1.5 ml of M9 buffer, transferred to low-retention 1.5-ml tubes (Fisherbrand), and pelleted by a low-speed spin (10 s) in a tabletop mini centrifuge. The supernatant was removed and replaced with M9 buffer. Centrifugation and washing were repeated twice more. After the final wash, the worms were transferred to fresh control or auxin plates and kept in the dark for an additional 45 min at room temperature before mounting on agarose pads. They were imaged within 1 hour, using a spinning disk confocal microscope equipped with differential interference contrast (DIC) and 488-nm excitation laser to image and score acridine orange–positive apoptotic nuclei in the germ line (see the “Live imaging” section). Low retention pipette tips (Fisherbrand) were used throughout the washing process.

### Live imaging

For live imaging, gravid hermaphrodites were picked into a 10-μl drop of water containing 250 μM tetramisole hydrochloride on a freshly prepared agarose pad (7.5% in water). The immobilized worms were overlaid with no. 1.5 high-precision cover glasses (0.170 ± 0.005 mm; Marienfeld), sealed to the slide using VALAP [1:1:1 (w:w:w) vaseline:lanolin:paraffin] and imaged immediately. Image acquisition was carried out on a Marianas spinning disk confocal microscope (Intelligent Imaging Innovations Inc.) at ambient temperature (21°C), using a 100× 1.46 NA oil immersion objective. To analyze LINC complex mobility, 3D image stacks at specific gonad zones/meiotic stages were acquired every 5 s for a duration of 30 to 60 time points, with 10 *z* sections at intervals of 0.5 μm per time point. To examine the dynamics of diplotene nuclear collapse, image stacks were acquired every 10 s. DIC was used to facilitate identification of gonad regions. The 488- or 561-nm excitation lasers were used with identical parameters for control and experimental samples. In some cases, we also mounted animals using 100-nm polystyrene beads (Polysciences, catalog no. 00876) and serotonin creatinine sulfate (Sigma-Aldrich) as previously described (*49*) but observed no differences for the short-term imaging used in this work.

### Image analysis

To quantify nuclear volume (fig. S3) based on 4′,6-diamidino-2-phenylindole (DAPI) fluorescence, the “Surface” function in Imaris (×64, 9.2.0, Bitplane) was used for 3D segmentation and quantification. NE staining was used to confirm that segmented regions belong to the same nucleus (figs. S2 and S3).

Quantification of the distribution of LINC complexes at the NE was performed using the “freehand line” and “plot profile” tools in Fiji. Motion of LINC complex patches/foci in 3D was analyzed in Imaris (×64, 9.2.0, Bitplane) using the “Spots” function for tracking and reference frames for drift correction. The quantification of shrinking dynamics of collapsing NE and SC was carried out in Fiji, and shrinking speeds were computed using the linear model in RStudio.

To quantify the fraction of nuclei showing homolog pairing and complete synapsis as a function of meiotic progression, images of each gonad spanning the premeiotic (distal) end to early diplotene were divided into five zones of equal length (in figs. S4C and S6C). X-chromosome pairing was scored on the basis of the number of HIM-8 foci per nucleus, and complete synapsis was scored by colocalization of SYP-1 and HTP-3. The fraction of collapsed nuclei as a function of meiotic progression was scored similarly, except that the gonad region from the distal tip to early diakinesis was divided into six regions (Fig. 4, B and D). Collapsed nuclei were identified on the basis of smaller volume and hypercondensed chromatin compared to adjacent nuclei in the same zone from the same animal, as well as nuclei in the same zone from control animals without RNAi or auxin treatment.

### Measurement of nuclear stiffness

Meiotic nuclei were isolated from dissected adult *C. elegans* germ lines as previously described (*94*), with minor modifications. For each experimental group, at least 100 age-matched adult hermaphrodites were dissected in 1× Egg Buffer with Tween [25 mM Hepes (pH 7.4), 118 mM NaCl, 48 mM KCl, 2 mM EDTA, 0.5 mM EGTA, and 0.05% Tween 20]. Gonads were released by nicking with a scalpel blade. The tissue was transferred to 1× Wash Buffer [WB; 25 mM Hepes (pH 7.5), 118 mM NaCl, 48 mM KCl, and freshly supplemented with 0.5 mM spermidine, 0.05% Tween 20, and cOmplete protease inhibitor cocktail (Roche), one tablet per 10 ml of WB]. Extruded gonads were aspirated using a capillary glass pipette (quartz glass; item no: Q100-70-10, Sutter Instrument) controlled using a syringe (5 ml; BD) to separate this tissue from the worm carcasses (gonads were broken at approximately late pachytene/diplotene). Isolated gonads were then pooled using a regular pipette into 1 ml of freshly prepared 1× WB prechilled on ice and homogenized using a 1-ml Dounce homogenizer (Wheaton), using 33 strokes with the loose pestle followed by 33 strokes with the tight pestle. The resulting homogenate (approximately 1 ml) was then transferred to a low-retention 1.5-ml tube, vortexed at low speed for 1 min, split into two equal volumes, and each filtered through a 15-μm cell strainer (pluriSelect, reference no. 43-50015-01). The collected nuclear suspension was centrifuged at 3100 rpm for 10 min at 4°C, and the supernatant was collected and centrifuged again at 12,000 rpm for 2 min at 4°C. The pelleted nuclei were combined and resuspended in a total volume of about 50 μl of 1× WB. Low-retention 1.5-ml microcentrifuge tubes (Genesee Scientific, catalog no. 28-282LR) were used throughout nuclear isolation. Isolated nuclei were examined by loading a 5-μl droplet of the final resuspended solution on a glass-bottom dish (MatTek, P35G-1.5-14-C) followed by imaging using a spinning disk confocal microscope (see the “Live imaging” section). For mechano-NPS measurements, nuclei were suspended in 1× WB at a concentration of at least 4 × 10^4^ nuclei/ml.

Mechano-NPS was performed as previously described (*67*, *68*, *95*). The platform consisted of a 9.9-μm-high microfluidic channel embedded in a polydimethylsiloxane (PDMS; Sylgard 184, Dow Corning) mold that was oxygen plasma–bonded to a glass substrate with predefined platinum electrodes and gold contact pads (Fig. 5C). A node (80.4 μm by 50.4 μm, *L* × *W*) segmented the channel into two sections: a “sizing” segment (700.4 μm by 5.9 μm, *L* × *W*) that measured the size of the nucleus transiting the device and a contraction segment (700.4 μm by 2.9 μm, *L* × *W*) that applied a constant strain, ε = (*d*_n_ − *w*_c_)/*d*_n_, where *d*_n_ is the diameter of the nucleus and *w*_c_ is the width of the contraction channel, to the nuclei. For the results reported in Fig. 5F, the average applied strain was ε_avg_ = 0.15. Two sequential filters, 9.4 and 5.9 μm wide, respectively, were included at the microfluidic channel’s inlet to remove debris larger than the size of the nuclei. To create the PDMS mold, soft lithography was used as previously described (*67*, *68*, *95*), with one modification. After fabricating the negative-relief master onto a polished silicon (Si) wafer, we silanized the surface by drop casting 100 μl of hexamethyldisilazane (HMDS; Sigma-Aldrich) onto the wafer and spinning at 2500 rpm for 30 s. This silanization step was necessary to prevent small, critical features of the cured PDMS mold from ripping off when it was excised from the negative-relief master. Once the HMDS was air-dried, PDMS (10:1 prepolymer:curing agent, degassed) was subsequently poured onto the wafer and cured at 80°C for 2 hours. To determine the effective diameter of the sizing segment, polystyrene beads, 2 ± 0.05 μm (Sigma-Aldrich, 80177-5ML-F), were measured (table S6).

A constant DC voltage (6 V) across the completed mechano-NPS channel was used to measure the current pulses caused by nuclei transiting the channel when a nonpulsatile pressure of 7 kPa was used. A custom-written software (sohnlab/mechano-NPS-small: Version 1.0; https://doi.org/10.5281/zenodo.7884859) applied rectangular smoothing and down-sampling to the raw current versus time data and extracted the following measurements from each nucleus event: the magnitude of the initial current subpulse, Δ*I*_S_; the transit time across the sizing segment, *t*_S_; and the transit time across the contraction segment, *t*_c_ (Fig. 5B). Nuclear size and wCDI were calculated using eqs. S1 to S3. Nuclei with diameters smaller than, or equal to, the contraction channel width were not included in wCDI measurements, as ε = 0 for these nuclei (Fig. 5F and fig. S18B). For each treatment group, a sample size of *n* ≥ 53 nuclei was measured and used for wCDI comparison (Fig. 5F). Welch analysis of variance (ANOVA) tests with Games-Howell post hoc multiple comparisons tests were used to determine whether significant differences between treatment groups were present. A power analysis demonstrated that our sample sizes of *n* = 53, *n* = 53, and *n* = 71 nuclei for the control RNAi, −Auxin group; the control RNAi, +Auxin group; and the *samp-1(RNAi)*, +Auxin group, respectively, have sufficient power to detect the differences between the LMN-1 depleted group and the control (power > 0.99), as well as the LMN-1, SAMP-1 co-depleted group, and the control (power > 0.98). Additional details of the power analysis can be found in data S1. Isolated nuclei entering the contraction segment were filmed using a Nikon TE-2000-E microscope, a 60× objective (Nikon, Plan Fluor ELWD, 60× 0.70 NA), and a high-speed camera (Fastec IL5-S: SXGA High Speed Digital Camera) at 611 frames/s.

### Statistics

All statistical analyses were carried out in RStudio (version 1.2.5033). Ten meiotic nuclei per stage from each of three animals were measured per condition (without or with auxin) (Fig. 2, B and C). Three animals were measured per condition per stage: TZ, transition zone (*n* = 1132 detected particles for “−Auxin”; *n* = 859 for “+Auxin”); MP, mid-pachytene (*n* = 620 detected particles for “−Auxin”; *n* = 1657 for “+Auxin”); LP, late pachytene (*n* = 306 detected particles for “−Auxin”; *n* = 1675 for “+Auxin”) (Fig. 2E). Ten collapsing nuclei from eight LMN-1–depleted animals were measured (Fig. 2, H and I). To quantify asymmetry of LINC complex at diplotene, mean normalized peak intensities about 180° (170° to 190°) were compared using the two-tailed *t* test (Fig. 3B). Unpaired two-sample two-sided *t* test was used to calculate *P* values comparing normalized peak intensities from 170° to 190° between each condition (Fig. 3B): *P* < 2.2 × 10^−16^ between control RNAi and −/+ Auxin (66.42% increase in mean when +Auxin), *P* = 0.2603 between *dnc-1(RNAi)* and −/+ Auxin (1.26% decrease in mean when +Auxin), and *P* = 2.716 × 10^−6^ between *dlc-1(RNAi)* and −/+Auxin (3.85% increase in mean when +Auxin). To quantify LINC complex asymmetry at early pachytene NE (fig. S17, B and D), normalized intensity across 0° to 360° were combined as 0° to 180° because of the symmetric distribution about 180°, and the data were fit to a linear model (two-way ANOVA). All two-sample tests are unpaired and two-sided. Mann-Whitney test was used for calculating *P* values in Figs. 1F and 2I. One-way ANOVA and post hoc pairwise *t* tests (two-sided; adjusted by the Benjamini-Hochberg method) were used for calculating *P* values in Figs. 1H and 3E. Two-way ANOVA was used to calculate the *P* values in Fig. 2 (B, C, and E). Brown-Forsythe and Welch ANOVA tests with Games-Howell post hoc multiple comparisons test (two-sided) were performed to compute the *P* values in Fig. 5F, where sample sizes for the control RNAi, −Auxin; control RNAi, +Auxin; and *samp-1(RNAi)*, +Auxin are *n* = 53, *n* = 53, and *n* = 71 nuclei, respectively. All statistics can be found in data S1. ***P < 0.001, **P < 0.01, and *P < 0.05; ns (not significant), *P* ≥ 0.05.

## Supplementary Materials

### This PDF file includes:

Figs. S1 to S18 Tables S1 to S7 Legends for movies S1 to S5 Legend for Data S1 References

## Notes

View/request a protocol for this paper from *Bio-protocol*.
