# Supplementary material for: A cooperative network at the nuclear envelope counteracts LINC-mediated forces during oogenesis in C. elegans
Source: Sci Adv. 2023 Jul 12;9(28):eabn5709. doi: 10.1126/sciadv.abn5709 (PMC10337908; doi:10.1126/sciadv.abn5709)
Supplement: Supplementary file 1 — Figs. S1 to S18 Tables S1 to S7 Legends for movies S1 to S5 Legend for Data S1 References [file sciadv.abn5709_sm.pdf]

Science Advances
AAAS

Supplementary Materials for

A cooperative network at the nuclear envelope counteracts LINC-mediated forces during oogenesis in *C. elegans*

Chenshu Liu *et al.*

Corresponding author: Chenshu Liu, chenshu.liu@berkeley.edu; Abby F. Dernburg, afdernburg@berkeley.edu

Sci. Adv. **9**, eabn5709 (2023)
DOI: 10.1126/sciadv.abn5709

The PDF file includes:

Figs. S1 to S18
Tables S1 to S7
Legends for movies S1 to S5
Legend for Data S1
References

Other Supplementary Material for this manuscript includes the following:

Movies S1 to S5
Data S1

Fig. S1.

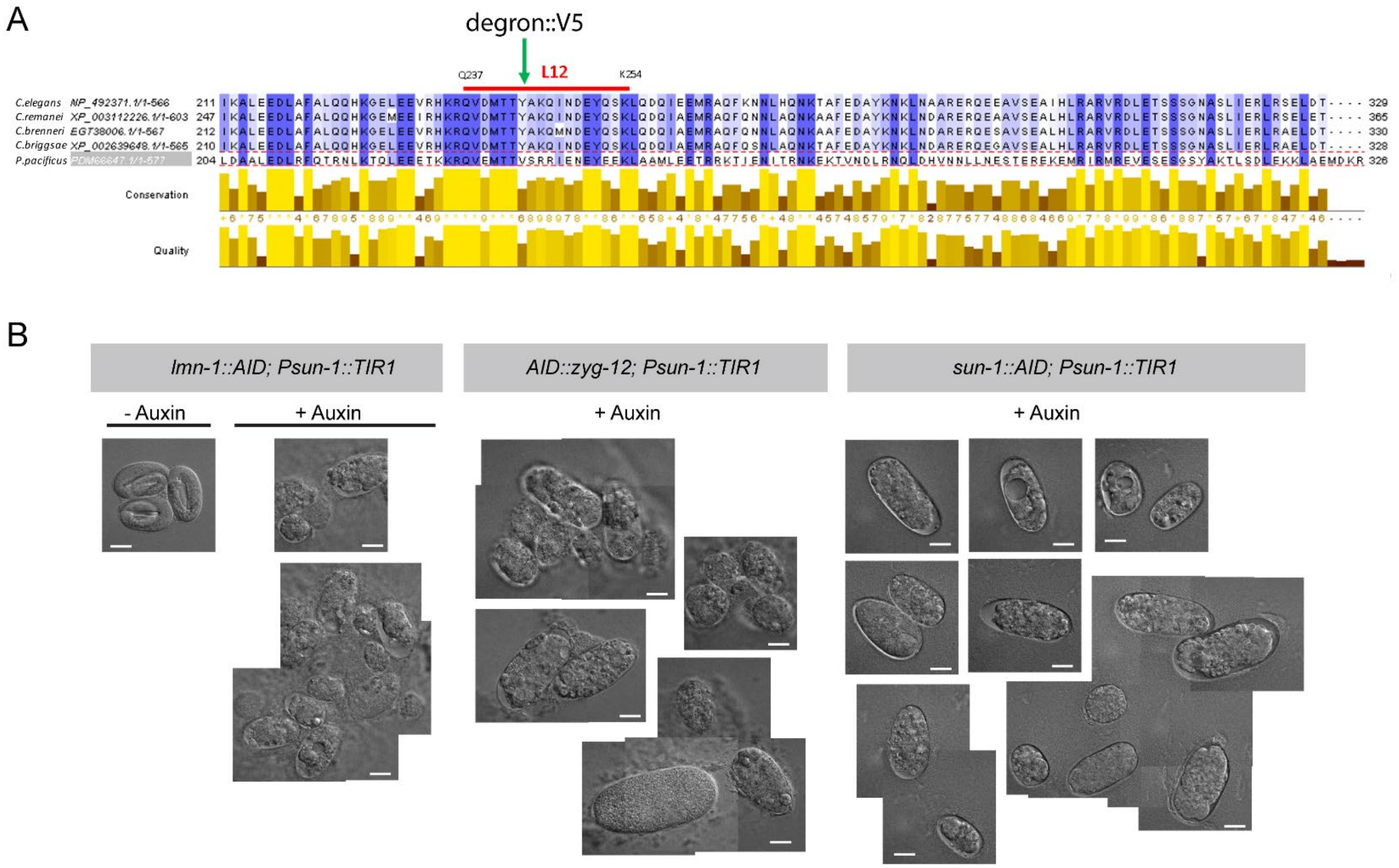
A
degron::V5
Q237
L12
K254
C.elegans NP_492371.1/1-566
C.remanei XP_003112226.1/1-603
C.brenneri EGT38006.1/1-567
C.briggsae XP_002639648.1/1-565
P.pacificus
Conservation
Quality
B
lmn-1::AID; Psun-1::TIR1
- Auxin
+ Auxin
AID::zyg-12; Psun-1::TIR1
+ Auxin
sun-1::AID; Psun-1::TIR1
+ Auxin

Fig. S1. (Related to Figs. 1 and 3) CRISPR tagging and auxin-inducible degradation of LMN-1 in *C. elegans* germline. (**A**) Multiple sequence alignments of nematode LMN-1 proteins were generated using Clustal Omega and visualized using Jalview, showing the position of the degron/V5 insertion in *C. elegans* LMN-1. (**B**) Morphology of eggs/embryos laid by hermaphrodite worms treated ± auxin for 48 hrs (from young adulthood). Images acquired using DIC. Scale bar, 20 μm.

Fig. S2.

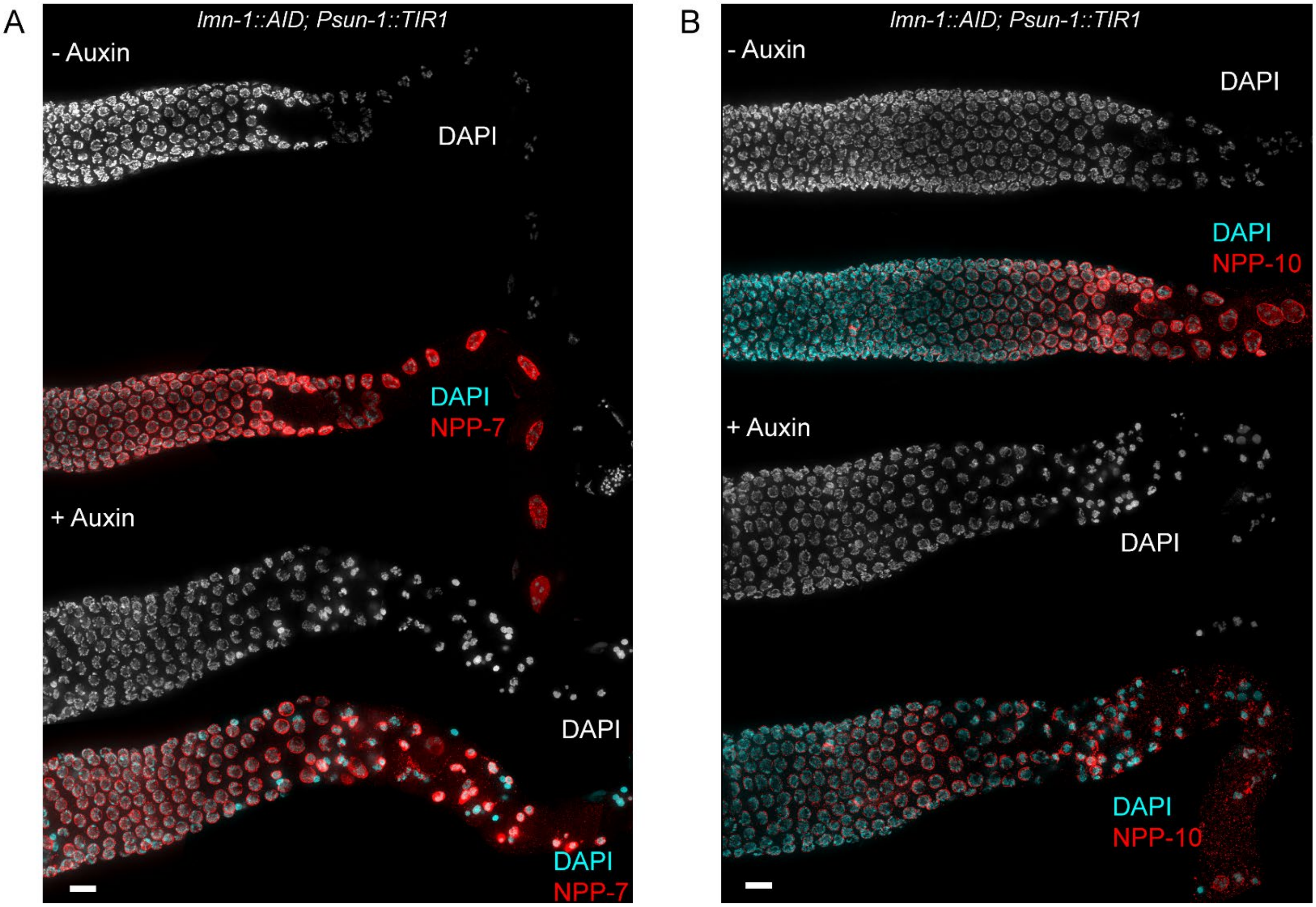
A
lmn-1::AID; Psun-1::TIR1
- Auxin
DAPI
DAPI
NPP-7
+ Auxin
DAPI
DAPI
NPP-7
B
lmn-1::AID; Psun-1::TIR1
- Auxin
DAPI
DAPI
NPP-10
+ Auxin
DAPI
DAPI
NPP-10

Fig. S2. (Related to Fig. 1) Representative images showing co-staining of DAPI and nucleoporins NPP-7 (A) or NPP-10 (B). Composite images are maximum-intensity projections. Meiosis progresses from left to right. Scale bars, 10 μm.

Fig. S3.

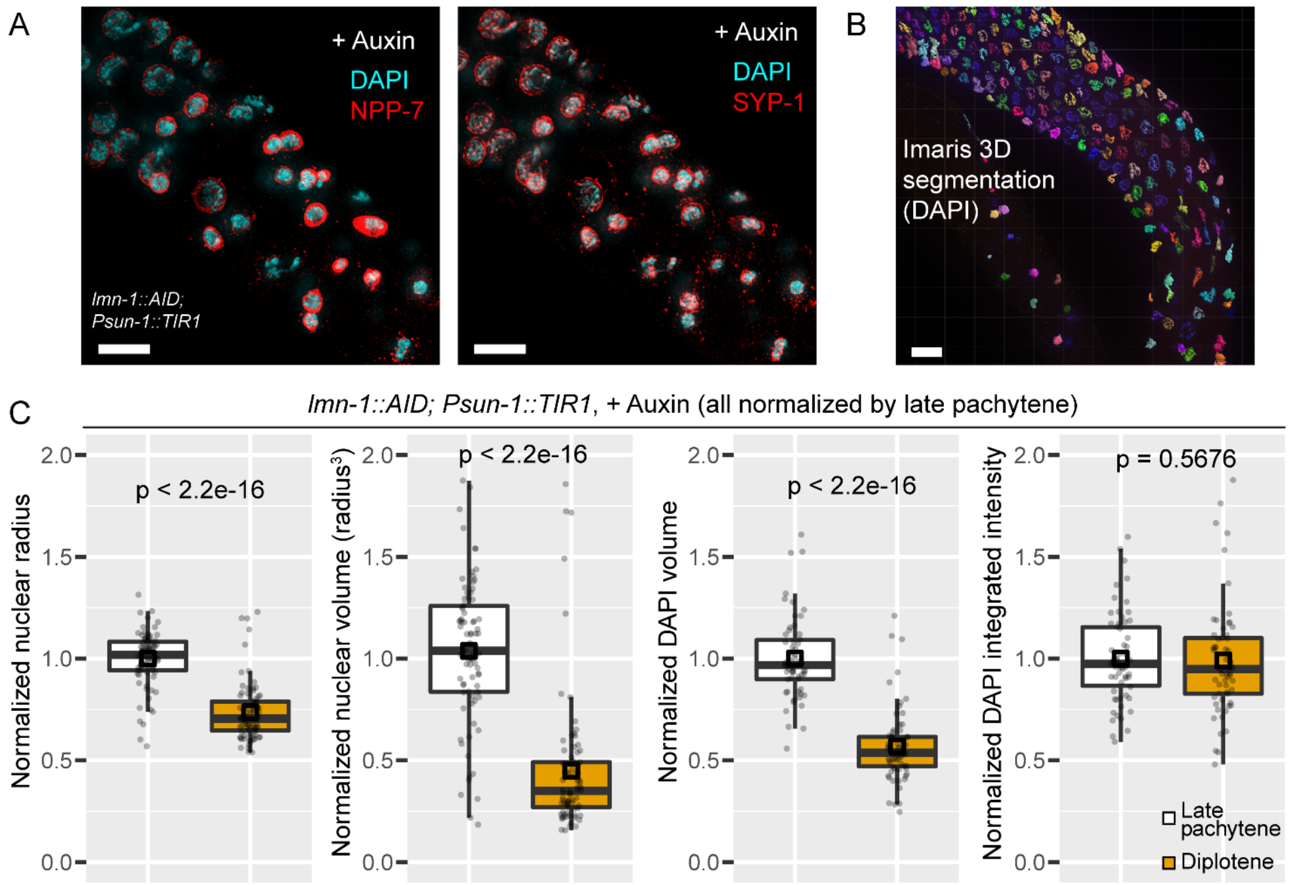
A
+ Auxin
DAPI
NPP-7
lmn-1::AID;
Psun-1::TIR1
+ Auxin
DAPI
SYP-1
B
Imaris 3D
segmentation
(DAPI)
C
lmn-1::AID; Psun-1::TIR1, + Auxin (all normalized by late pachytene)
Normalized nuclear radius
p < 2.2e-16
Normalized nuclear volume (radius³)
p < 2.2e-16
Normalized DAPI volume
p < 2.2e-16
Normalized DAPI integrated intensity
p = 0.5676
2.0
1.5
1.0
0.5
0.0
Late pachytene
Diplotene

Fig. S3. (Related to Fig. 1) Quantifying nuclear collapse. (**A**) Composite images (maximum-intensity projections) showing nuclei in late meiotic prophase stained for DAPI (cyan), NPP-7 (red) or SYP-1 (red). Meiosis progresses from top left to bottom right. Scale bars, 10 µm. (**B**) Segmentation of chromosome volume based on DAPI fluorescence. Meiosis progresses from top left to bottom right. Scale bar, 10 µm. (**C**) Quantification of nuclear radii based on NPP-7 or SYP-1 staining (with maximum Z projection), nuclear volume based on radius^3, nuclear size based on DAPI volume and integrated DAPI fluorescence intensity per nucleus. Nuclear sizes in late pachytene were normalized to one. Unpaired two-sample two-sided *t*-test was used to calculate *p*-values. At least 58 nuclei from three animals were analyzed per stage.

Fig. S4.

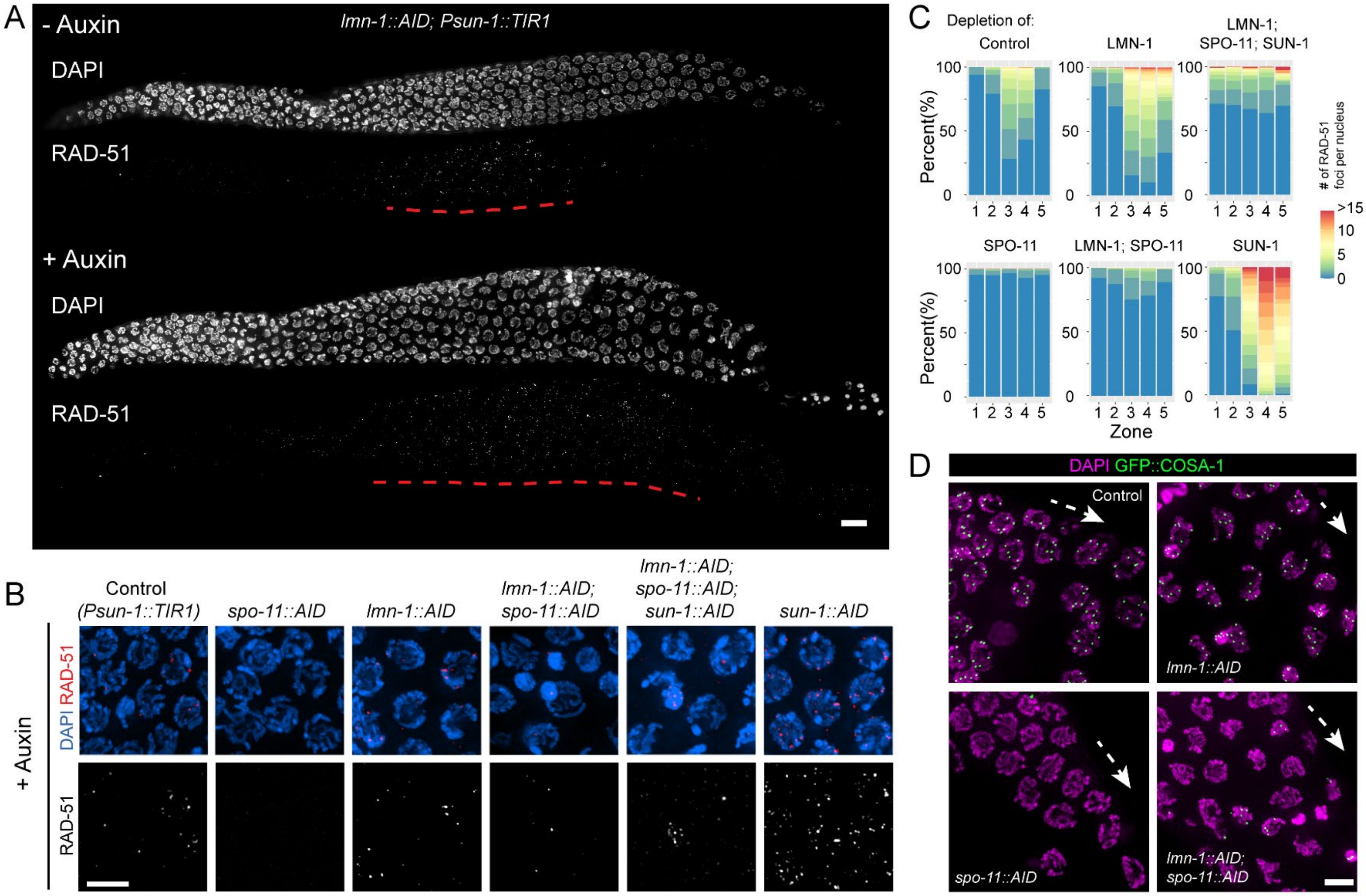
A
- Auxin
lmn-1::AID; Psun-1::TIR1
DAPI
RAD-51
+ Auxin
DAPI
RAD-51
B
Control (Psun-1::TIR1)
spo-11::AID
lmn-1::AID
lmn-1::AID; spo-11::AID
lmn-1::AID; spo-11::AID; sun-1::AID
sun-1::AID
+ Auxin
DAPI RAD-51
RAD-51
C
Depletion of:
Control
LMN-1
LMN-1; SPO-11; SUN-1
SPO-11
LMN-1; SPO-11
SUN-1
Percent(%)
Zone
# of RAD-51 foci per nucleus
>15
10
5
0
D
DAPI GFP::COSA-1
Control
lmn-1::AID
spo-11::AID
lmn-1::AID; spo-11::AID

Fig. S4. (Related to Figs. 1 and 3) LMN-1 depletion causes SPO-11 independent DNA damage. (**A**) LMN-1 depletion causes persistent DNA damage marked by RAD-51 foci. Scale bar, 10 µm. (**B**) RAD-51 staining in mid-pachytene nuclei from control and hermaphrodites depleted of SPO-11, LMN-1, LMN-1 and SPO-11 both, or LMN-1 and SPO-11 and SUN-1 simultaneously. Control hermaphrodite has TIR1 but no AID-tagged genes. Animals of indicated genotypes (all homozygous for $P_{sun\text{-}1}$*::TIR1*) were exposed to auxin for 24 hours from the L4 stage to young adulthood prior to dissection. Scale bar, 5 µm. (**C**) Quantification of RAD-51 foci per nucleus as a function of meiotic progression. Gonads were divided into five zones of equal length spanning the premeiotic region to early diplotene (as in **Fig. S6C**). Control, N = 964 nuclei (4 animals); SPO-11 depletion, N = 1480 nuclei (4 animals); LMN-1 depletion, N = 1781 nuclei (6 animals); LMN-1, SPO-11 double depletion, N = 1329 nuclei (5 animals); LMN-1, SPO-11, SUN-1 triple depletion, N = 844 nuclei (5 animals); SUN-1 depletion, N = 1201 nuclei (4 animals). Pairwise comparisons for proportions were performed to compute the *p*-values (adjusted by the Benjamini-Hochberg method, see **Data S1**). Experimental conditions were the same as in (**B**). (**D**) Designated crossover sites marked by GFP::COSA-1 foci in late prophase nuclei. Following LMN-1 depletion, nuclei still showed six designated crossover sites, whereas GFP::COSA-1 foci were absent from apoptotic nuclei. Animals of indicated genotypes (all homozygous for $P_{sun\text{-}1}$*::TIR1*) were exposed to auxin for 24 hours from the L4 stage to young adulthood prior to dissection. Dashed arrows indicate meiotic progression. Scale bar, 5 µm.

Fig. S5.

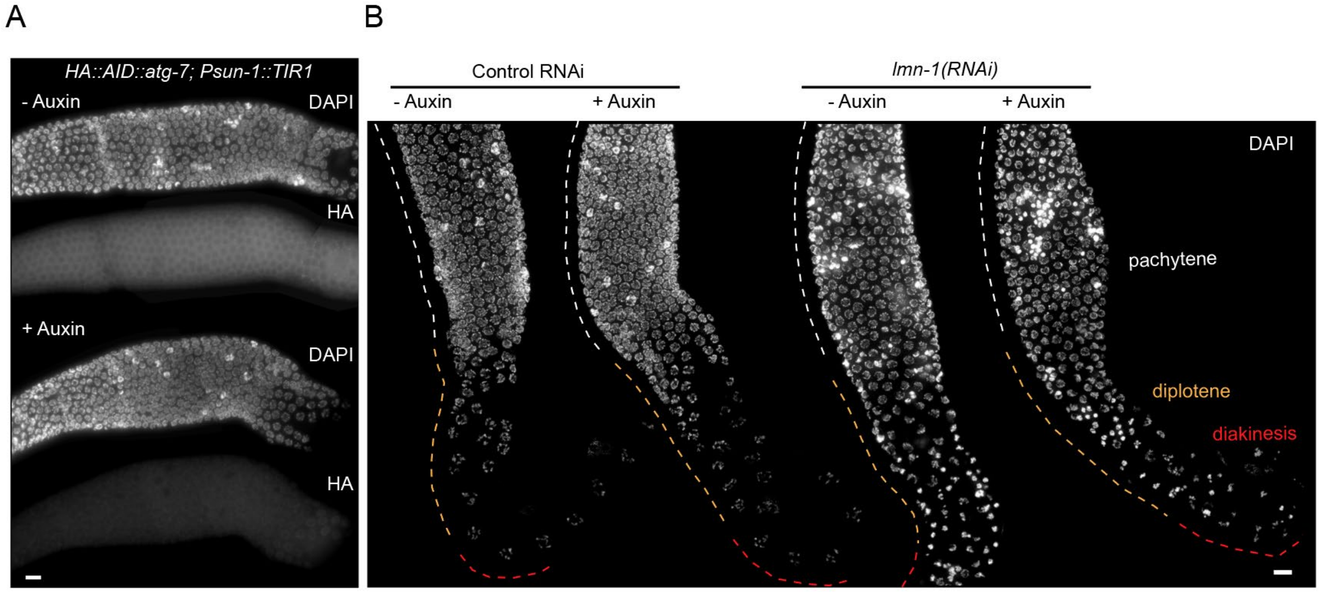

(Non-deconvolved max. Z projection)

Fig. S5. ATG-7 depletion does not rescue nuclear collapse. (A) Raw (non-deconvolved) maximum-intensity Z-projection showing the cytoplasmic localization of ATG-7 in the germline and the effectiveness of depletion by 12hr auxin treatment. Gray-scale images were scaled identically. Scale bar, 10 μm. (B) Nuclear morphology at later stages of meiotic prophase. Colored dashed lines mark meiotic stages based on the anatomical positions of gonads from control animals. Scale bar, 10 μm.

Fig. S6.

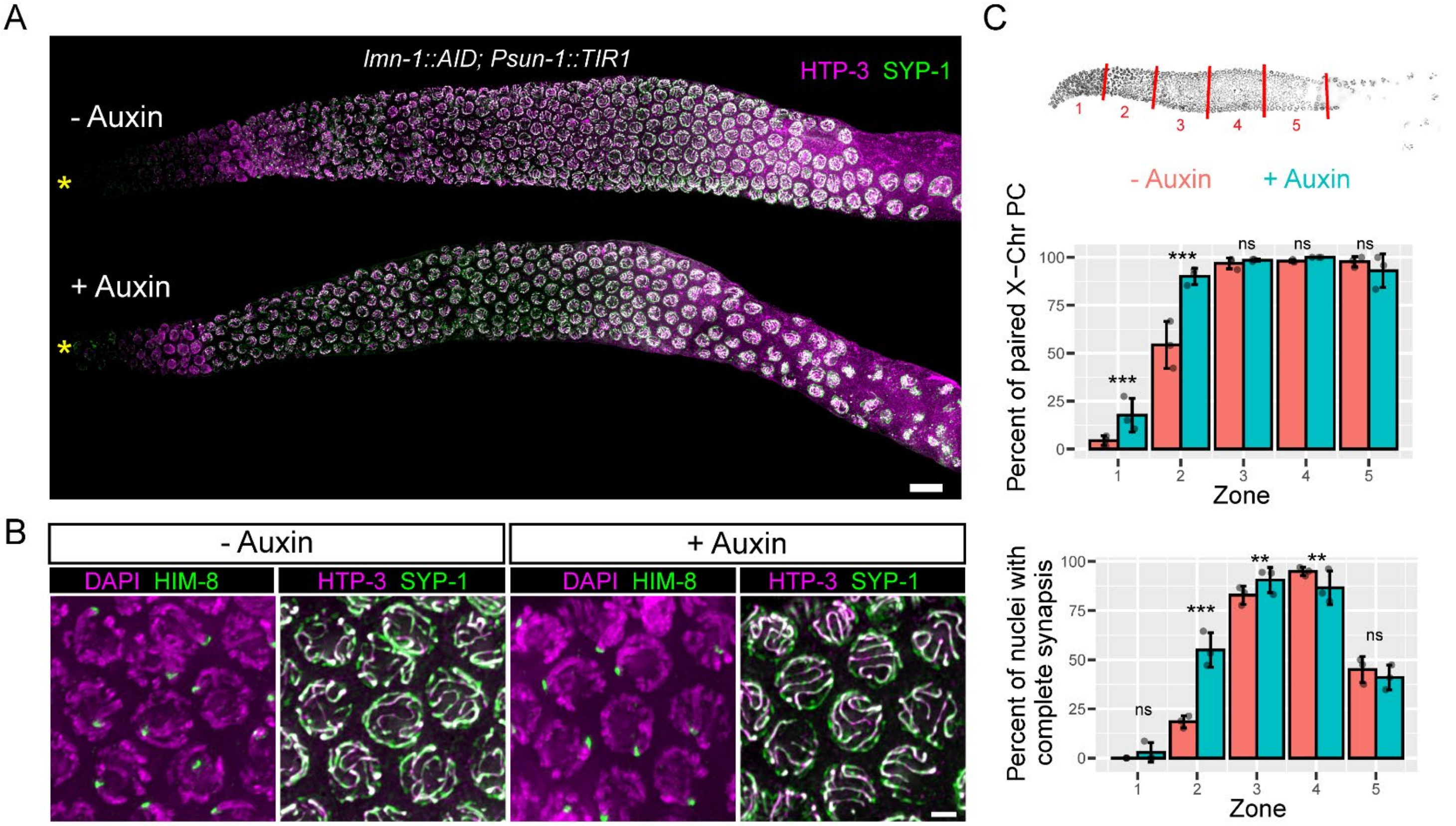

A
lmn-1::AID; Psun-1::TIR1
HTP-3 SYP-1
- Auxin
+ Auxin
B
- Auxin
+ Auxin
DAPI HIM-8
HTP-3 SYP-1
DAPI HIM-8
HTP-3 SYP-1
C
1
2
3
4
5
- Auxin
+ Auxin
Percent of paired X−Chr PC
Zone
Percent of nuclei with complete synapsis
Zone
***
ns
**

Fig. S6. (Related to Fig. 2) Chromosome pairing and synapsis occur normally following LMN-1 depletion. (**A**) Dynamics of synapsis, based on immunostaining of SYP-1 (synaptonemal complex) and HTP-3 (chromosome axes). Yellow asterisks indicate the distal end of gonads. Scale bar, 10 µm. (**B**) Normal pairing of X chromosomes is revealed by immunofluorescence of HIM-8, which localizes to the X-chromosome pairing centers. Scale bar, 2 µm. (**C**) Quantification of homolog pairing and synapsis. Diagram of distal gonad divided into five zones of equal length. Three gonads were measured per condition. Mean ± SD are plotted. Two-sided two-proportions *z*-test was used for computing the *p*-values. ***, $p < 0.001$; **, $p < 0.01$; ns, $p \geq 0.05$ (see **Data S1**).

Fig. S7.

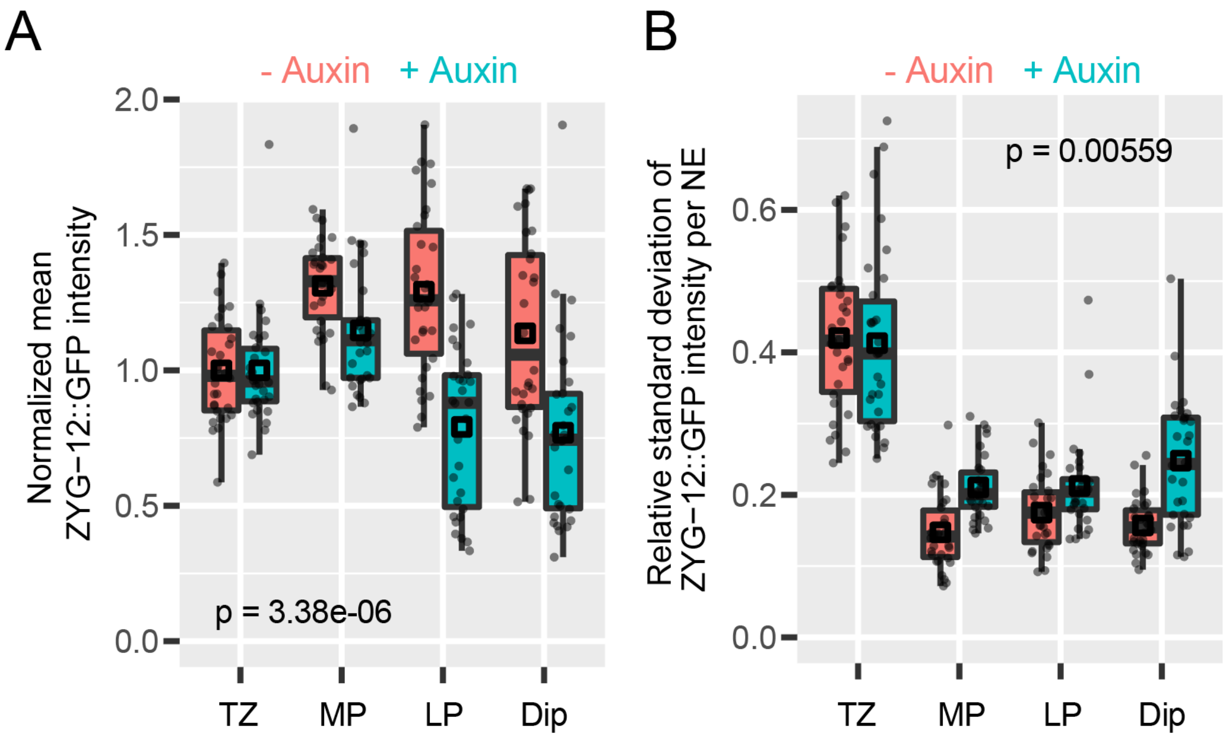
A
- Auxin
+ Auxin
Normalized mean
ZYG−12::GFP intensity
2.0
1.5
1.0
0.5
0.0
p = 3.38e-06
TZ
MP
LP
Dip
B
- Auxin
+ Auxin
Relative standard deviation of
ZYG−12::GFP intensity per NE
0.6
0.4
0.2
0.0
p = 0.00559
TZ
MP
LP
Dip

Fig. S7. (Related to Fig. 2) Prolonged ZYG-12::GFP clustering following LMN-1 depletion. (**A**) Mean intensities of ZYG-12::GFP at the nuclear envelope per nucleus, normalized against intensity levels at the transition zone. Medians (black crossbars) and means (black boxes) are shown. Fluorescence intensity surrounding each nucleus was manually segmented and quantified from additive projection images after background subtraction. 30 nuclei per stage were measured per condition (without or with auxin). TZ, transition zone; MP, mid-pachytene; LP, late pachytene; Dip, diplotene. Two-way ANOVA was used to calculate the p-value. (**B**) Clustering of ZYG-12::GFP, defined as the relative standard deviation (ratio of the standard deviation to the mean value) of fluorescence intensity at the NE in each nucleus. Medians (black crossbars) and means (black boxes) are shown. Fluorescence intensity was measured in the same way as in (**A**). 30 nuclei per stage were measured per condition. TZ, transition zone; MP, mid-pachytene; LP, late pachytene; Dip, diplotene. *p*-value was calculated by two-way ANOVA.

Fig. S8.

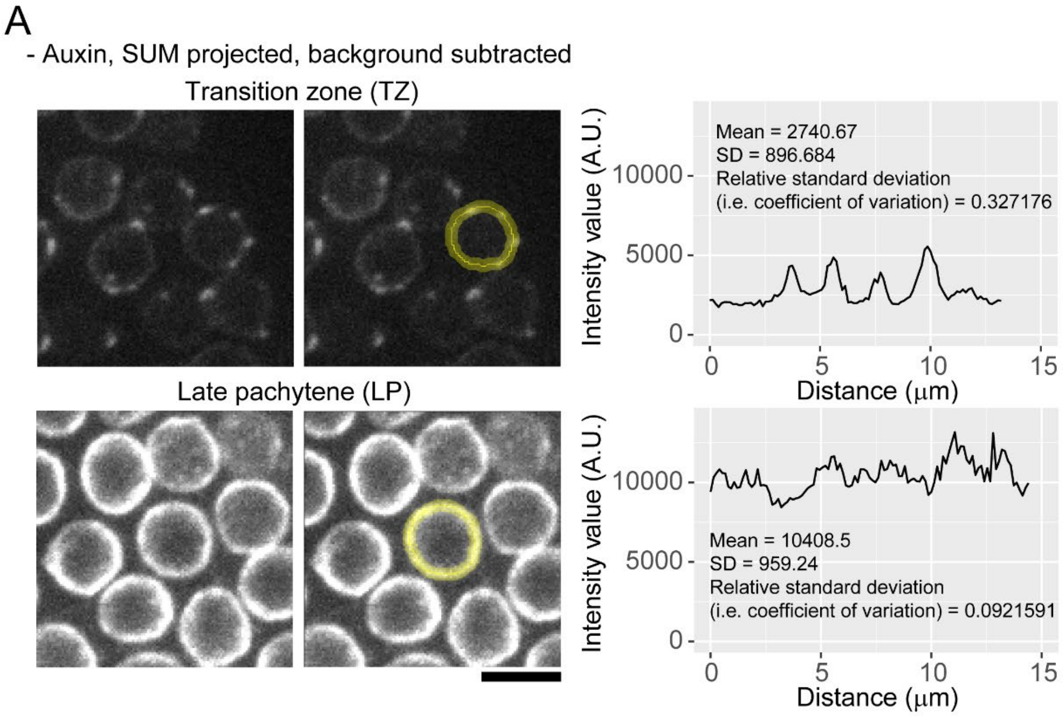
A
- Auxin, SUM projected, background subtracted
Transition zone (TZ)
Mean = 2740.67
SD = 896.684
Relative standard deviation
(i.e. coefficient of variation) = 0.327176
Intensity value (A.U.)
Distance (μm)
Late pachytene (LP)
Mean = 10408.5
SD = 959.24
Relative standard deviation
(i.e. coefficient of variation) = 0.0921591
Intensity value (A.U.)
Distance (μm)

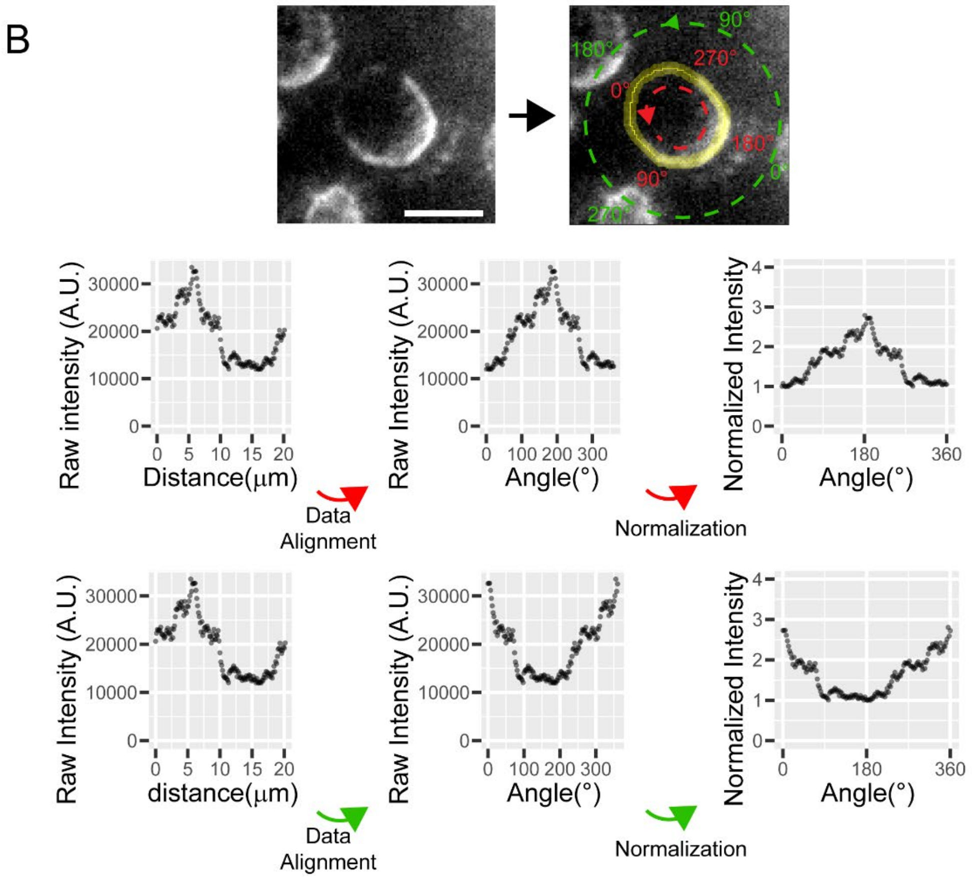
B
90°
180°
270°
0°
Raw intensity (A.U.)
Distance(μm)
Data Alignment
Raw Intensity (A.U.)
Angle(°)
Normalization
Normalized Intensity
Angle(°)
Raw Intensity (A.U.)
distance(μm)
Data Alignment
Raw Intensity (A.U.)
Angle(°)
Normalization
Normalized Intensity
Angle(°)

Fig. S8. (Related to Figs. 2 and 3) Quantifying LINC complex distribution at the NE. (**A**) Quantifying SUN-1 distribution at the NE. Representative line-scan profiles of SUN-1::mRuby fluorescence intensity at the circumference of individual nuclei. Grayscale images are additive projections and are scaled using the same look up table (LUT). Scale bar, 5 µm. (**B**) Quantifying asymmetric ZYG-12 distribution at the NE. Top panel shows grayscale images of additive projections with background subtraction showing polarized ZYG-12::GFP distribution at the NE in a LMN-1 depleted diplotene nucleus. The right panel shows line scan profile along the circumference of the nucleus and approximate locations of angles mapped subsequently during data alignment. Scale bar, 5 µm. Bottom panels show data alignment and normalization. Raw intensity measurement from line-scan was aligned and mapped such that 180° corresponds to the coordinate along the NE's circumference with the maximum-intensity, and the intensity at 0° was normalized to one (red); or in cases where one nucleus has multiple ZYG-12 intensity peaks on the NE, 180° corresponds to the coordinate along the NE's circumference with the minimum-intensity, and the intensity at 180° was normalized to one (green).

Fig. S9.

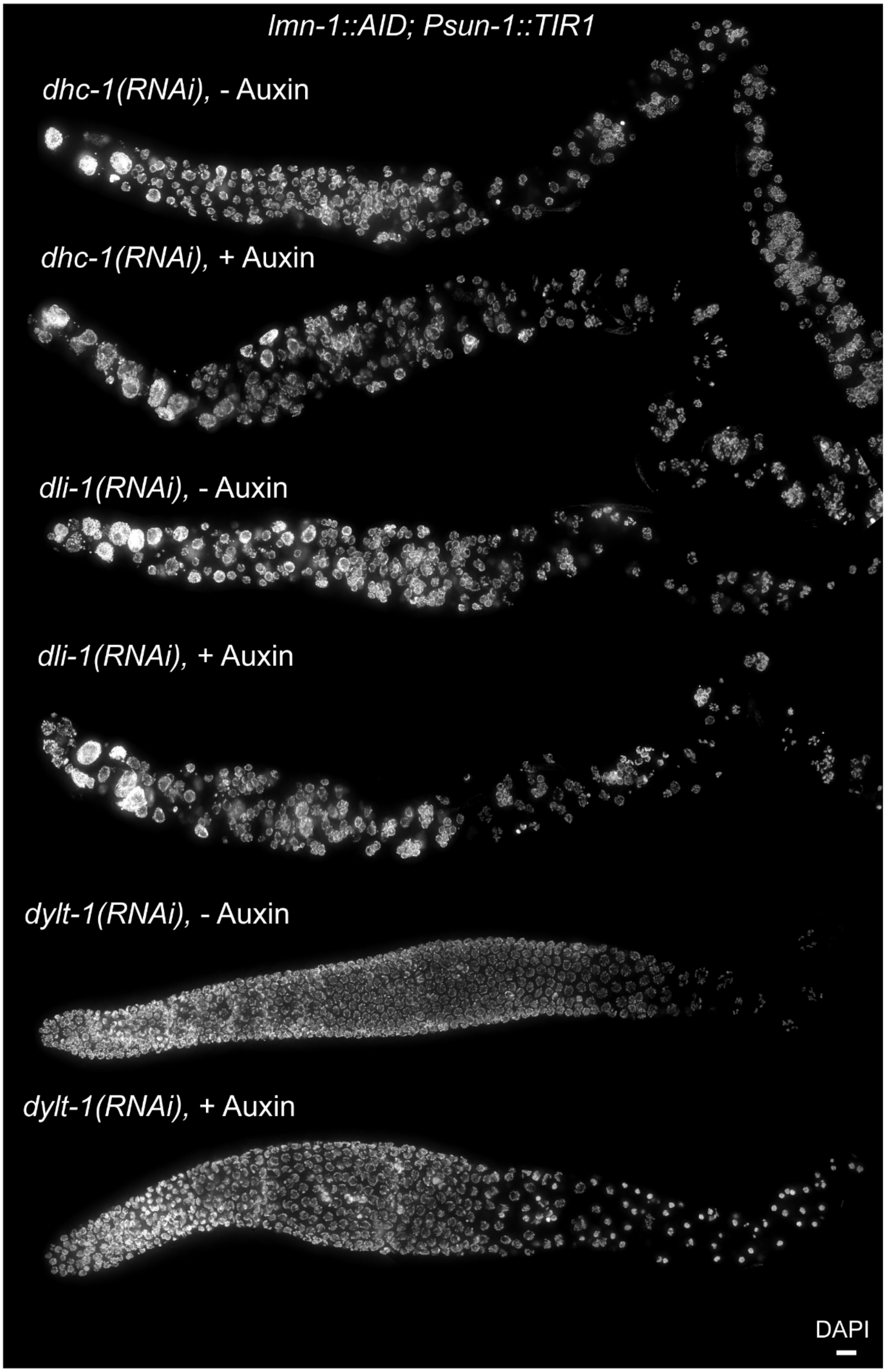

lmn-1::AID; Psun-1::TIR1
dhc-1(RNAi), - Auxin
dhc-1(RNAi), + Auxin
dli-1(RNAi), - Auxin
dli-1(RNAi), + Auxin
dylt-1(RNAi), - Auxin
dylt-1(RNAi), + Auxin
DAPI

Fig. S9. (Related to Fig. 3) Co-depletion of DHC-1 or DLI-1, but not DYLT-1, also rescues nuclear collapse despite marked nuclear mispositioning. Nuclear morphology upon depleting DHC-1, DLI-1 or DYLT-1 in *lmn-1::AID::V5* worms ±auxin treatment. Mitotic defects are seen in the proliferative region, and meiotic nuclei are mispositioned throughout the gonad following depletion of DHC-1 or DLI-1. Scale bar, 10 µm.

Fig. S10.

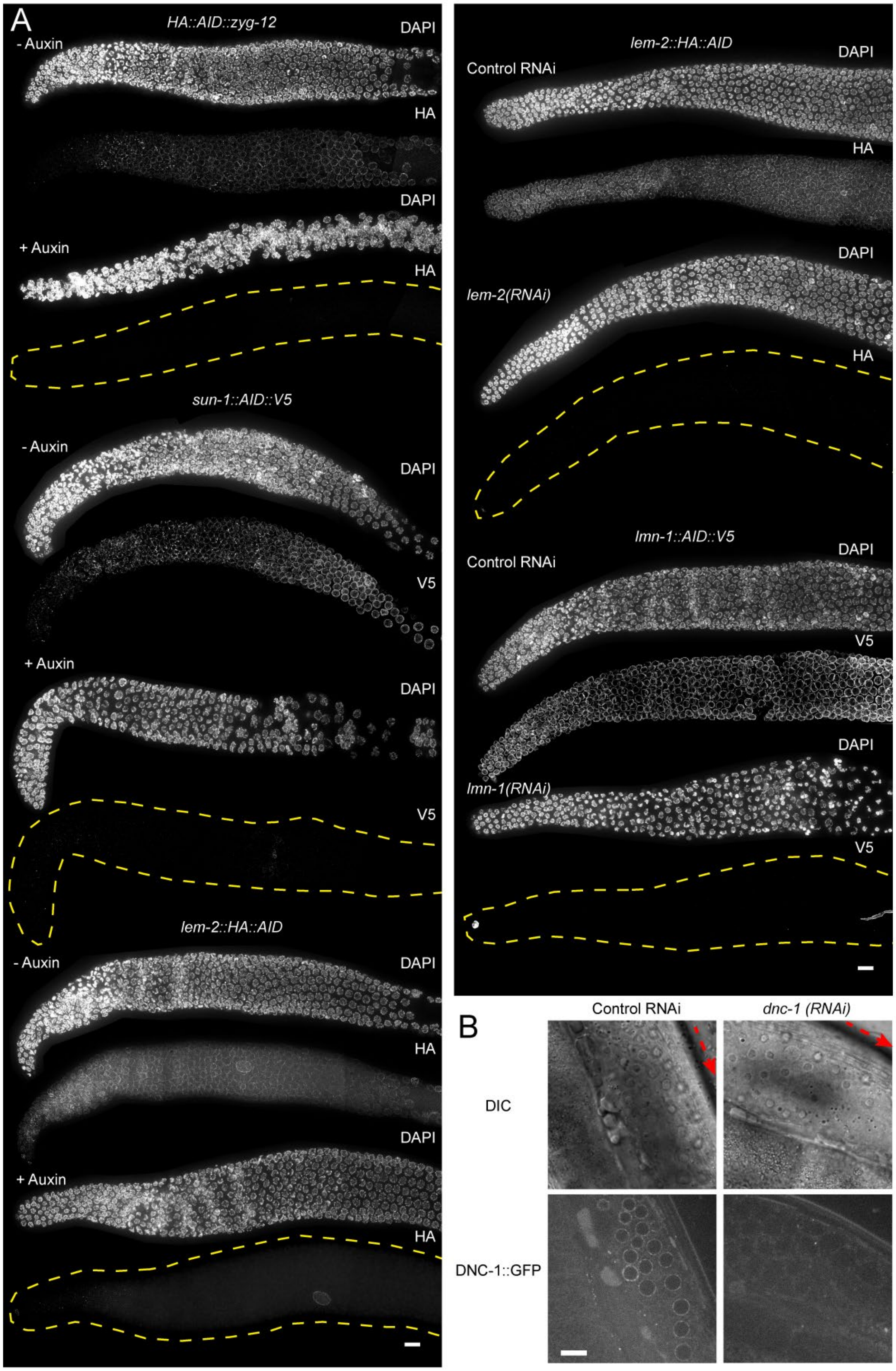
A
HA::AID::zyg-12
- Auxin
DAPI
HA
DAPI
+ Auxin
HA
sun-1::AID::V5
- Auxin
DAPI
V5
+ Auxin
DAPI
V5
lem-2::HA::AID
- Auxin
DAPI
HA
DAPI
+ Auxin
HA
lem-2::HA::AID
Control RNAi
DAPI
HA
DAPI
lem-2(RNAi)
HA
lmn-1::AID::V5
Control RNAi
DAPI
V5
DAPI
lmn-1(RNAi)
V5
B
Control RNAi
dnc-1 (RNAi)
DIC
DNC-1::GFP

Fig. S10. (Related to Figs. 3 and 4) Efficacy of auxin-induced degradation or RNAi. (**A**) Nuclear morphology in hermaphrodites depleted of ZYG-12, SUN-1 or LEM-2 with auxin-induced degradation, or depleted of LEM-2 or LMN-1 using RNAi. The duration of auxin treatment was at least 8 hours and the duration of feeding RNAi was 48hr. Scale bars, 10 µm. All worms were homozygous for P_{sun-1}*::TIR1* or P_{gld-1}*::TIR1*. ZYG-12 depletion results in mispositioning of meiotic nuclei. (**B**) DNC-1 at NE can be effectively depleted using RNAi. Live imaging stills of DIC and DNC-1::GFP in the late pachytene region of the germline in worms fed with Control RNAi or *dnc-1(RNAi)*. Arrows indicate meiotic progression. Scale bar, 10 µm.

Fig. S11.

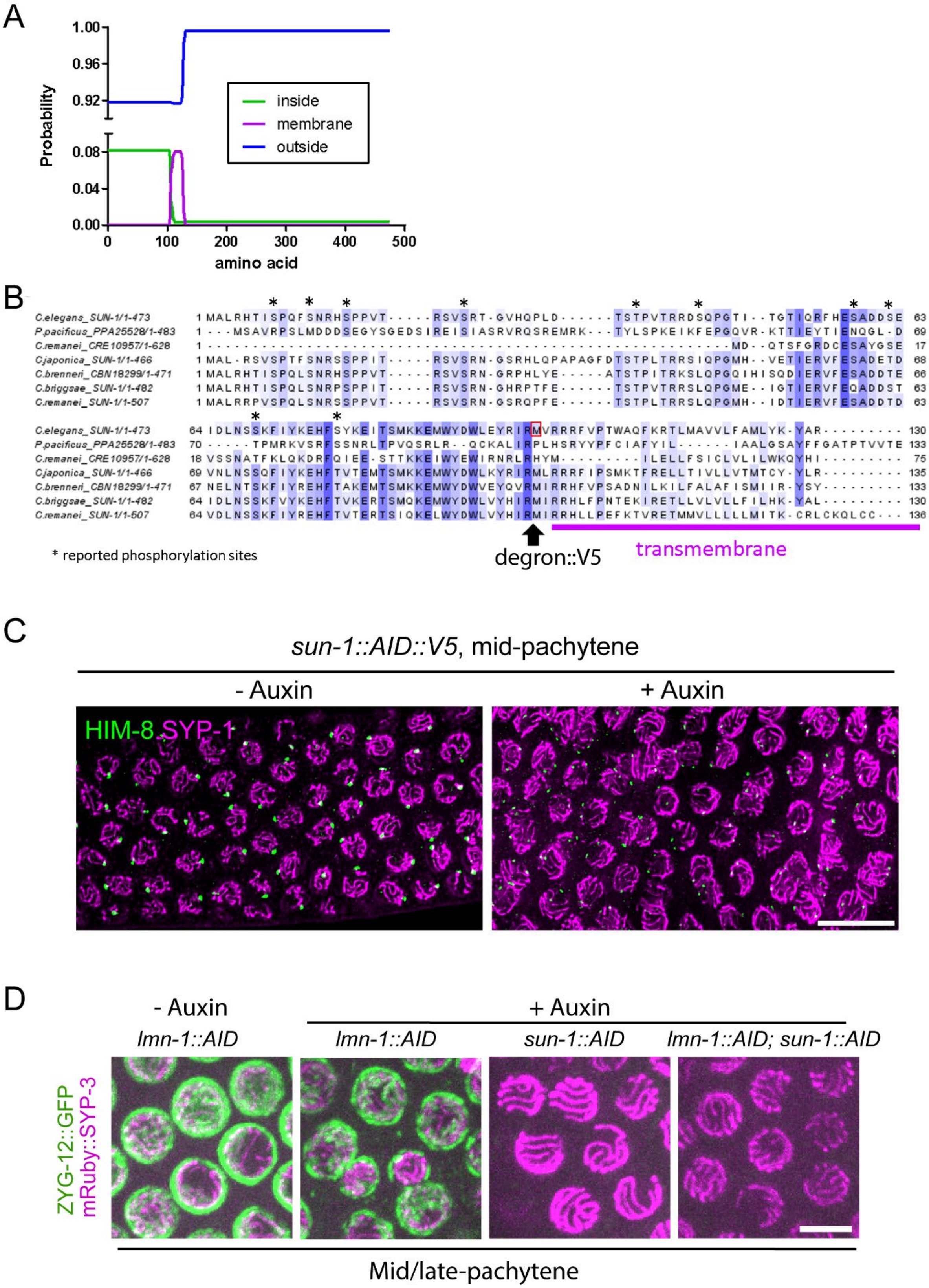
A
Probability
1.00
0.96
0.92
0.08
0.04
0.00
0
100
200
300
400
500
amino acid
inside
membrane
outside
B
C.elegans_SUN-1/1-473
P.pacificus_PPA25528/1-483
C.remanei_CRE10957/1-628
C.japonica_SUN-1/1-466
C.brenneri_CBN18299/1-471
C.briggsae_SUN-1/1-482
C.remanei_SUN-1/1-507
* reported phosphorylation sites
degron::V5
transmembrane
C
sun-1::AID::V5, mid-pachytene
- Auxin
+ Auxin
HIM-8 SYP-1
D
- Auxin
+ Auxin
lmn-1::AID
lmn-1::AID
sun-1::AID
lmn-1::AID; sun-1::AID
ZYG-12::GFP
mRuby::SYP-3
Mid/late-pachytene

Fig. S11. (Related to Fig. 3) CRISPR tagging and auxin-inducible degradation of SUN-1 in *C. elegans* germline. (**A**) Prediction of transmembrane region in *C. elegans* SUN-1 using TMHMM (http://www.cbs.dtu.dk/services/TMHMM/). Probability of amino acids inside the INM (green), outside the ONM (blue) and in the transmembrane region (magenta) is plotted. (**B**) Multiple sequence alignments of nematode SUN-1 proteins were generated using Clustal Omega and visualized using Jalview, showing position of degron/V5 insertion in *C. elegans* SUN-1. (**C**) X-chromosome pairing (HIM-8) and SC assembly (SYP-1) in *sun-1::AID::V5* worms without or with auxin treatment. All worms were homozygous for $P_{sun\text{-}1}$*::TIR1*. Scale bar, 10 μm. (**D**) SUN-1 is required for ZYG-12 localization at the NE of meiotic cells. Composite images showing live meiotic nuclei from mid/late pachytene in worms of indicated genotypes or treatments. All worms were homozygous for $P_{sun\text{-}1}$*::TIR1* or $P_{gld\text{-}1}$*::TIR1*. ZYG-12::GFP in green and mRuby::SYP-3 in magenta. All images are maximum-intensity projections and scaled identically. Scale bar, 5 μm.

Fig. S12.

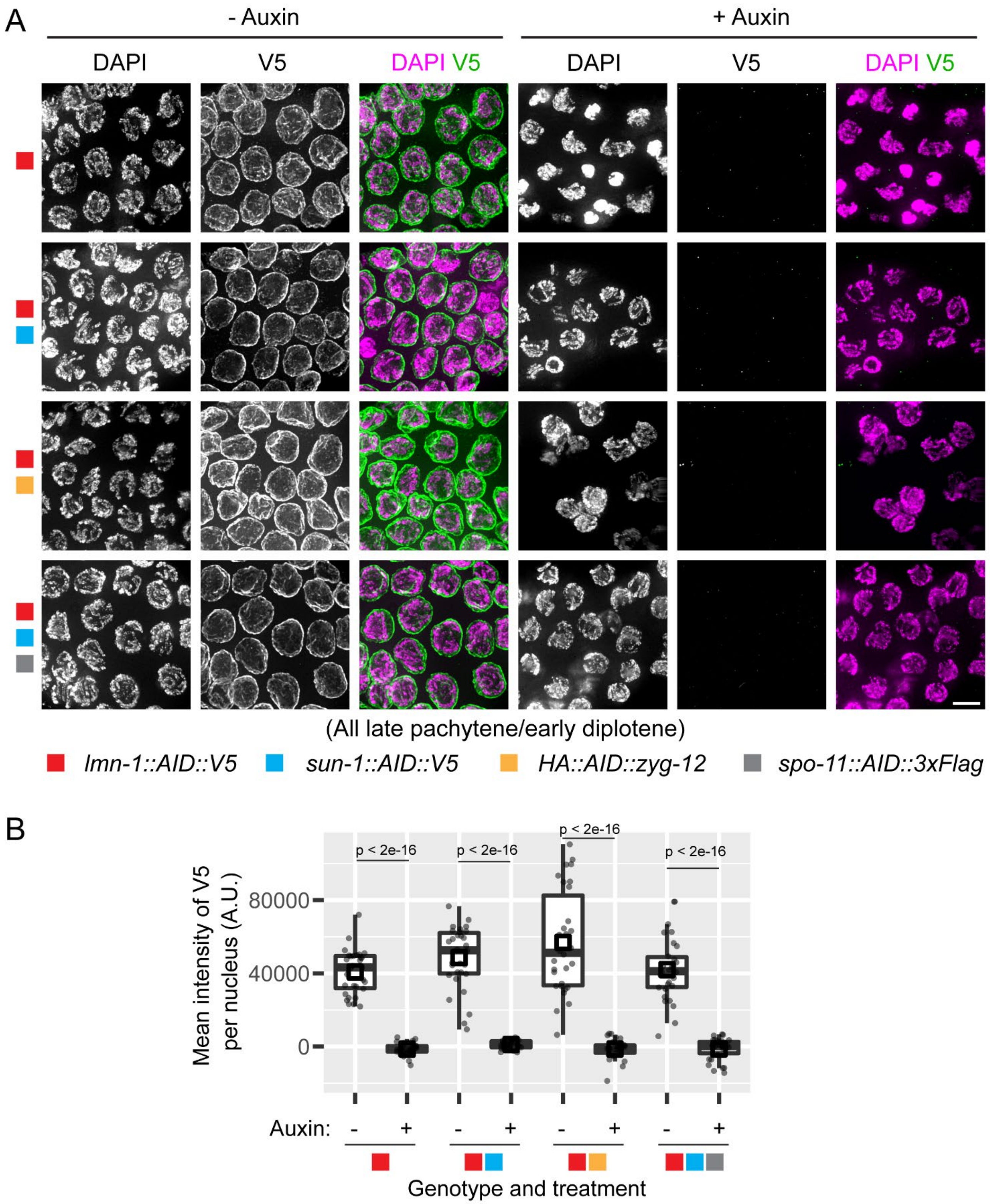
A
- Auxin
+ Auxin
DAPI
V5
DAPI V5
(All late pachytene/early diplotene)
lmn-1::AID::V5
sun-1::AID::V5
HA::AID::zyg-12
spo-11::AID::3xFlag
B
Mean intensity of V5 per nucleus (A.U.)
80000
40000
0
p < 2e-16
Auxin:
-
+
Genotype and treatment

Fig. S12. (Related to Fig. 3) LMN-1 depletion is equally efficient upon auxin-induced co-degradation. (**A**) Four strains carrying *lmn-1::AID::V5* alone or in combination with other AID-tagged genes were immunostained for V5 after the same 12hr treatment from young adult (-/+ Auxin). All strains have P_{sun-1} or P_{gld-1} driven TIR1::mRuby. All images are maximum-intensity projections and scaled identically between -/+ Auxin. Scale bar, 5 µm. (**B**) Quantification of mean intensity of V5 staining per nucleus. Fluorescence intensity was measured from additive projection images after background subtraction. 30 nuclei in late pachytene/early diplotene were measured per condition. Medians (black crossbars) and means (black boxes) are shown. *p*-values were calculated using one-way ANOVA and *post hoc* pairwise *t*-tests (two-sided; adjusted by the Benjamini-Hochberg method). $p > 0.05$ between all + Auxin groups. A.U., arbitrary unit.

Fig. S13.

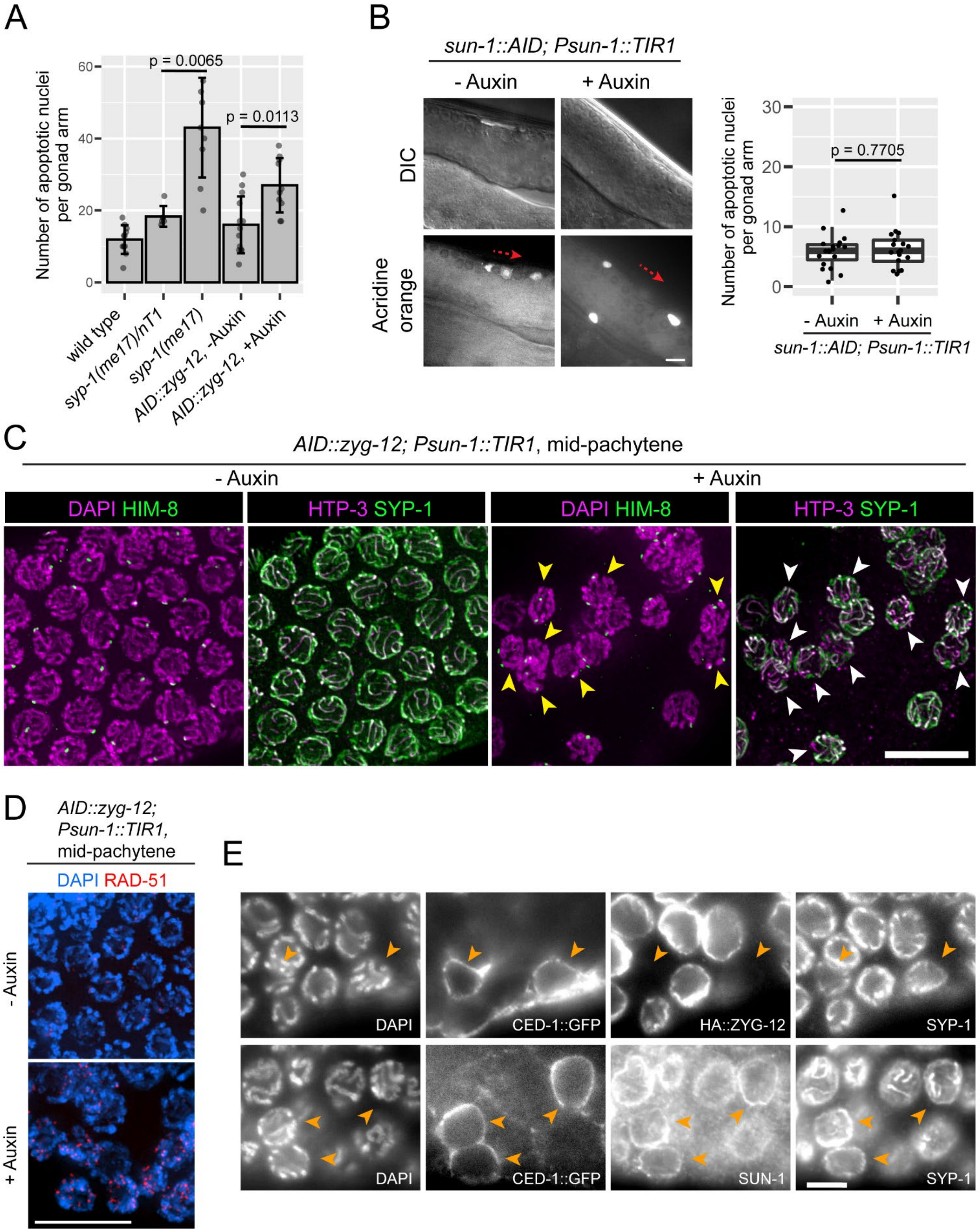
A
Number of apoptotic nuclei per gonad arm
60
40
20
0
p = 0.0065
p = 0.0113
wild type
syp-1(me17)/nT1
syp-1(me17)
AID::zyg-12, -Auxin
AID::zyg-12, +Auxin
B
sun-1::AID; Psun-1::TIR1
- Auxin
+ Auxin
DIC
Acridine orange
Number of apoptotic nuclei per gonad arm
30
20
10
0
p = 0.7705
- Auxin
+ Auxin
sun-1::AID; Psun-1::TIR1
C
AID::zyg-12; Psun-1::TIR1, mid-pachytene
- Auxin
+ Auxin
DAPI HIM-8
HTP-3 SYP-1
DAPI HIM-8
HTP-3 SYP-1
D
AID::zyg-12; Psun-1::TIR1, mid-pachytene
DAPI RAD-51
- Auxin
+ Auxin
E
DAPI
CED-1::GFP
HA::ZYG-12
SYP-1
DAPI
CED-1::GFP
SUN-1
SYP-1

Fig. S13. (Related to Fig. 3) SUN-1, but not ZYG-12, is required for damage-induced apoptosis during meiosis. (**A**) Germline apoptosis is increased upon depleting ZYG-12. Apoptosis was quantified using CED-1::GFP. *syp-1(me17)/nT1* heterozygous and *syp-1(me17)* homozygous animals were used as controls. Mean ± SD are plotted. Pairwise Mann-Whitney test was used for computing the *p*-values. (**B**) Germline apoptosis does not change upon SUN-1 depletion. Red dashed arrows indicate the direction of meiotic progression. Scale bar, 10 µm. Unpaired two-sample two-sided Mann-Whitney test was used for computing the *p*-value. (**C**) X-chromosome pairing (HIM-8) and SC assembly (SYP-1) in *HA::AID::zyg-12* worms without or with auxin treatment. Yellow arrow heads indicate nuclei with unpaired HIM-8 foci, white arrow heads indicate nuclei with incomplete synapsis (HTP-3 staining devoid of SYP-1). Scale bar, 10 µm. **(D)** RAD-51 staining in mid-pachytene nuclei in *HA::AID::zyg-12* worms without or with auxin treatment. Scale bar, 10 µm. (**E**) Single Z-section of non-deconvolved immunofluorescence images showing CED-1::GFP positive meiotic nuclei stain positive for SUN-1 but negative for ZYG-12 (orange arrow heads). One worm per row is shown. Scale bar, 5 µm.

Fig. S14.

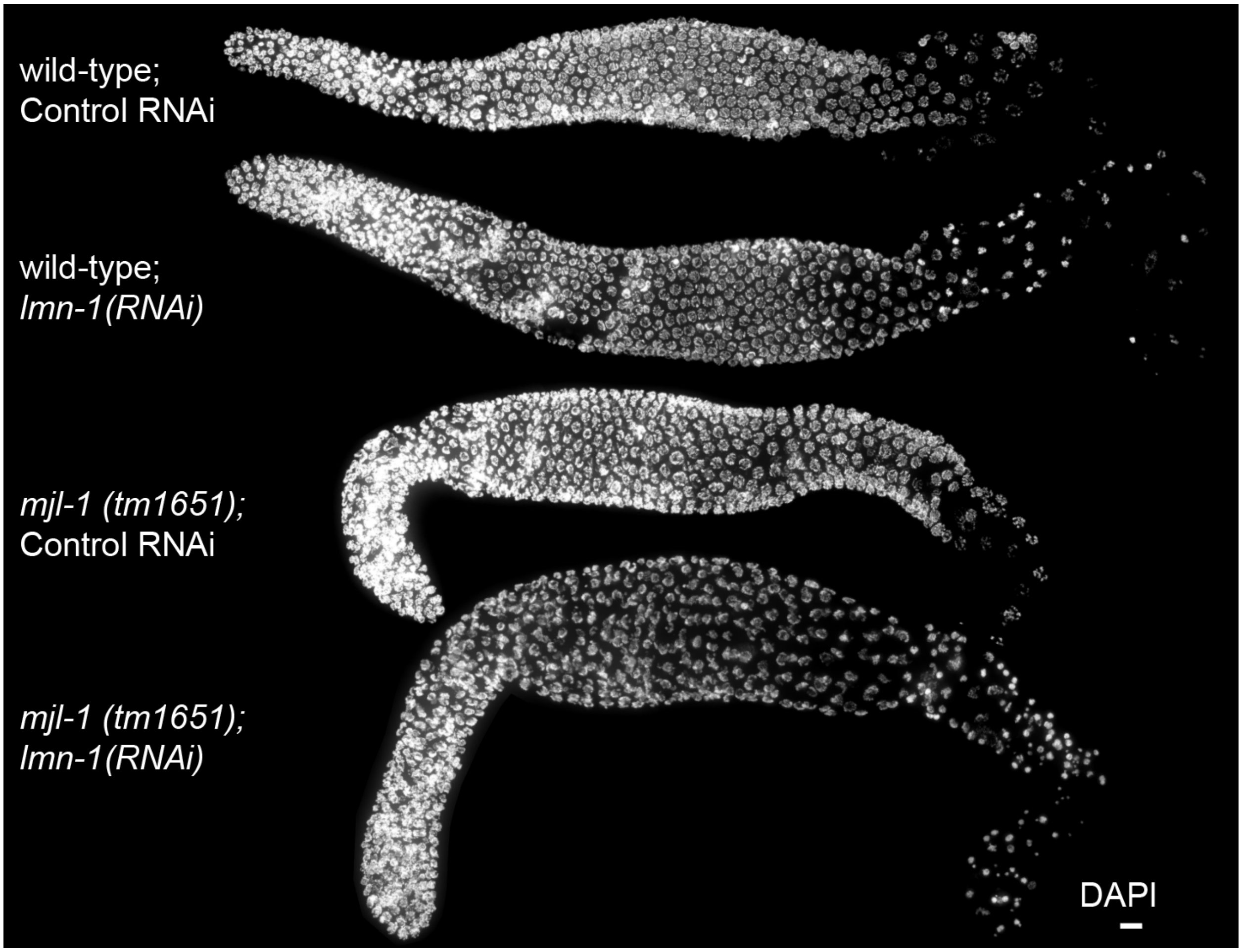
wild-type;
Control RNAi
wild-type;
lmn-1(RNAi)
mjl-1 (tm1651);
Control RNAi
mjl-1 (tm1651);
lmn-1(RNAi)
DAPI

Fig. S14. (Related to Fig. 3) The absence of the meiotic NE protein MJL-1 does not rescue nuclear collapse. Nuclear morphology in wild-type (N2) or *mjl-1 null (tm1651)* worms following Control RNAi or *lmn-1(RNAi)*. Scale bar, 10 µm.

Fig. S15.

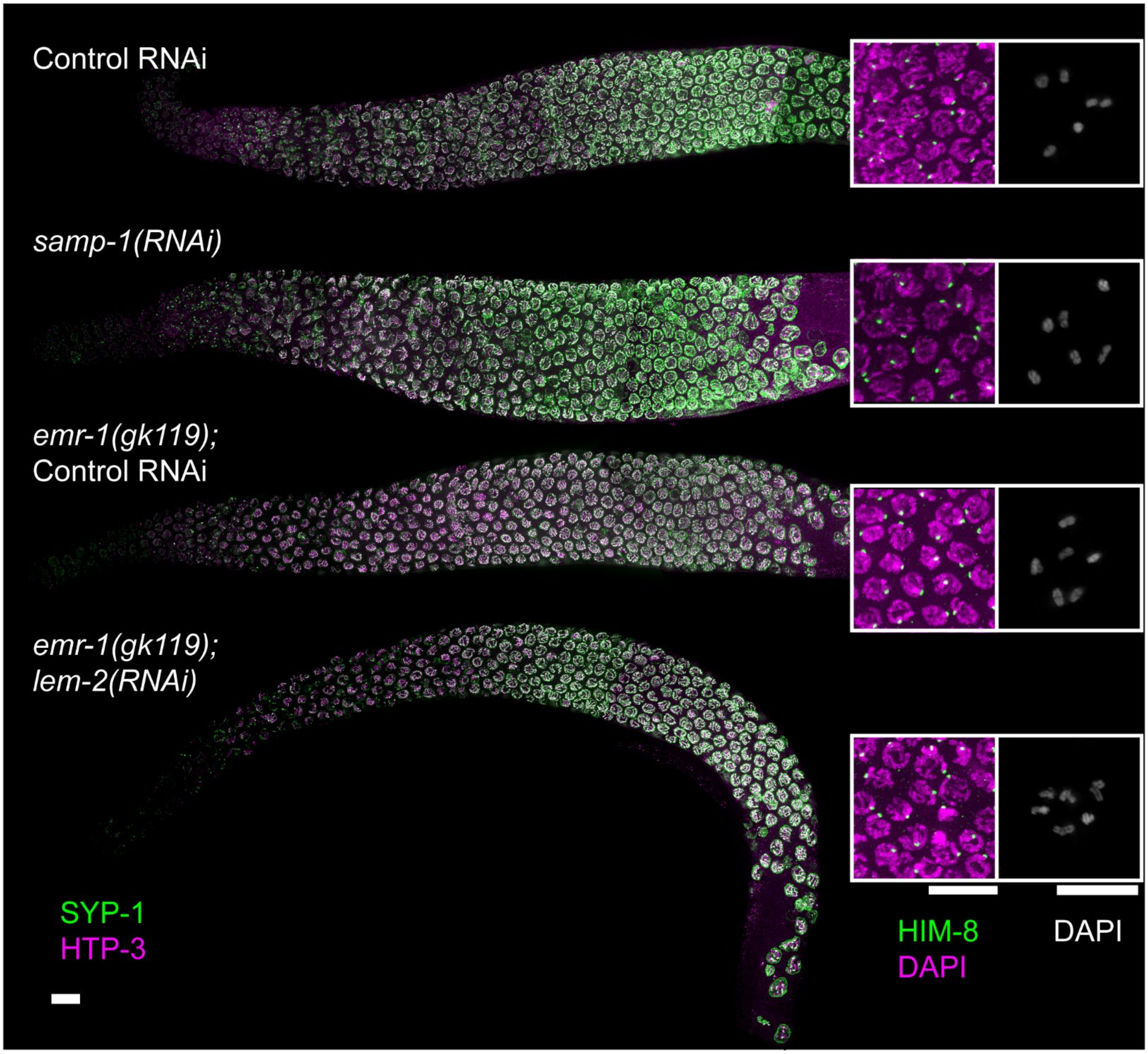

Control RNAi
samp-1(RNAi)
emr-1(gk119);
Control RNAi
emr-1(gk119);
lem-2(RNAi)
SYP-1
HTP-3
HIM-8
DAPI
DAPI

Fig. S15. (Related to Fig. 4) Pairing and synapsis upon depleting SAMP-1, EMR-1 or LEM-2. X-chromosome pairing (HIM-8) and SC assembly (SYP-1 and HTP-3) upon RNAi-mediated depletion of SAMP-1 or of LEM-2 in *emr-1(gk119)* mutants. Insets showing X-chromosome pairing in early pachytene and bivalents formation in diakinesis under each condition. Scale bars, 10 μm.

Fig. S16.

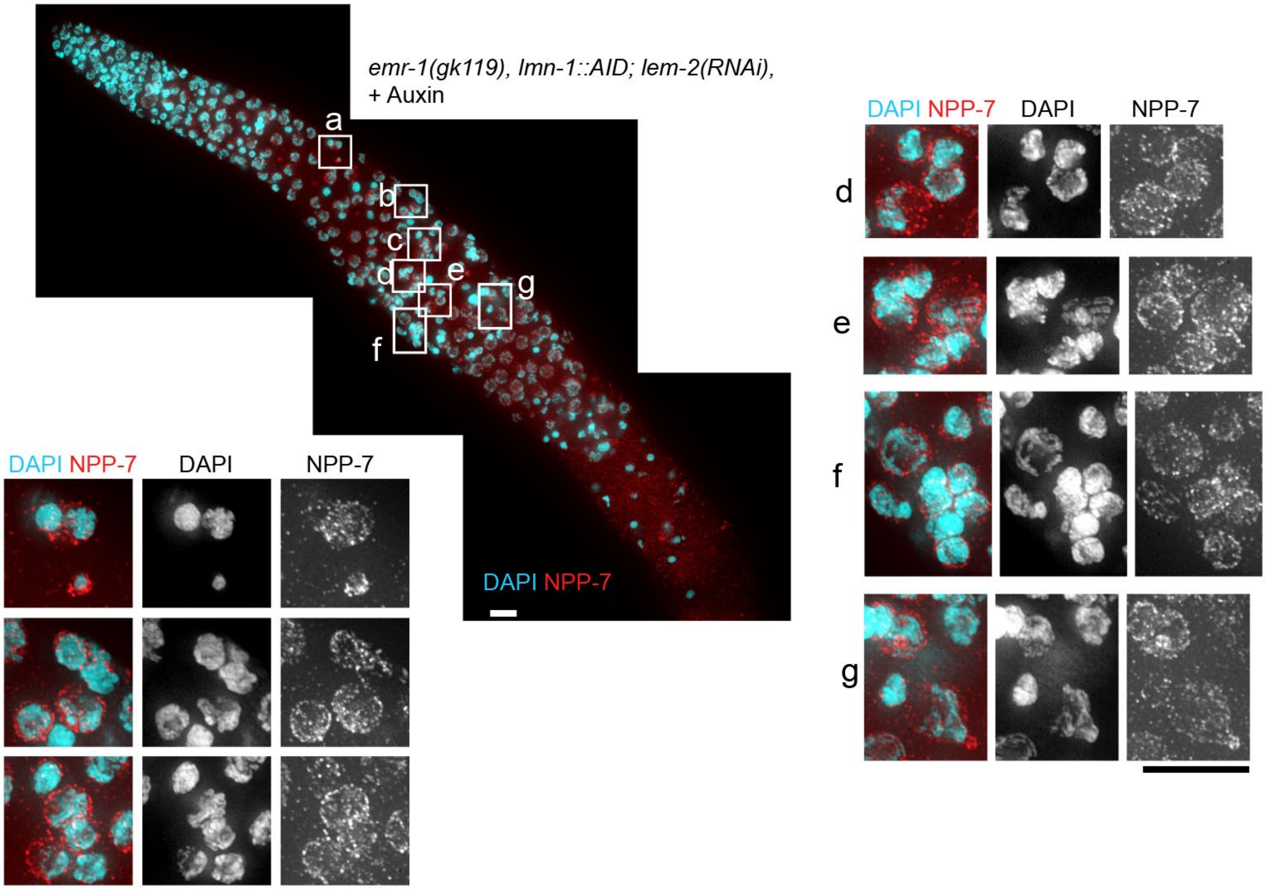
emr-1(gk119), lmn-1::AID; lem-2(RNAi),
+ Auxin
a
b
c
d
e
f
g
DAPI NPP-7
DAPI NPP-7
DAPI
NPP-7
a
b
c
DAPI NPP-7
DAPI
NPP-7
d
e
f
g

Fig. S16. (Related to Fig. 4) Immunostaining of nuclear pores in germlines depleted of EMR-1, LEM-2 and LMN-1. Composite images showing a representative gonad from an *emr-1(gk119), lmn-1::AID* animal with *lem-2(RNAi)* and Auxin treatment, DAPI in cyan and NPP-7 (marking nuclear pore complex) in red. Insets show that nuclei undergoing exacerbated collapse can still be surrounded by nuclear pore complexes marked by NPP-7. All scale bars, 10 μm.

Fig. S17.

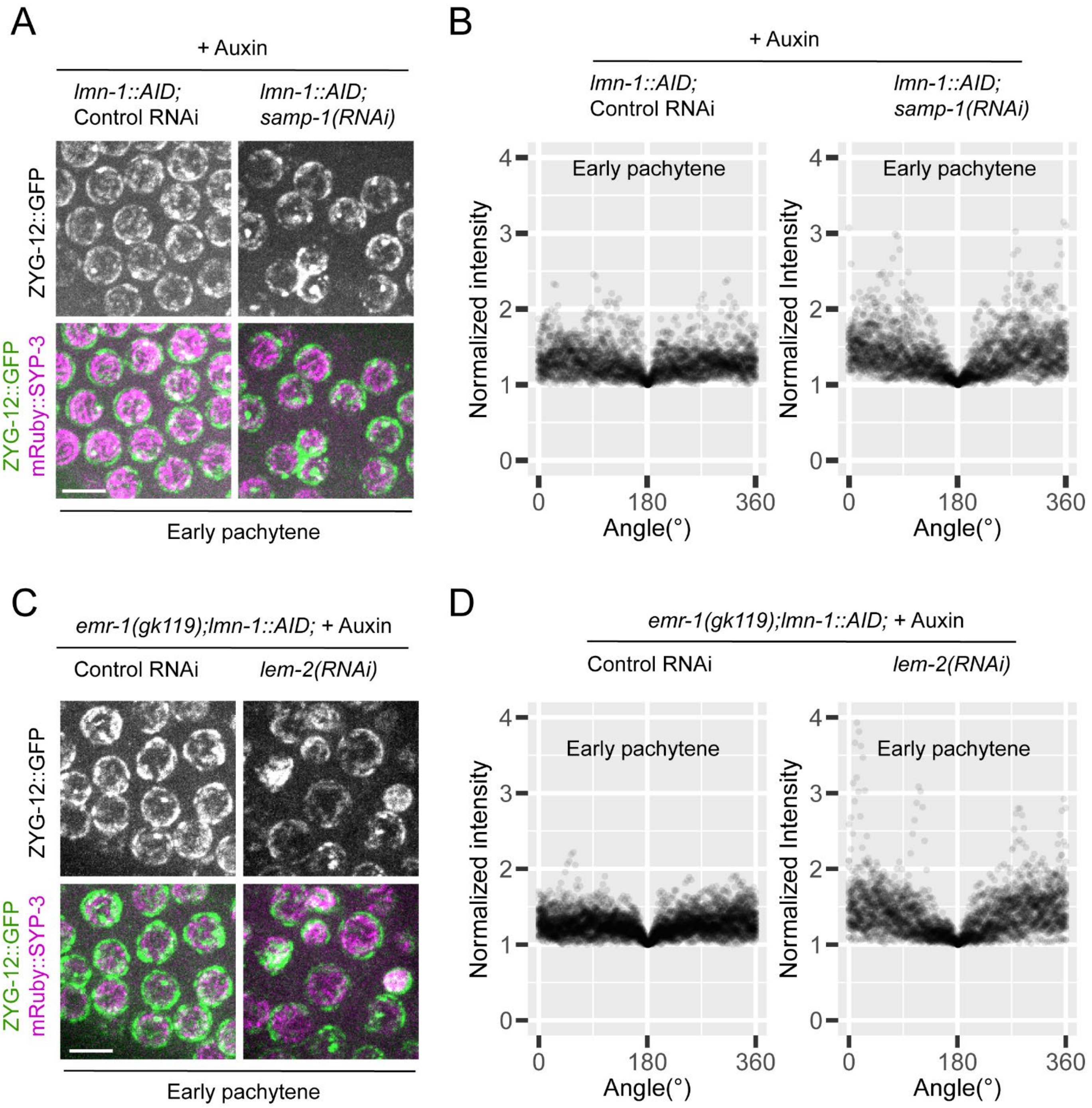

A
+ Auxin
lmn-1::AID; Control RNAi
lmn-1::AID; samp-1(RNAi)
ZYG-12::GFP
ZYG-12::GFP mRuby::SYP-3
Early pachytene
B
+ Auxin
lmn-1::AID; Control RNAi
lmn-1::AID; samp-1(RNAi)
Early pachytene
Normalized intensity
Normalized Intensity
Angle(°)
0 1 2 3 4
0 180 360
C
emr-1(gk119);lmn-1::AID; + Auxin
Control RNAi
lem-2(RNAi)
ZYG-12::GFP
ZYG-12::GFP mRuby::SYP-3
Early pachytene
D
emr-1(gk119);lmn-1::AID; + Auxin
Control RNAi
lem-2(RNAi)
Early pachytene
Normalized intensity
Normalized Intensity
Angle(°)

Fig. S17. (Related to Fig. 4) Asymmetric distribution of ZYG-12::GFP at early pachytene NE upon co-depleting LMN-1 and SAMP-1 or EMR-1/LEM-2. (**A** and **C**) Composite images showing mRuby::SYP-3 (magenta) and ZYG-12::GFP (green) at the NE of early pachytene nuclei of indicated genotypes or treatments. All images are maximum-intensity projections and scaled identically per experiment. Scale bar, 5 µm. (**B** and **D**) Quantification of ZYG-12::GFP fluorescence as a function of angle along the circumference of NE in early pachytene nuclei. Intensity measurement was performed as in **Fig. S8B**. Because of the multiple bright foci/patches of LINC complexes at the NE in each early pachytene nucleus, data had to be aligned and normalized differently than that in **Fig. 3B**: data were aligned and mapped so that 180° corresponds to the coordinate along the NE's circumference with the minimum-intensity of ZYG-12::GFP, which was normalized as one. In (**B**), N = 32 early pachytene nuclei pooled from four animals were measured for each condition. $p < 2.2\text{e-}16$ between the control RNAi and *samp-1(RNAi)* with 2-way ANOVA. In (**D**), N = 51 (Control RNAi) and 35 (*lem-2(RNAi)*) early pachytene nuclei pooled from seven or four animals were measured. $p < 2.2\text{e-}16$ between the control RNAi and *lem-2(RNAi)* with 2-way ANOVA. See **Data S1**. All worms were homozygous for $P_{gld\text{-}1}$*::TIR1*.

Fig. S18.

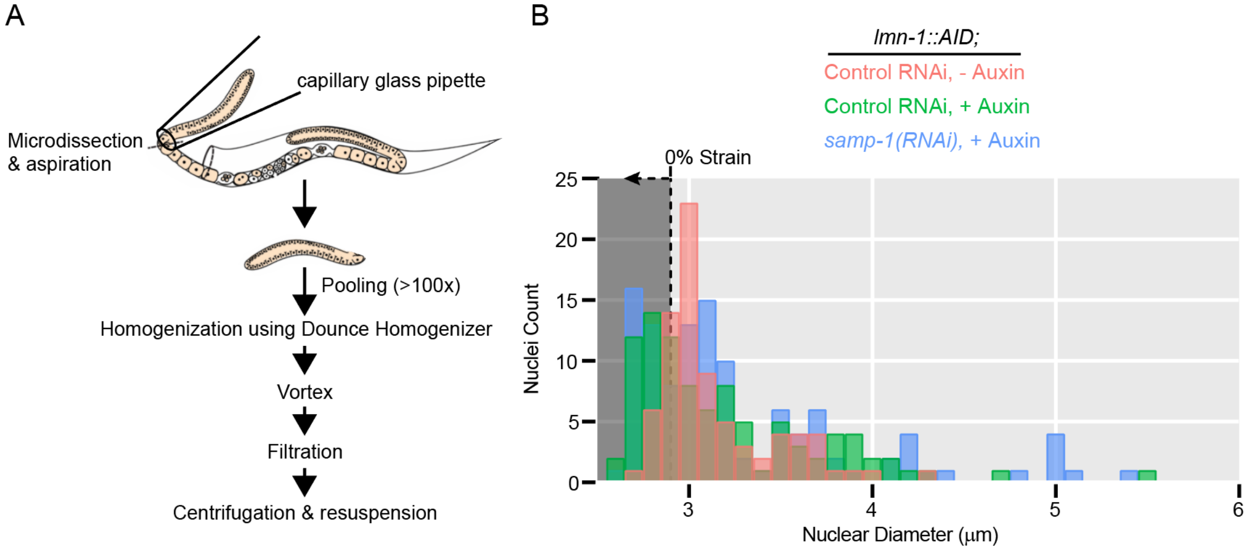
A
capillary glass pipette
Microdissection
& aspiration
Pooling (>100x)
Homogenization using Dounce Homogenizer
Vortex
Filtration
Centrifugation & resuspension
B
lmn-1::AID;
Control RNAi, - Auxin
Control RNAi, + Auxin
samp-1(RNAi), + Auxin
0% Strain
Nuclei Count
Nuclear Diameter (µm)
25
20
15
10
5
0
3
4
5
6

Fig. S18. (Related to Fig. 5) Isolating meiotic nuclei for stiffness measurement using mechano-NPS. (**A**) Schematic of the workflow of microdissecting *C. elegans* gonads to isolate meiotic nuclei. (**B**) Nuclear size measured with mechano-NPS. The nuclear size is calculated using **supplemental equation 1**. Welch ANOVA with Games-Howell multiple comparisons test revealed no significant differences between nuclear diameter in each group, $p>0.05$. Sample sizes for the Control RNAi, -Auxin, the Control RNAi, +Auxin, and the *samp-1(RNAi)*, +Auxin were n=79, n=94, n=113 nuclei respectively. The darker region of the histogram to the left of the dotted line contains nuclei which were excluded from *wCDI* analysis because they were too small to experience any strain, i.e. their size was smaller than or equal to the width of the contraction channel employed. All nuclei to the right of the dotted line were included in *wCDI* analysis.

Table S1.

Strains	Auxin	n	Embryos laid (±SD)	Embryonic viability (±SD) %*	Male progeny (±SD) %
P_{sun-1}::TIR1IV	-	6	225.5 ± 18.9	106.1 ± 3.2	0.0 ± 0.0
P_{sun-1}::TIR1	+ (from L1)	4	228.0 ± 26.5	106.8 ± 3.8	0.0 ± 0.0
P_{sun-1}::TIR1 IV	+ (from L4)	3	245.7 ± 43.8	108.0 ± 2.3	0.1 ± 0.2
lmn-1::AID::V5 I; P_{sun-1}::TIR1 IV	-	7	252.1 ± 30.4	106.4 ± 5.1	0.1 ± 0.2
lmn-1::AID::V5 I; P_{sun-1}::TIR1 IV	+ (from L1)	6	83.3 ± 45.8	9.6 ± 23.5	0.0 ± 0.0
lmn-1::AID::V5 I; P_{sun-1}::TIR1 IV	+ (from L4)	3	39.7 ± 44.0	0.0 ± 0.0	0.0 ± 0.0
HA::AID::zyg-12 II; P_{sun-1}::TIR1 IV	-	3	254.3 ± 76.4	104.1 ± 4.7	0.0 ± 0.0
HA::AID::zyg-12 II; P_{sun-1}::TIR1 IV	+ (from L1)	3	0.0 ± 0.0	0.0 ± 0.0	0.0 ± 0.0
HA::AID::zyg-12 II; P_{sun-1}::TIR1 IV	+ (from L4)	3	0.0 ± 0.0	0.0 ± 0.0	0.0 ± 0.0
sun-1::AID::V5 V; P_{sun-1}::TIR1 IV	-	6	225.5 ± 53.5	81.3 ± 21.7	0.7 ± 0.7
sun-1::AID::V5 V; P_{sun-1}::TIR1 IV	+ (from L1)	5	31.0 ± 26.5	0.0 ± 0.0	0.0 ± 0.0
sun-1::AID::V5 V; P_{sun-1}::TIR1 IV	+ (from L4)	3	33.3 ± 35.3	0.0 ± 0.0	0.0 ± 0.0
emr-1(gk119) I; lem-2::HA::AID II; P_{sun-1}::TIR1 IV	-	6	216.3 ± 22.5	106.4 ± 2.4	0.1 ± 0.2

* Embryonic viability of >100% reflects the fact that some embryos are overlooked when counting, but adult worms hatched from these embryos are easier to count accurately.

Table S1. Quantification of brood size, embryonic viability, and male self-progeny of worm strains with AID alleles generated in this study. Sample size n indicates the number of broods examined. Note that high levels of uncertainty are usually associated with counting deformed eggs resulting from auxin treatment.

Table S2.

Allele	Genotype	Information about mutagenesis
ie137	*lmn-1(ie137[lmn-1::AID::V5])*	Internally tagged; generated using *dpy-10* Co-CRISPR in *ieSi38[sun-1p::TIR1::mRuby::sun-1 3'UTR, Cbr-unc-119(+)] IV*
ie138	*zyg-12(ie138[HA::AID::zyg-12])*	generated using *dpy-10* Co-CRISPR in *ieSi38[sun-1p::TIR1::mRuby::sun-1 3'UTR, Cbr-unc-119(+)] IV*
ie139	*sun-1(ie139[sun-1::AID::V5])*	Internally tagged; generated using *dpy-10* Co-CRISPR in *ieSi38[sun-1p::TIR1::mRuby::sun-1 3'UTR, Cbr-unc-119(+)] IV*
ie140	*lem-2(ie140[lem-2::HA::AID])*	generated using *dpy-10* Co-CRISPR in *ieSi38[sun-1p::TIR1::mRuby::sun-1 3'UTR, Cbr-unc-119(+)] IV*
ie203	*atg-7(ie203[atg-7::AID::HA])*	generated using *dpy-10* Co-CRISPR in *ieSi38[sun-1p::TIR1::mRuby::sun-1 3'UTR, Cbr-unc-119(+)] IV*

Table S2. Alleles generated in this study.

Table S3.

Strains	Source	Identifier
C. elegans: N2 Bristol, wild isolate	Caenorhabditis Genetics Center	N2
C.elegans: bcIs39(ced-1::GFP) V	Caenorhabditis Genetics Center	CA195 (MD701)
C. elegans: dnc-1::GFP	Zhang, Skop and White (89)	CA846
C.elegans: syp-1 (me17) bcIs39(ced-1::GFP) V/ nT1 [qIs51] (IV;V)	Bhalla et al. (43)	CA885
C.elegans: ieDf2/mIs11 IV	Harper et al. (57); Caenorhabditis Genetics Center	CA998
C. elegans: ieSi38[sun-1p::TIR1::mRuby::sun-1 3' UTR, Cbr-unc-119 (+)] IV	Zhang et al. (35); Caenorhabditis Genetics Center	CA1199
C.elegans: ieSi64[gld-1p::TIR1::mRuby::gld-1 3'UTR, Cbr-unc-119(+)] II; unc-119(ed3) III	Zhang et al. (35); Caenorhabditis Genetics Center	CA1352
C.elegans: ieSi65[sun-1p::TIR1::sun-1 3'UTR, Cbr-unc-119(+)] II; unc-119(ed3) III	Zhang et al. (35); Caenorhabditis Genetics Center	CA1353
C. elegans: meIs8[pie-1p::GFP::cosa-1, unc-119(+)] II; spo-11(ie59[spo-11::AID::3xFLAG]), ieSi38[sun-1p::TIR1::mRuby::sun-1 3'UTR, Cbr-unc-119(+)] IV	Zhang et al. (82); Caenorhabditis Genetics Center	CA1423
C. elegans: lmn-1(ie137[lmn-1::AID::V5]) I; unc-119 (ed3) III; ieSi38 [P_{sun-1}::TIR1::mRuby::sun-1 3'UTR, cb-unc-119(+)] IV	This paper	CA1532
C. elegans: lmn-1(ie137[lmn-1::AID::V5]) I; unc-119 (ed3) III; ieSi38 [P_{sun-1}::TIR1::mRuby::sun-1 3'UTR, cb-unc-119(+)] IV; bcIs39 (ced-1::GFP) V	This paper	CA1561
C. elegans: lmn-1(ie137[lmn-1::AID::V5]) I; ;ced-4(n1162) III; ieSi38 [P_{sun-1}::TIR1::mRuby::sun-1 3'UTR, cb-unc-119(+)] IV	This paper	CA1562
C. elegans: lmn-1(ie137[lmn-1::AID::V5]) I; ieSi65[sun-1p::TIR1::sun-1 3'UTR, Cbr-unc-119(+)] II; unc-119(ed3) III; ieSi21 (sun-1::mRuby) IV	This paper	CA1563
C. elegans: unc-119 (ed3) III; ieSi38 [P_{sun-1}::TIR1::mRuby::sun-1 3'UTR, cb-unc-119(+)] IV; sun-1(ie139[sun-1::AID::V5]) V	This paper	CA1564
C. elegans: lmn-1(ie137[lmn-1::AID::V5]) I; ieSi64[gld-1p::TIR1::mRuby::gld-1 3'UTR, Cbr-unc-119(+)] II; unc-119(ed3) III; sun-1(ie139[sun-1::AID::V5]) V	This paper	CA1565
C. elegans: zyg-12(ie138[HA::AID::zyg-12]) II; unc-119 (ed3) III; ieSi38 [P_{sun-1}::TIR1::mRuby::sun-1 3'UTR, cb-unc-119(+)] IV	This paper	CA1566
C. elegans: lmn-1(ie137[lmn-1::AID::V5]) I; zyg-12(ie138[HA::AID::zyg-12]) II; unc-119 (ed3) III; ieSi38 [P_{sun-1}::TIR1::mRuby::sun-1 3'UTR, cb-unc-119(+)] IV	This paper	CA1567
C. elegans: lmn-1(ie137[lmn-1::AID::V5]), emr-1(gk119) I; unc-119 (ed3) III; ieSi38 [P_{sun-1}::TIR1::mRuby::sun-1 3'UTR, cb-unc-119(+)] IV	This paper	CA1568
C. elegans: lem-2(ie140[lem-2::HA::AID]) II; ieSi64[gld-1p::TIR1::mRuby::gld-1 3'UTR, Cbr-unc-119(+)] II; unc-119(ed3) III;	This paper	CA1569
C. elegans: lmn-1(ie137[lmn-1::AID::V5]) I; lem-2(ie140[lem-2::HA::AID]), ieSi64[gld-1p::TIR1::mRuby::gld-1 3'UTR, Cbr-unc-119(+)] II; unc-119(ed3) III;	This paper	CA1570
C. elegans: lmn-1(ie137[lmn-1::AID::V5]), emr-1(gk119) I; unc-119 (ed3) III; ieSi38 [P_{sun-1}::TIR1::mRuby::sun-1 3'UTR, cb-unc-119(+)] IV; sun-1(ie139[sun-1::AID::V5]) V	This paper	CA1571
C. elegans: syp-3 (ok857) I; ieSi64[gld-1p::TIR1::mRuby::gld-1 3'UTR, Cbr-unc-119(+)] II; unc-119(ed3) III; ieSi19 (mRuby::SYP-3),ojIs9 [zyg-12(all)::GFP + unc-119(+)] IV; sun-1(ie139[sun-1::AID::V5]) V	This paper	CA1572
C. elegans: lmn-1(ie137[lmn-1::AID::V5]) I; ieSi64[gld-1p::TIR1::mRuby::gld-1 3'UTR, Cbr-unc-119(+)] II; unc-119(ed3) III; ieSi19 (mRuby::SYP-3),ojIs9 [zyg-12(all)::GFP + unc-119(+)] IV; sun-1(ie139[sun-1::AID::V5]) V	This paper	CA1573
C. elegans: lmn-1(ie137[lmn-1::AID::V5]) I; ieSi64[gld-1p::TIR1::mRuby::gld-1 3'UTR, Cbr-unc-119(+)] II; unc-119(ed3) III; ieSi19 (mRuby::SYP-3),ojIs9 [zyg-12(all)::GFP + unc-119(+)] IV;	This paper	CA1574
C. elegans: lmn-1(ie137[lmn-1::AID::V5]), emr-1(gk119) I; ieSi64[gld-1p::TIR1::mRuby::gld-1 3'UTR, Cbr-unc-119(+)] II; unc-119(ed3) III; ieSi19 (mRuby::SYP-3),ojIs9 [zyg-12(all)::GFP + unc-119(+)] IV;	This paper	CA1575
C. elegans: lmn-1(ie137[lmn-1::AID::V5]) I; meIs8[pie-1p::GFP::cosa-1, unc-119(+)] II; unc-119 (ed3) III; ieSi38 [P_{sun-1}::TIR1::mRuby::sun-1 3'UTR, cb-unc-119(+)] IV	This paper	CA1576
C. elegans: lmn-1(ie137[lmn-1::AID::V5]) I; meIs8[pie-1p::GFP::cosa-1, unc-119(+)] II; unc-119 (ed3) III; spo-11(ie59[spo-11::AID::3xFLAG]), ieSi38 [P_{sun-1}::TIR1::mRuby::sun-1 3'UTR, cb-unc-119(+)] IV	This paper	CA1577

C. elegans: lmn-1(ie137[lmn-1::AID::V5]) I; unc-119 (ed3) III; spo-11(ie59[spo-11::AID::3xFLAG]), ieSi38 [$P_{sun\text{-}1}$::TIR1::mRuby::sun-1 3'UTR, cb-unc-119(+)] IV; sun-1(ie139[sun-1::AID::V5]) V	This paper	CA1578
C. elegans: zyg-12(ie138[HA::AID::zyg-12]) II; unc-119 (ed3) III; ieSi38 [$P_{sun\text{-}1}$::TIR1::mRuby::sun-1 3'UTR, cb-unc-119(+)] IV; bcIs39 (ced-1::GFP) V	This paper	CA1579
C. elegans: emr-1(gk119) I; lem-2(ie140[lem-2::HA::AID]) II; ieSi64[gld-1p::TIR1::mRuby::gld-1 3'UTR, Cbr-unc-119(+)] II; unc-119(ed3) III;	This paper	CA1677
C. elegans: mjl-1(tm1651) I; hT2 [bli-4(e937) let-?(q782) qIs48] (I,III)	National Bioresource Project	CA1728
C. elegans: unc-119 (ed3) III; atg-7(ie203[atg-7::AID::HA]) IV; ieSi38 [$P_{sun\text{-}1}$::TIR1::mRuby::sun-1 3'UTR, cb-unc-119(+)] IV	This paper	CA1729

Table S3. Genotypes of worm strains generated and used in this study.

Table S4.

Transgenes	crRNAs and repair templates (mostly gBlock)	Genotyping primer names	Primer sequences	Fragment sizes
lmn-1::AID::V5 in *ie137*	5' – AGAAGTTCGTCACAAGAGAC – 3'; 5' - TCTGGAAGAAGATCTCGCTTTTGCTCTTCAA CAGCACAAGGGAGAACTTGAAGAAGTTCGcC ACAAGAGgCAGGTCGACATGACAACCTACG GCGGCGGAGGATCCatgcctaaagatccagccaaac ctccggccaaggcacaagttgtgggatggccaccggtgagatc ataccggaagaacgtgatggtttcctgccaaaaatcaagcggtg gcccggaggcggcggcgttcgtgaagggaggatccggaGG AAAGCCAATTCCAAACCCACTTCTTGGACTC GACTCCACCGCCAAGCAGATTAATGATGAGT ATCAATCTAAGCTT -3'	oCL41 (F)	5' - AAA GCA GAA CAT CAC TCT TCG TGA CAC CGT AGA AG -3';	WT, 277bp; inserted, 481bp
		oCL42 (R)	5' - TTT GAT GCA AAT TGT TCT TGA ACT GAG CAC GCA TCT C -3'	
HA::AID::zyg-12 in *ie138*	5' – GAATCTGAGTCGTCAGACAA – 3'; 5' – aaaatctatcaatttcttttttttcagaacaaaatcatgTACCCAT ACGATGTTCCAGATTACGCTggaggatccggaatg cctaaagatccagccaaacctccggccaaggcacaagttgtgg gatggccaccggtgagatcataccggaagaacgtgatggtttcct gccaaaaatcaagcggtggcccggaggcggcggcgttcgtga agGGCGGCGGAGGATCCGGAGGAGGAGGC AGTGGAGGCGGCGGTTCTGGCGGTGGCGG CTCAGGCGGAGGTGGATCGTTAGACCTGAC AAACAAAGAGTCCGAGTCTTCAGACAACGGA AATAGCAAGTACGAAGATTCCATAGACGGAC GA – 3'	oCL95 (F)	5' – TTGTAAACTCTACCAGCC T -3';	WT, 401bp; inserted, 650bp
		oCL96 (R)	5' – TCAGAGGTAGTTTAGTGG C -3';	
sun-1::AID::V5 in *ie139*	5' – GCTGGAATATCGCATTCGCA – 3'; 5' – TACAAGGAGCATTTTAGCTACAAAGAAATCA CTTCGATGAAGAAGGAAATGTGGTATGACTG GCTGGAATATCGCATcCGtGGCGGCGGAGGA TCCatgcctaaagatccagccaaacctccggccaaggcaca agttgtgggatggccaccggtgagatcataccggaagaacgtg atggtttcctgccaaaaatcaagcggtggcccggaggcggcgg cgttcgtgaagggaggatccggaGGAAAGCCAATTCC AAACCCACTTCTTGGACTCGACTCCACCATG GTTCGGCGTCGTTTTGTTCCAACGTGGGCC CAGTTTAAACGTACTCTT – 3'	oCL101 (F)	5'- CTT CGA TGA AGA AGG AAA TGT GGT ATG ACT GGC -3';	WT, 456bp; inserted, 660bp
		oCL102 (R)	5'- CTC TTC GAT TGC CGA CTC TTT CCA TCC TTT -3';	
lem-2::HA::AID in *ie140*	5' – TGTGCCGTGTGGAAGTGGAT – 3'; 5' – CTACCGATGTTCTTGTGCTTCCGTCTGGAAA TGAGTGcGCtGTcTGGAAaTGGATCGGAAATC AGTCTCAGAAGAGATGGTACCCATACGATGT TCCAGATTACGCTggaggatccggaatgcctaaagatc cagccaaacctccggccaaggcacaagttgtgggatggccacc ggtgagatcataccggaagaacgtgatggtttcctgccaaaaat caagcggtggcccggaggcggcggcgttcgtgaagTAGatc attgttttgctgtataatttttcgatttt– 3'	oCL137 (F)	5' – GAAGCTCTACGAGCTCAT C – 3';	WT, 379bp; inserted, 553bp
		oCL138 (R)	5' – gtcattgtgataccttaggc – 3';	
atg-7::AID::HA in ie203	5' – GATGATGAAGATTTCTGAat – 3'; 5' – CAGAACTCTGTTAATGCTATTGATATCGATTT TGAGGATGATGAAGATTTCGGCGGCGGAGG ATCCGGAGGAGGAGGCAGTGGAGGCGGCG GTTCTGGCGGTGGCGGCTCAatgcctaaagatcca gccaaacctccggccaaggcacaagttgtgggatggccaccg gtgagatcataccggaagaacgtgatggtttcctgccaaaaatc aagcggtggcccggaggcggcggcgttcgtgaagggaggatc cggaTACCCATACGATGTTCCAGATTACGCTT GAattggtcgcctcaaatttttaccttttctgtataattg – 3'	Atg-7-Fw (F)	5' – GTCGTCTCGAAGAAGTCA C – 3';	WT, 186bp; inserted, 420bp
		Atg-7-Rw (R)	5' – ggaggcaaaatagaatcac – 3';	
dpy-10	5' – GCTACCATAGGCACCACGAG – 3'; 5' – ATACGGCAAGATGAGAATGACTGGAAACCGT ACCGCATGCGGTGCCTATGGTAGCGGAGCT TCACATGGCTTCAGA – 3' (ssDNA repair template)	N.A.	N.A.	N.A.

Table S4. Sequences of crRNAs, repair templates, and DNA primers used to genotype edited progeny.

Table S5.

Target gene	GenePairs Name	Plate	Well
dnc-1	ZK593.5	111	B12
dlc-1	T26A5.9	75	H6
lis-1	T03F6.5	88	F4
dhc-1	T21E12.4	4	H2
dli-1	C39E9.14	116	A3
dylt-1	F13G3.4	11	E6
lem-2	W01G7.5	63	B2
samp-1	T24F1.2	58	A6
lmn-1	DY3.2	14	D12

Table S5. RNAi clones used in this study.

Table S6.

	$\Delta I_S/I$	σ_I	D_e	n
Channel A	5.00×10^{-5}	0.768×10^{-5}	10.2	25
Channel B	4.23×10^{-5}	0.719×10^{-5}	11.1	21

Table S6. Measuring channel effective diameters. Polystyrene microspheres (Sigma-Aldrich) with a diameter of 2 µm ± 0.05 µm suspended in the 1x Wash Buffer were measured with the mechano-NPS platform. One platform consisted of two independent microfluidic channels for measurement (Channel A and B), which acted as two independent devices. Polystyrene beads were measured in both devices to calculate their effective diameters. $\boldsymbol{\Delta I_S/I}$ corresponds to the average ratio of current pulse amplitude to baseline current produced as the polystyrene beads transited the sizing segment and $\boldsymbol{\sigma_I}$ corresponds to the standard deviation. D_e is the effective diameter, as calculated from Equation S1 using $\boldsymbol{\Delta I_S/I}$ and the known diameter of the polystyrene beads. n corresponds to the number of beads measured in each device.

Table S7.

	U_{flow} (µm/msec)	σ_U (µm/msec)	*n*
Channel A	10.84	0.80	109
Channel B	10.15	1.23	68

Table S7. Measuring fluid velocity. U_{flow} is the approximate fluid velocity (µm/msec), σ_U is its standard deviation (µm/msec), and *n* is the number of nuclei measured in each device (Channel A and B). The U_{flow} in a device is the average nuclear velocity in the sizing segment of all nuclei measured in that device (**Equation S3**). One platform consisted of two independent microfluidic channels for measurement, Channel A and B, which acted as independent devices, therefore U_{flow} was calculated for each device. Three replicas of both Channel A and B were used to calculate the U_{flow} values reported. Only nuclei whose *wCDI* was measured (nuclei that underwent > 0% strain) were included in the U_{flow} calculation for each device.

Equations

Particle size was calculated using,

$$\frac{\Delta I_S}{I} = \frac{d^3}{D_e^2 L}\left[\frac{1}{1-0.8\left(\frac{d}{D_e}\right)^3}\right] \quad \text{(Eq. S1)}$$

where $\Delta I_S$is the magnitude of the current drop produced by the particle as it transits the sizing segment (**Fig. 5B**), I is the baseline current, d is the diameter of the particle, L is the overall channel length, and D_e is the effective diameter (Table S6) *(96).*

The whole-cell deformability index (*wCDI*) was calculated using,

$$wCDI = \frac{v_c}{U_{flow}}\left(\frac{d_n}{h}\right) \quad \text{(Eq. S2)}$$

where d_n is the nuclear diameter, h is the channel height, v_c is the velocity of the nucleus in the contraction segment, and U_{flow} is the fluid velocity *(67)*. The contraction segment velocity is defined as $v_c = L_c/t_c$, where L_c is the contraction segment length and t_c is the nuclear transit time in the contraction segment. The fluid velocity, U_{flow}, can be approximated as the average nuclear velocity in the sizing segment,

$$U_{flow} \sim v_{s\_avg} = \frac{\sum v_s}{n} \quad \text{(Eq. S3)}$$

where v_s is the nuclear velocity in the sizing segment and n is the number of nuclei measured. The sizing segment velocity is defined as $v_s = L_s/t_s$, where L_s is the sizing segment length and t_s is the nuclear transit time through the sizing segment. An average of all nuclei's v_s is used to calculate U_{flow} to take into account variations in the fluid velocity due to off-axis hydrodynamic effects *(97)*. For the experiments reported here, we calculated U_{flow} for each device (Channel A and B) utilized (**Table S7**).

Movie S1. (separate file)

Nuclear envelope dynamics marked by SUN-1::mRuby in late prophase after LMN-1 depletion. Two representative time-lapse recordings of SUN-1::mRuby at the NE of meiotic nuclei in late meiotic prophase after LMN-1 depletion are shown. Arrow points to a collapsing nucleus. Note many meiotic nuclei with asymmetrically distributed SUN-1::mRuby at the NE. Time stamp is hr:min:sec. Scale bars, 10 μm.

Movie S2. (separate file)

Tracking SUN-1::mRuby patches in early meiosis. An example of drift-corrected time-lapse recordings of SUN-1::mRuby patch movement on the NE of transition zone nuclei, using reference frame followed by 3D particle tracking in Imaris. Time stamp is hr:min:sec. Scale bar, 5 μm.

Movie S3. (separate file)

LMN-1 depletion changes the mobility of SUN-1::mRuby patches throughout prophase. Side-by-side comparison of time-lapse recordings of the movement of SUN-1::mRuby patches or foci during different stages of meiosis, with or without LMN-1 depletion. Time stamp is hr:min:sec. Scale bars, 5 μm.

Movie S4. (separate file)

Dynamics of diplotene nuclear collapse. Time-lapse recording of a dual-color labeled oocyte nucleus during diplotene collapse. Green, ZYG-12::GFP; Magenta, mRuby::SYP-3. Scale bar, 5 μm.

Movie S5. (separate file)

Contact between NE and SC happens during diplotene nuclear collapse. Time-lapse recording of another dual-color labeled diplotene nucleus during collapse. The frame showing initial contact between NE and SC is annotated. Green, ZYG-12::GFP; Magenta, mRuby::SYP-3. Time stamp is hr:min:sec. Scale bar, 2 μm.

Data S1. (separate file)

Statistical source data. Additional output of statistical tests performed in this study (with related figures or supplementary figure numbers).

REFERENCES AND NOTES

1. N. Bhalla, A. F. Dernburg, Prelude to a division. *Annu. Rev. Cell Dev. Biol.* **24**, 397–424 (2008).

2. Z. Yu, Y. Kim, A. F. Dernburg, Meiotic recombination and the crossover assurance checkpoint in Caenorhabditis elegans. *Semin. Cell Dev. Biol.* **54**, 106–116 (2016).

3. A. Sato, B. Isaac, C. M. Phillips, R. Rillo, P. M. Carlton, D. J. Wynne, R. A. Kasad, A. F. Dernburg, Cytoskeletal forces span the nuclear envelope to coordinate meiotic chromosome pairing and synapsis. *Cell* **139**, 907–919 (2009).

4. D. J. Wynne, O. Rog, P. M. Carlton, A. F. Dernburg, Dynein-dependent processive chromosome motions promote homologous pairing in C. elegans meiosis. *J. Cell Biol.* **196**, 47–64 (2012).

5. C.-Y. Lee, H. F. Horn, C. L. Stewart, B. Burke, E. Bolcun-Filas, J. C. Schimenti, M. E. Dresser, R. J. Pezza, Mechanism and regulation of rapid telomere prophase movements in mouse meiotic chromosomes. *Cell Rep.* **11**, 551–563 (2015).

6. J. Link, M. Leubner, J. Schmitt, E. Göb, R. Benavente, K. T. Jeang, R. Xu, M. Alsheimer, Analysis of meiosis in SUN1 deficient mice reveals a distinct role of SUN2 in mammalian meiotic LINC complex formation and function. *PLOS Genet.* **10**, e1004099 (2014).

7. Y. Luo, I.-W. Lee, Y.-J. Jo, S. Namgoong, N.-H. Kim, Depletion of the LINC complex disrupts cytoskeleton dynamics and meiotic resumption in mouse oocytes. *Sci. Rep.* **6**, 20408 (2016).

8. B. Burke, LINC complexes as regulators of meiosis. *Curr. Opin. Cell Biol.* **52**, 22–29 (2018).

9. W. Chang, H. J. Worman, G. G. Gundersen, Accessorizing and anchoring the LINC complex for multifunctionality. *J. Cell Biol.* **208**, 11–22 (2015).

10. J. Fan, H. Jin, B. A. Koch, H.-G. Yu, Mps2 links Csm4 and Mps3 to form a telomere-associated LINC complex in budding yeast. *Life Sci. Alliance* **3**, e202000824 (2020).

11. H. J. Kim, C. Liu, A. F. Dernburg, How and why chromosomes interact with the cytoskeleton during meiosis. *Genes* 2022;**13**:901.

12. K. Zhou, M. M. Rolls, D. H. Hall, C. J. Malone, W. Hanna-Rose, A ZYG-12–dynein interaction at the nuclear envelope defines cytoskeletal architecture in the C. elegans gonad. *J. Cell Biol.* **186**, 229–241 (2009).

13. O. Rog, Abby F. Dernburg, Direct visualization reveals kinetics of meiotic chromosome synapsis. *Cell Rep.* **10**, 1639–1645 (2015).

14. M. Zhang, in *Encyclopedia of Reproduction*, M. K. Skinner, Ed. (Academic Press, ed. 2, 2018), pp. 153–158.

15. G. Nagamatsu, S. Shimamoto, N. Hamazaki, Y. Nishimura, K. Hayashi, Mechanical stress accompanied with nuclear rotation is involved in the dormant state of mouse oocytes *Sci. Adv.* **5**, eaav9960 (2019).

16. Y. Tsatskis, R. Rosenfeld, J. D. Pearson, C. Boswell, Y. Qu, K. Kim, L. Fabian, A. Mohammad, X. Wang, M. I. Robson, K. Krchma, J. Wu, J. Gonçalves, D. Hodzic, S. Wu, D. Potter, L. Pelletier, W. H. Dunham, A. C. Gingras, Y. Sun, J. Meng, D. Godt, T. Schedl, B. Ciruna, K. Choi, J. R. B. Perry, R. Bremner, E. C. Schirmer, J. A. Brill, A. Jurisicova, H. McNeill, The NEMP family supports metazoan fertility and nuclear envelope stiffness *Sci. Adv.* **6**, eabb4591 (2020).

17. A. E. Goldman, G. Maul, P. M. Steinert, H. Y. Yang, R. D. Goldman, Keratin-like proteins that coisolate with intermediate filaments of BHK-21 cells are nuclear lamins. *Proc. Natl. Acad. Sci. U.S.A.* **83**, 3839–3843 (1986).

18. U. Aebi, J. Cohn, L. Buhle, L. Gerace, The nuclear lamina is a meshwork of intermediate-type filaments. *Nature* **323**, 560–564 (1986).

19. F. D. McKeon, M. W. Kirschner, D. Caput, Homologies in both primary and secondary structure between nuclear envelope and intermediate filament proteins. *Nature* **319**, 463–468 (1986).

20. P. M. Davidson, J. Lammerding, Broken nuclei—Lamins, nuclear mechanics, and disease. *Trends Cell Biol.* **24**, 247–256 (2014).

21. J. Swift, I. L. Ivanovska, A. Buxboim, T. Harada, P. C. D. P. Dingal, J. Pinter, J. D. Pajerowski, K. R. Spinler, J. W. Shin, M. Tewari, F. Rehfeldt, D. W. Speicher, D. E. Discher, Nuclear lamin-A scales with tissue stiffness and enhances matrix-directed differentiation. *Science* **341**, 1240104 (2013).

22. J. L. V. Broers, F. C. S. Ramaekers, G. Bonne, R. B. Yaou, C. J. Hutchison, Nuclear lamins: Laminopathies and their role in premature ageing. *Physiol. Rev.* **86**, 967–1008 (2006).

23. H. J. Worman, Nuclear lamins and laminopathies. *J. Pathol.* **226**, 316–325 (2012).

24. C. M. Denais, R. M. Gilbert, P. Isermann, A. L. McGregor, M. te Lindert, B. Weigelin, P. M. Davidson, P. Friedl, K. Wolf, J. Lammerding, Nuclear envelope rupture and repair during cancer cell migration. *Science* **352**, 353–358 (2016).

25. A. J. Earle, T. J. Kirby, G. R. Fedorchak, P. Isermann, J. Patel, S. Iruvanti, S. A. Moore, G. Bonne, L. L. Wallrath, J. Lammerding, Mutant lamins cause nuclear envelope rupture and DNA damage in skeletal muscle cells. *Nat. Mater.* **19**, 464–473 (2020).

26. E. M. Hatch, A. H. Fischer, T. J. Deerinck, M. W. Hetzer, Catastrophic nuclear envelope collapse in cancer cell micronuclei. *Cell* **154**, 47–60 (2013).

27. M. Kneissig, K. Keuper, M. S. de Pagter, M. J. van Roosmalen, J. Martin, H. Otto, V. Passerini, A. Campos Sparr, I. Renkens, F. Kropveld, A. Vasudevan, J. M. Sheltzer, W. P. Kloosterman, Z. Storchova, Micronuclei-based model system reveals functional consequences of chromothripsis in human cells. *eLife* **8**, e50292 (2019).

28. J. Link, D. Paouneskou, M. Velkova, A. Daryabeigi, T. Laos, S. Labella, C. Barroso, S. Pacheco Piñol, A. Montoya, H. Kramer, A. Woglar, A. Baudrimont, S. M. Markert, C. Stigloher, E. Martinez-Perez, A. Dammermann, M. Alsheimer, M. Zetka, V. Jantsch, Transient and partial nuclear lamina disruption promotes chromosome movement in early meiotic prophase. *Dev. Cell* **45**, 212–225.e7 (2018).

29. J. Link, D. Jahn, J. Schmitt, E. Göb, J. Baar, S. Ortega, R. Benavente, M. Alsheimer, The meiotic nuclear lamina regulates chromosome dynamics and promotes efficient homologous recombination in the mouse. *PLOS Genet.* **9**, e1003261 (2013).

30. M. C. Vantyghem, D. Vincent-Desplanques, F. Defrance-Faivre, J. Capeau, C. Fermon, A. S. Valat, O. Lascols, A. C. Hecart, P. Pigny, B. Delemer, C. Vigouroux, J. L. Wemeau, Fertility and obstetrical complications in women with LMNA-related familial partial lipodystrophy. *J. Clin. Endocrinol. Metabol.* **93**, 2223–2229 (2008).

31. J. Liu, T. R. Ben-Shahar, D. Riemer, M. Treinin, P. Spann, K. Weber, A. Fire, Y. Gruenbaum, Essential roles for Caenorhabditis elegans lamin gene in nuclear organization, cell cycle progression, and spatial organization of nuclear pore complexes. *Mol. Biol. Cell* **11**, 3937–3947 (2000).

32. R. A. Green, H. L. Kao, A. Audhya, S. Arur, J. R. Mayers, H. N. Fridolfsson, M. Schulman, S. Schloissnig, S. Niessen, K. Laband, S. Wang, D. A. Starr, A. A. Hyman, T. Schedl, A. Desai, F. Piano, K. C. Gunsalus, K. Oegema, A high-resolution C. elegans essential gene network based on phenotypic profiling of a complex tissue. *Cell* **145**, 470–482 (2011).

33. G. Huelgas-Morales, M. Sanders, G. Mekonnen, T. Tsukamoto, D. Greenstein, Decreased mechanotransduction prevents nuclear collapse in a *Caenorhabditis elegans* laminopathy. *Proc. Natl. Acad. Sci. U.S.A.* **117**, 31301–31308 (2020).

34. E. Haithcock, Y. Dayani, E. Neufeld, A. J. Zahand, N. Feinstein, A. Mattout, Y. Gruenbaum, J. Liu, Age-related changes of nuclear architecture in *Caenorhabditis elegans*. *Proc. Natl. Acad. Sci. U.S.A.* **102**, 16690–16695 (2005).

35. L. Zhang, J. D. Ward, Z. Cheng, A. F. Dernburg, The auxin-inducible degradation (AID) system enables versatile conditional protein depletion in C. elegans. *Development* **142**, 4374–4384 (2015).

36. G. Velez-Aguilera, S. Nkombo Nkoula, B. Ossareh-Nazari, J. Link, D. Paouneskou, L. van Hove, N. Joly, N. Tavernier, J. M. Verbavatz, V. Jantsch, L. Pintard, PLK-1 promotes the merger of the parental genome into a single nucleus by triggering lamina disassembly. *eLife* **9**, e59510 (2020).

37. T. Dechat, S. A. Adam, P. Taimen, T. Shimi, R. D. Goldman, Nuclear lamins. *Cold Spring Harb. Perspect. Biol.* **2**, a000547 (2010).

38. C. Rinaldo, P. Bazzicalupo, S. Ederle, M. Hilliard, A. La Volpe, Roles for *Caenorhabditis elegans* rad-51 in meiosis and in resistance to ionizing radiation during development. *Genetics* **160**, 471–479 (2002).

39. R. Yokoo, K. A. Zawadzki, K. Nabeshima, M. Drake, S. Arur, A. M. Villeneuve, COSA-1 reveals robust homeostasis and separable licensing and reinforcement steps governing meiotic crossovers. *Cell* **149**, 75–87 (2012).

40. A. Penkner, L. Tang, M. Novatchkova, M. Ladurner, A. Fridkin, Y. Gruenbaum, D. Schweizer, J. Loidl, V. Jantsch, The nuclear envelope protein matefin/SUN-1 is required for homologous pairing in C. elegans meiosis. *Dev. Cell* **12**, 873–885 (2007).

41. T. Bohr, G. Ashley, E. Eggleston, K. Firestone, N. Bhalla, Synaptonemal complex components are required for meiotic checkpoint function in Caenorhabditis elegans. *Genetics* **204**, 987–997 (2016).

42. S. A. Raiders, M. D. Eastwood, M. Bacher, J. R. Priess, Binucleate germ cells in Caenorhabditis elegans are removed by physiological apoptosis. *PLOS Genet.* **14**, e1007417 (2018).

43. N. Bhalla, A. F. Dernburg, A conserved checkpoint monitors meiotic chromosome synapsis in Caenorhabditis elegans. *Science* **310**, 1683–1686 (2005).

44. T. Bohr, C. R. Nelson, E. Klee, N. Bhalla, Spindle assembly checkpoint proteins regulate and monitor meiotic synapsis in C. elegans. *J. Cell Biol.* **211**, 233–242 (2015).

45. A. J. MacQueen, M. P. Colaiácovo, K. McDonald, A. M. Villeneuve, Synapsis-dependent and -independent mechanisms stabilize homolog pairing during meiotic prophase in C. elegans. *Genes Dev.* **16**, 2428–2442 (2002).

46. A. Gartner, S. Milstein, S. Ahmed, J. Hodgkin, M. O. Hengartner, A conserved checkpoint pathway mediates DNA damage–induced apoptosis and cell cycle arrest in *C. elegans*. *Mol. Cell* **5**, 435–443 (2000).

47. Y. Zhang, L. Yan, Z. Zhou, P. Yang, E. Tian, K. Zhang, Y. Zhao, Z. Li, B. Song, J. Han, L. Miao, H. Zhang, SEPA-1 mediates the specific recognition and degradation of P granule components by autophagy in *C. elegans*. *Cell* **136**, 308–321 (2009).

48. Y. Tian, Z. Li, W. Hu, H. Ren, E. Tian, Y. Zhao, Q. Lu, X. Huang, P. Yang, X. Li, X. Wang, A. L. Kovács, L. Yu, H. Zhang, *C. elegans* screen identifies autophagy genes specific to multicellular organisms. *Cell* **141**, 1042–1055 (2010).

49. O. Rog, S. Köhler, A. F. Dernburg, The synaptonemal complex has liquid crystalline properties and spatially regulates meiotic recombination factors. *eLife* **6**, e21455 (2017).

50. I. L. Minn, M. M. Rolls, W. Hanna-Rose, C. J. Malone, SUN-1 and ZYG-12, mediators of centrosome–nucleus attachment, are a functional SUN/KASH pair in *Caenorhabditis elegans*. *Mol. Biol. Cell* **20**, 4586–4595 (2009).

51. M. Terasawa, M. Toya, F. Motegi, M. Mana, K. Nakamura, A. Sugimoto, *Caenorhabditis elegans* ortholog of the p24/p22 subunit, DNC-3, is essential for the formation of the dynactin complex by bridging DNC-1/p150^{Glued} and DNC-2/dynamitin. *Genes Cells* **15**, 1145–1157 (2010).

52. R. H. Harders, T. H. Morthorst, A. D. Lande, M. O. Hesselager, O. A. Mandrup, E. Bendixen, A. Stensballe, A. Olsen, Dynein links engulfment and execution of apoptosis via CED-4/Apaf1 in C. elegans. *Cell Death Dis.* **9**, 1012 (2018).

53. C. Liu, J.-Z. Chuang, C.-H. Sung, Y. Mao, A dynein independent role of Tctex-1 at the kinetochore. *Cell Cycle* **14**, 1379–1388 (2015).

54. S. M. O'Rourke, M. D. Dorfman, J. C. Carter, B. Bowerman, Dynein modifiers in C. elegans: Light chains suppress conditional heavy chain mutants. *PLOS Genet.* **3**, e128 (2007).

55. Y. B. Tzur, A. Margalit, N. Melamed-Book, Y. Gruenbaum, Matefin/SUN-1 is a nuclear envelope receptor for CED-4 during *Caenorhabditis elegans* apoptosis. *Proc. Natl. Acad. Sci. U.S.A.* **103**, 13397–13402 (2006).

56. C. M. Phillips, A. F. Dernburg, A family of zinc-finger proteins is required for chromosome-specific pairing and synapsis during meiosis in C. elegans. *Dev. Cell* **11**, 817–829 (2006).

57. N. C. Harper, R. Rillo, S. Jover-Gil, Z. J. Assaf, N. Bhalla, A. F. Dernburg, Pairing centers recruit a polo-like kinase to orchestrate meiotic chromosome dynamics in C. elegans. *Dev. Cell* **21**, 934–947 (2011).

58. H. J. Kim, C. Liu, L. Zhang, A. F. Dernburg, MJL-1 is a nuclear envelope protein required for homologous chromosome pairing and regulation of synapsis during meiosis in *C. elegans Sci. Adv.* **9**, eadd1453 (2023).

59. M. B. Gerstein, Z. J. Lu, E. L. van Nostrand, C. Cheng, B. I. Arshinoff, T. Liu, K. Y. Yip, R. Robilotto, A. Rechtsteiner, K. Ikegami, P. Alves, A. Chateigner, M. Perry, M. Morris, R. K. Auerbach, X. Feng, J. Leng, A. Vielle, W. Niu, K. Rhrissorrakrai, A. Agarwal, R. P. Alexander, G. Barber, C. M. Brdlik, J. Brennan, J. J. Brouillet, A. Carr, M. S. Cheung, H. Clawson, S. Contrino, L. O. Dannenberg, A. F. Dernburg, A. Desai, L. Dick, A. C. Dosé, J. du, T. Egelhofer, S. Ercan, G. Euskirchen, B. Ewing, E. A. Feingold, R. Gassmann, P. J. Good, P. Green, F. Gullier, M. Gutwein, M. S. Guyer, L. Habegger, T. Han, J. G. Henikoff, S. R. Henz, A. Hinrichs, H. Holster, T. Hyman, A. L. Iniguez, J. Janette, M. Jensen, M. Kato, W. J. Kent, E. Kephart, V. Khivansara, E. Khurana, J. K. Kim, P. Kolasinska-Zwierz, E. C. Lai, I. Latorre, A. Leahey, S. Lewis, P. Lloyd, L. Lochovsky, R. F. Lowdon, Y. Lubling, R. Lyne, M. MacCoss, S. D. Mackowiak, M. Mangone, S. McKay, D. Mecenas, G. Merrihew, D. M. Miller III, A. Muroyama, J. I. Murray, S. L. Ooi, H. Pham, T. Phippen, E. A. Preston, N. Rajewsky, G. Rätsch, H. Rosenbaum, J. Rozowsky, K. Rutherford, P. Ruzanov, M. Sarov, R. Sasidharan, A. Sboner, P. Scheid, E. Segal, H. Shin, C. Shou, F. J. Slack, C. Slightam, R. Smith, W. C. Spencer, E. O. Stinson, S. Taing, T. Takasaki, D. Vafeados, K. Voronina, G. Wang, N. L. Washington, C. M. Whittle, B. Wu, K. K. Yan, G. Zeller, Z. Zha, M. Zhong, X. Zhou; modENCODE Consortium, J. Ahringer, S. Strome, K. C. Gunsalus, G. Micklem, X. S. Liu, V. Reinke, S. K. Kim, L. D. W. Hillier, S. Henikoff, F. Piano, M. Snyder, L. Stein, J. D. Lieb, R. H. Waterston, Integrative analysis of the Caenorhabditis elegans genome by the modENCODE Project. *Science* **330**, 1775–1787 (2010).

60. K. Ikegami, T. A. Egelhofer, S. Strome, J. D. Lieb, Caenorhabditis elegans chromosome arms are anchored to the nuclear membrane via discontinuous association with LEM-2. *Genome Biol.* **11**, R120 (2010).

61. T. R. Mandigo, B. D. Turcich, A. J. Anderson, M. R. Hussey, E. S. Folker, *Drosophila* emerins control LINC complex localization and transcription to regulate myonuclear position. *J. Cell Sci.* **132**, jcs235580 (2019).

62. Y. Hiraoka, H. Maekawa, H. Asakawa, Y. Chikashige, T. Kojidani, H. Osakada, A. Matsuda, T. Haraguchi, Inner nuclear membrane protein Ima1 is dispensable for intranuclear positioning of centromeres. *Genes Cells* **16**, 1000–1011 (2011).

63. S. Gudise, R. A. Figueroa, R. Lindberg, V. Larsson, E. Hallberg, Samp1 is functionally associated with the LINC complex and A-type lamina networks. *J. Cell Sci.* **124**, 2077–2085 (2011).

64. J. Borrego-Pinto, T. Jegou, D. S. Osorio, F. Auradé, M. Gorjánácz, B. Koch, I. W. Mattaj, E. R. Gomes, Samp1 is a component of TAN lines and is required for nuclear movement. *J. Cell Sci.* **125**, 1099–1105 (2012).

65. C. R. Bone, E. C. Tapley, M. Gorjánácz, D. A. Starr, The Caenorhabditis elegans SUN protein UNC-84 interacts with lamin to transfer forces from the cytoplasm to the nucleoskeleton during nuclear migration. *Mol. Biol. Cell* **25**, 2853–2865 (2014).

66. A. Morales-Martínez, A. Dobrzynska, P. Askjaer, Inner nuclear membrane protein LEM-2 is required for correct nuclear separation and morphology in C. elegans. *J. Cell Sci.* **128**, 1090–1096 (2015).

67. J. Kim, S. Han, A. Lei, M. Miyano, J. Bloom, V. Srivastava, M. R. Stampfer, Z. J. Gartner, M. A. LaBarge, L. L. Sohn, Characterizing cellular mechanical phenotypes with mechano-node-pore sensing. *Microsyst. Nanoeng.* **4**, 17091 (2018).

68. B. Li, A. Maslan, S. E. Kitayama, C. Pierce, A. M. Streets, L. L. Sohn, Mechanical phenotyping reveals unique biomechanical responses in retinoic acid-resistant acute promyelocytic leukemia. *iScience* **25**, 103772 (2022).

69. K. G. Geles, S. A. Adam, Germline and developmental roles of the nuclear transport factor importin α3 in *C. elegans*. *Development* **128**, 1817–1830 (2001).

70. N. T. Chartier, A. Mukherjee, J. Pfanzelter, S. Fürthauer, B. T. Larson, A. W. Fritsch, R. Amini, M. Kreysing, F. Jülicher, S. W. Grill, A hydraulic instability drives the cell death decision in the nematode germline. *Nat. Phys.* **17**, 920–925 (2021).

71. A. F. Dernburg, Pushing the (nuclear) envelope into meiosis. *Genome Biol.* **14**, 110 (2013).

72. E. C. Schirmer, R. Foisner, Proteins that associate with lamins: Many faces, many functions. *Exp. Cell Res.* **313**, 2167–2179 (2007).

73. A. Brachner, S. Reipert, R. Foisner, J. Gotzmann, LEM2 is a novel MAN1-related inner nuclear membrane protein associated with A-type lamins. *J. Cell Sci.* **118**, 5797–5810 (2005).

74. L. Clements, S. Manilal, D. R. Love, G. E. Morris, Direct interaction between emerin and lamin A. *Biochem. Biophys. Res. Commun.* **267**, 709–714 (2000).

75. M. Sakaki, H. Koike, N. Takahashi, N. Sasagawa, S. Tomioka, K. Arahata, S. Ishiura, Interaction between emerin and nuclear lamins. *J. Biochem.* **129**, 321–327 (2001).

76. R. Zhu, C. Liu, G. G. Gundersen, Nuclear positioning in migrating fibroblasts. *Semin. Cell Dev. Biol.* **82**, 41–50 (2018).

77. E. S. Folker, C. Östlund, G. W. G. Luxton, H. J. Worman, G. G. Gundersen, Lamin A variants that cause striated muscle disease are defective in anchoring transmembrane actin-associated nuclear lines for nuclear movement. *Proc. Natl. Acad. Sci. U.S.A.* **108**, 131–136 (2011).

78. L. Penfield, B. Wysolmerski, M. Mauro, R. Farhadifar, M. A. Martinez, R. Biggs, H. Y. Wu, C. Broberg, D. Needleman, S. Bahmanyar, Dynein pulling forces counteract lamin-mediated nuclear stability during nuclear envelope repair. *Mol. Biol. Cell* **29**, 852–868 (2018).

79. W. Chang, Y. Wang, G. W. G. Luxton, C. Östlund, H. J. Worman, G. G. Gundersen, Imbalanced nucleocytoskeletal connections create common polarity defects in progeria and physiological aging. *Proc. Natl. Acad. Sci. U.S.A.* **116**, 3578–3583 (2019).

80. Y. K. Wu, H. Umeshima, J. Kurisu, M. Kengaku, Nesprins and opposing microtubule motors generate a point force that drives directional nuclear motion in migrating neurons. *Development* **145**, dev158782 (2018).

81. S. Brenner, The genetics of Caenorhabditis elegans. *Genetics* **77**, 71–94 (1974).

82. L. Zhang, S. Köhler, R. Rillo-Bohn, A. F. Dernburg, A compartmentalized signaling network mediates crossover control in meiosis. *eLife* **7**, e30789 (2018).

83. J. A. Arribere, R. T. Bell, B. X. H. Fu, K. L. Artiles, P. S. Hartman, A. Z. Fire, Efficient marker-free recovery of custom genetic modifications with CRISPR/Cas9 in *Caenorhabditis elegans*. *Genetics* **198**, 837–846 (2014).

84. A. Paix, A. Folkmann, D. Rasoloson, G. Seydoux, High efficiency, homology-directed genome editing in *Caenorhabditis elegans* using CRISPR-Cas9 ribonucleoprotein complexes. *Genetics* **201**, 47–54 (2015).

85. L. Timmons, D. L. Court, A. Fire, Ingestion of bacterially expressed dsRNAs can produce specific and potent genetic interference in Caenorhabditis elegans. *Gene* **263**, 103–112 (2001).

86. T. Davies, H. X. Kim, N. Romano Spica, B. J. Lesea-Pringle, J. Dumont, M. Shirasu-Hiza, J. C. Canman, Cell-intrinsic and -extrinsic mechanisms promote cell-type-specific cytokinetic diversity. *eLife* **7**, e36204 (2018).

87. R. S. Kamath, J. Ahringer, Genome-wide RNAi screening in Caenorhabditis elegans. *Methods* **30**, 313–321 (2003).

88. R. S. Kamath, A. G. Fraser, Y. Dong, G. Poulin, R. Durbin, M. Gotta, A. Kanapin, N. le Bot, S. Moreno, M. Sohrmann, D. P. Welchman, P. Zipperlen, J. Ahringer, Systematic functional analysis of the Caenorhabditis elegans genome using RNAi. *Nature* **421**, 231–237 (2003).

89. H. Zhang, A. R. Skop, J. G. White, Src and Wnt signaling regulate dynactin accumulation to the P2-EMS cell border in C. elegans embryos. *J. Cell Sci.* **121**, 155–161 (2008).

90. C. M. Phillips, K. L. McDonald, A. F. Dernburg, in *Meiosis: Volume 2, Cytological Methods*, S. Keeney, Ed. (Humana Press, 2009), pp. 171–195.

91. A. J. MacQueen, C. M. Phillips, N. Bhalla, P. Weiser, A. M. Villeneuve, A. F. Dernburg, Chromosome sites play dual roles to establish homologous synapsis during meiosis in C. elegans. *Cell* **123**, 1037–1050 (2005).

92. C. M. Phillips, C. Wong, N. Bhalla, P. M. Carlton, P. Weiser, P. M. Meneely, A. F. Dernburg, HIM-8 binds to the X chromosome pairing center and mediates chromosome-specific meiotic synapsis. *Cell* **123**, 1051–1063 (2005).

93. A. Gartner, A. J. MacQueen, A. M. Villeneuve, in *Checkpoint Controls and Cancer: Volume 1: Reviews and Model Systems*, A. H. Schönthal, Ed. (Humana Press, 2004), pp. 257–274.

94. M. Han, G. Wei, C. E. McManus, L. W. Hillier, V. Reinke, Isolated C. elegans germ nuclei exhibit distinct genomic profiles of histone modification and gene expression. *BMC Genomics* **20**, 500 (2019).

95. A. Lai, R. Rex, K. L. Cotner, A. Dong, M. Lustig, L. L. Sohn, Mechano-node-pore sensing: A rapid, label-free platform for multi-parameter single-cell viscoelastic measurements. *J. Vis. Exp.*, e64665 (2022).

96. R. W. DeBlois, C. P. Bean, Counting and sizing of submicron particles by the resistive pulse technique. *Rev. Sci. Instrum.* **41**, 909–916 (1970).

97. O. A. Saleh, L. L. Sohn, Correcting off-axis effects in an on-chip resistive-pulse analyzer. *Rev. Sci. Instrum.* **73**, 4396–4398 (2002).
