# Supplementary material for: A cooperative network at the nuclear envelope counteracts LINC-mediated forces during oogenesis in C. elegans
Source: Sci Adv. 2023 Jul 12;9(28):eabn5709. doi: 10.1126/sciadv.abn5709 (PMC10337908; doi:10.1126/sciadv.abn5709)
Supplement: Supplementary file 3 — Data S1 [file sciadv.abn5709_data_s1.zip › abn5709_Data_S1.pdf]

SOURCE DATA

STATISTICAL SOURCE DATA FOR

A cooperative network at the nuclear envelope counteracts LINC-mediated forces during oogenesis in *C. elegans*

Chenshu Liu, Rachel Rex, Zoe Lung, John S. Wang, Fan Wu, Hyung Jun Kim, Liangyu Zhang, Lydia L. Sohn and Abby F. Dernburg
*Corresponding author. chenshu.liu@berkeley.edu; afdernburg@berkeley.edu

TABLE OF CONTENT:

Additional output of statistical analyses for data presented in main figures and Supplementary figures are as following (statistics of interests are highlighted):

**Output of statistical analyses**	**Figure(s)**
Mann-Whitney test for comparing the number of apoptotic nuclei per gonad arm	**Fig. 1F**
One-way ANOVA and post hoc pairwise t-tests for comparing normalized nuclear sizes	**Fig. 1H**
Two-way ANOVA on the mean and relative standard deviation of SUN-1::mRuby intensity;	**Fig. 2B,C**
Two-way ANOVA on the mean speed of SUN-1::mRuby tracks	**Fig. 2E**
Quantification of the rate of nuclear collapse both before or after contact between NE and SC	**Fig. 2I**
Statistics for asymmetry of diplotene LINC distribution, re-binned, t-test for peak intensity value between 170-190°	**Fig. 3B**
One-way ANOVA and post hoc pairwise t-tests for comparing normalized nuclear sizes	**Fig. 3E**
For comparing the percentage of nuclear collapse in each zone, post hoc pair-wise comparison of proportions	**Fig. 4B**
For comparing the percentage of nuclear collapse in each zone, post-hoc pair-wise comparison of proportions	**Fig. 4D**
Welch ANOVA and Games-Howell post hoc multiple comparisons test for comparing wCDI	**Fig. 5F**
Unpaired two-sample two-sided t-test for comparing normalized nuclear radius, nuclear volume (derived), DAPI volume as well as DAPI integrated intensity in control or Auxin treated animals	**Fig. S3C**
For comparing the proportion of nuclei with at least one RAD-51 focus in each zone across groups, post hoc pair wise comparison of proportions	**Fig. S4C**
For comparing the percentage of X-chromosome pairing or complete synapsis	**Fig. S6C**
Two-way ANOVA on the mean and relative standard deviation of ZYG-12::GFP intensity;	**Fig. S7A,B**
One-way ANOVA and post hoc pairwise t-tests for comparing mean intensity of V5 staining per nucleus	**Fig. S12B**
Pairwise Mann-Whitney test for comparing germline apoptosis using CED-1::GFP	**Fig. S13A**
Mann-Whitney test for compairing germline apoptosis using acridine orange	**Fig. S13B**
Statistics for comparing the extent of asymmetry	**Fig. S17B**
Statistics for comparing the extent of asymmetry	**Fig. S17D**
Statistics for comparing nuclear size	**Fig. S18B**

Mann-Whitney test for comparing the number of apoptotic nuclei per gonad arm; related to Fig. 1F:

```
# for lmn-1::AID -/+ Auxin; Hereafter the code with "length" is for computing sample size

> tapply(dat_1$count, dat_1$treatment, mean)
     -Aux      +Aux
 6.894737 16.520000
> tapply(dat_1$count, dat_1$treatment, sd)
    -Aux     +Aux
3.264195 5.470527
> tapply(dat_1$count, dat_1$treatment, length)
-Aux +Aux
  19   25


> wilcox.test(count~treatment, data = dat_1, exact = FALSE, alternative = "two.sided")

        Wilcoxon rank sum test with continuity correction

data:  count by treatment
W = 27.5, p-value = 6.664e-07
alternative hypothesis: true location shift is not equal to 0


# for lmn-1::AID; ced-4 -/+ Auxin

> tapply(dat_2$count, dat_2$treatment, mean)
      -Aux       +Aux
0.00000000 0.07692308
> tapply(dat_2$count, dat_2$treatment, sd)
     -Aux      +Aux
0.0000000 0.2773501
> tapply(dat_2$count, dat_2$treatment, length)
-Aux +Aux
  16   13


> wilcox.test(count~treatment, data = dat_2, exact = FALSE, alternative = "two.sided")

        Wilcoxon rank sum test with continuity correction

data:  count by treatment
W = 96, p-value = 0.2983
alternative hypothesis: true location shift is not equal to 0
```

count	group
3	lmn-1::AID, -Aux
3	lmn-1::AID, -Aux
4	lmn-1::AID, -Aux
8	lmn-1::AID, -Aux
6	lmn-1::AID, -Aux
4	lmn-1::AID, -Aux
8	lmn-1::AID, -Aux
1	lmn-1::AID, -Aux
7	lmn-1::AID, -Aux
10	lmn-1::AID, -Aux
11	lmn-1::AID, -Aux
11	lmn-1::AID, -Aux
12	lmn-1::AID, -Aux
6	lmn-1::AID, -Aux
6	lmn-1::AID, -Aux
12	lmn-1::AID, -Aux
8	lmn-1::AID, -Aux
7	lmn-1::AID, -Aux
4	lmn-1::AID, -Aux
10	lmn-1::AID, +Aux
12	lmn-1::AID, +Aux
14	lmn-1::AID, +Aux
23	lmn-1::AID, +Aux
17	lmn-1::AID, +Aux
13	lmn-1::AID, +Aux
28	lmn-1::AID, +Aux
12	lmn-1::AID, +Aux
18	lmn-1::AID, +Aux
5	lmn-1::AID, +Aux
19	lmn-1::AID, +Aux
15	lmn-1::AID, +Aux
17	lmn-1::AID, +Aux
21	lmn-1::AID, +Aux
21	lmn-1::AID, +Aux
22	lmn-1::AID, +Aux
14	lmn-1::AID, +Aux
9	lmn-1::AID, +Aux
11	lmn-1::AID, +Aux
20	lmn-1::AID, +Aux
15	lmn-1::AID, +Aux
18	lmn-1::AID, +Aux
27	lmn-1::AID, +Aux
14	lmn-1::AID, +Aux
18	lmn-1::AID, +Aux
0	lmn-1::AID; ced-4, -Aux
0	lmn-1::AID; ced-4, -Aux
0	lmn-1::AID; ced-4, -Aux
0	lmn-1::AID; ced-4, -Aux
0	lmn-1::AID; ced-4, -Aux
0	lmn-1::AID; ced-4, -Aux
0	lmn-1::AID; ced-4, -Aux
0	lmn-1::AID; ced-4, -Aux
0	lmn-1::AID; ced-4, -Aux
0	lmn-1::AID; ced-4, -Aux
0	lmn-1::AID; ced-4, -Aux
0	lmn-1::AID; ced-4, -Aux
0	lmn-1::AID; ced-4, -Aux
0	lmn-1::AID; ced-4, -Aux
0	lmn-1::AID; ced-4, -Aux
0	lmn-1::AID; ced-4, -Aux
1	lmn-1::AID;ced-4, +Aux
0	lmn-1::AID;ced-4, +Aux
0	lmn-1::AID;ced-4, +Aux
0	lmn-1::AID;ced-4, +Aux
0	lmn-1::AID;ced-4, +Aux
0	lmn-1::AID;ced-4, +Aux
0	lmn-1::AID;ced-4, +Aux
0	lmn-1::AID;ced-4, +Aux
0	lmn-1::AID;ced-4, +Aux
0	lmn-1::AID;ced-4, +Aux
0	lmn-1::AID;ced-4, +Aux
0	lmn-1::AID;ced-4, +Aux
0	lmn-1::AID;ced-4, +Aux

One-way ANOVA and post hoc pairwise t-tests for comparing normalized nuclear sizes; Related to Fig. 1H:

```
# hereafter the "N" in the code is for computing sample size

 > tapply(dat_norm_2$volume, list(dat_norm_2$group, dat_norm_2$zone), function(x) {
 +   mean_x <- mean(x)
 +   sd_x <- sd(x)
 +   n_x <- length(x)
 +   paste("Mean:", round(mean_x, 2), "SD:", round(sd_x, 2), "N:", n_x)
 + })
                          Late Pachytene              Diplotene
 - Auxin                  "Mean: 1 SD: 0.24 N: 120" "Mean: 1.35 SD: 0.41 N: 111"
 LMN-1 AID                "Mean: 1 SD: 0.22 N: 130" "Mean: 0.42 SD: 0.22 N: 117"
 LMN-1 AID, ced-4         "Mean: 1 SD: 0.2 N: 161"  "Mean: 0.49 SD: 0.32 N: 165"


> pairwise.t.test(data_t$volume, data_t$group,
+                 p.adjust.method = "BH")

        Pairwise comparisons using t tests with pooled SD

data:  data_t$volume and data_t$group

                 - Auxin LMN-1 AID
LMN-1 AID        <2e-16  -
LMN-1 AID, ced-4 <2e-16  0.061

P value adjustment method: BH
```

Two-way ANOVA on the mean and relative standard deviation of SUN-1::mRuby intensity; related to Fig. 2B,C:

```
# Use tapply() to report summary statistics by factor levels
# for mean
> tapply(df_norm$mean, list(df_norm$zone, df_norm$group), function(x) {
+   mean_x <- mean(x)
+   sd_x <- sd(x)
+   n_x <- length(x)
+   paste("Mean:", round(mean_x, 2), "SD:", round(sd_x, 2), "N:", n_x)
+ })
    - Aux                           + Aux
TZ  "Mean: 1 SD: 0.14 N: 30"    "Mean: 1 SD: 0.18 N: 30"
MP  "Mean: 1.95 SD: 0.6 N: 30"  "Mean: 1.05 SD: 0.24 N: 30"
LP  "Mean: 3.54 SD: 0.75 N: 30" "Mean: 1.47 SD: 0.48 N: 30"
Dip "Mean: 3.51 SD: 1.06 N: 30" "Mean: 1.41 SD: 0.45 N: 30"

> # for relative standard deviation
> tapply(df_all$coef_v, list(df_all$zone, df_all$group), function(x) {
+   mean_x <- mean(x)
+   sd_x <- sd(x)
+   n_x <- length(x)
+   paste("Mean:", round(mean_x, 2), "SD:", round(sd_x, 2), "N:", n_x)
+ })
    - Aux                           + Aux
TZ  "Mean: 0.32 SD: 0.09 N: 30" "Mean: 0.34 SD: 0.08 N: 30"
MP  "Mean: 0.13 SD: 0.04 N: 30" "Mean: 0.25 SD: 0.07 N: 30"
LP  "Mean: 0.13 SD: 0.03 N: 30" "Mean: 0.25 SD: 0.08 N: 30"
Dip "Mean: 0.14 SD: 0.05 N: 30" "Mean: 0.29 SD: 0.09 N: 30"



> res.aov3 <- aov(mean ~ zone * group, data = df_norm)
> summary(res.aov3)
             Df Sum Sq Mean Sq F value Pr(>F)
zone          3  98.51   32.84  101.35 <2e-16 ***
group         1  96.22   96.22  296.99 <2e-16 ***
zone:group    3  46.10   15.37   47.44 <2e-16 ***
Residuals   232  75.16    0.32
---
Signif. codes:  0 ‘***’ 0.001 ‘**’ 0.01 ‘*’ 0.05 ‘.’ 0.1 ‘ ’ 1
> res.aov4 <- aov(coef_v ~ zone * group, data = df_norm)
> summary(res.aov4)
             Df Sum Sq Mean Sq F value   Pr(>F)
zone          3 0.7650  0.2550  55.452  < 2e-16 ***
group         1 0.5890  0.5890 128.098  < 2e-16 ***
zone:group    3 0.1252  0.0417   9.073 1.05e-05 ***
Residuals   232 1.0668  0.0046
---
Signif. codes:  0 ‘***’ 0.001 ‘**’ 0.01 ‘*’ 0.05 ‘.’ 0.1 ‘ ’ 1
```

Two-way ANOVA on the mean speed of SUN-1::mRuby tracks; Related to Fig. 2E:

exp == 1 means ctrl, exp == 2 means + auxin

“1, 2, 3” under each “exp” are “TZ, MP, LP” respectively

```
> dat2_ctrl <- subset(dat2, dat2$exp == 1)
> tapply(dat2_ctrl$Track.Speed.Mean.Reference.Frame, dat2_ctrl$stage, mean)
       1        2        3 
60.35049 42.19735 33.55217 
> tapply(dat2_ctrl$Track.Speed.Mean.Reference.Frame, dat2_ctrl$stage, sd)
       1        2        3 
24.31436 19.79250 18.37615 
> tapply(dat2_ctrl$Track.Speed.Mean.Reference.Frame, dat2_ctrl$stage, length)
   1    2    3 
1132  620  306 

> dat2_aux <- subset(dat2, dat2$exp == 2)
> tapply(dat2_aux$Track.Speed.Mean.Reference.Frame, dat2_aux$stage, mean)
       1        2        3 
53.38761 44.83548 41.41716 
> tapply(dat2_aux$Track.Speed.Mean.Reference.Frame, dat2_aux$stage, sd)
       1        2        3 
23.25021 18.42933 18.53680 
> tapply(dat2_aux$Track.Speed.Mean.Reference.Frame, dat2_aux$stage, length)
   1    2    3 
 859 1657 1675 


> # Two-way ANOVA with interaction effect
> res.aov3 <- aov(Track.Speed.Mean.Reference.Frame ~ stage + exp + stage:exp, data = dat
2)
> summary(res.aov3)
              Df  Sum Sq Mean Sq F value Pr(>F)    
stage          2  323545  161773 386.127 <2e-16 ***
exp            1      26      26   0.061  0.804    
stage:exp      2   42797   21399  51.075 <2e-16 ***
Residuals   6243 2615579     419                   
---
Signif. codes:  0 ‘***’ 0.001 ‘**’ 0.01 ‘*’ 0.05 ‘.’ 0.1 ‘ ’ 1
```

Quantification of the rate of nuclear collapse both before or after contact between NE and SC; Related to Fig. 2I:

```
# “pre” means before SC/NE contact, “post” means after SC/NE contact

> dat_pre <- subset(dat_v, dat_v$time == "pre")
> wilcox.test(speed~group, data = dat_pre, exact = FALSE, alternative = "two.sided")

        Wilcoxon rank sum test with continuity correction

data:  speed by group
W = 100, p-value = 0.0001827
alternative hypothesis: true location shift is not equal to 0

> dat_post <- subset(dat_v, dat_v$time == "post")
> wilcox.test(speed~group, data = dat_post, exact = FALSE, alternative = "two.sided")

        Wilcoxon rank sum test with continuity correction

data:  speed by group
W = 59, p-value = 0.5204
alternative hypothesis: true location shift is not equal to 0


> tapply(dat_pre$speed, dat_pre$group, mean)
         SC           NE 
 0.00246972 -0.00999650 
> tapply(dat_pre$speed, dat_pre$group, sd)
         SC           NE 
0.003793290 0.005103751 
> tapply(dat_pre$speed, dat_pre$group, length)
SC NE 
10 10 


> tapply(dat_post$speed, dat_post$group, mean)
        SC         NE 
-0.0102081 -0.0111930 
> tapply(dat_post$speed, dat_post$group, sd)
         SC           NE 
0.004974953 0.005258206 
> tapply(dat_post$speed, dat_post$group, length)
SC NE 
10 10 
```

group	time	speed
SC	pre	0.003053
SC	pre	0.000561
SC	pre	0.000228
SC	pre	0.008482
SC	pre	0.00744
SC	pre	-0.000845
SC	pre	-0.000108
SC	pre	-0.001549
SC	pre	0.00709
SC	pre	0.0003444
NE	pre	-0.01982
NE	pre	-0.009296
NE	pre	-0.01111
NE	pre	-0.006255
NE	pre	-0.01813
NE	pre	-0.004558
NE	pre	-0.005753
NE	pre	-0.008799
NE	pre	-0.008077
NE	pre	-0.008167

SC	post	-0.01222
SC	post	-0.003432
SC	post	-0.0119
SC	post	-0.01435
SC	post	-0.0126
SC	post	-0.01549
SC	post	-0.005946
SC	post	-0.000893
SC	post	-0.0115
SC	post	-0.01375
NE	post	-0.01626
NE	post	-0.004331
NE	post	-0.0108
NE	post	-0.01808
NE	post	-0.01503
NE	post	-0.01438
NE	post	-0.003878
NE	post	-0.004331
NE	post	-0.01247
NE	post	-0.01237

Statistics for asymmetry of diplotene LINC distribution, re-binned, t-test for peak intensity value between 170-190°; related to Fig. 3B:

```
Sample size: # of nuclei
L4440 -Auxin, n = 51; L4440 +Auxin, n = 41;
dnc-1 -Auxin, n = 47; dnc-1 +Auxin, n = 52;
dlc-1 -Auxin, n = 55; dlc-1 +Auxin, n = 53.
```

L4440 -/+Aux (1 being -Aux, 2 being + Aux):

```
> df_t <- rbind(df_t1,df_t2)
> df_t$id <- factor(df_t$id)
> res.ftest <- var.test(Y ~ id, data = df_t)
> res.ftest
F test to compare two variances
data:  Y by id
F = 0.056958, num df = 420, denom df = 295, p-value < 2.2e-16
alternative hypothesis: true ratio of variances is not equal to 1
95 percent confidence interval:
 0.04603585 0.07019336
sample estimates:
ratio of variances
         0.0569576
>
> t.test(Y~id, data = df_t, alternative = "two.sided", var.equal = TRUE)
Two Sample t-test
data:  Y by id
t = -27.112, df = 715, p-value < 2.2e-16
alternative hypothesis: true difference in means is not equal to 0
95 percent confidence interval:
 -0.8531666 -0.7379485
sample estimates:
mean in group 1 mean in group 2
       1.197693        1.993250
> t.test(Y~id, data = df_t, alternative = "two.sided", var.equal = FALSE)
Welch Two Sample t-test
data:  Y by id
t = -23.171, df = 318.74, p-value < 2.2e-16
alternative hypothesis: true difference in means is not equal to 0
95 percent confidence interval:
 -0.8631076 -0.7280075
sample estimates:
mean in group 1 mean in group 2
       1.197693        1.993250

100*(1.993250-1.197693)/1.197693 = 66.424117 percent difference;
```

(below, left: L4440 -Auxin; right: L4440 +Auxin)

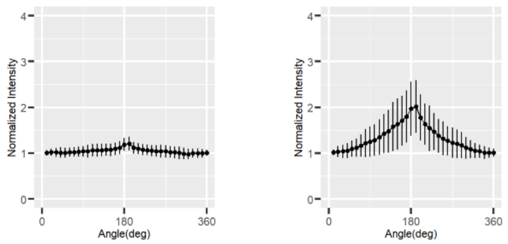

dnc-1 -/+ Aux (1 being -Aux, 2 being +Aux):

```
> df_t <- rbind(df_t1,df_t2)
> df_t$id <- factor(df_t$id)
> res.ftest <- var.test(Y ~ id, data = df_t)
> res.ftest
F test to compare two variances
```

```
data:  Y by id
F = 0.84728, num df = 326, denom df = 411, p-value = 0.1166
alternative hypothesis: true ratio of variances is not equal to 1
95 percent confidence interval:
 0.6904682 1.0422827
sample estimates:
ratio of variances
         0.8472795
>
> t.test(Y~id, data = df_t, alternative = "two.sided", var.equal = TRUE)
Two Sample t-test
data:  Y by id
t = 1.1265, df = 737, p-value = 0.2603
alternative hypothesis: true difference in means is not equal to 0
95 percent confidence interval:
 -0.01169140  0.04317295
sample estimates:
mean in group 1 mean in group 2
       1.253348        1.237607
100*(1.237607-1.253348)/1.253348 = -1.255916 percent different;

> t.test(Y~id, data = df_t, alternative = "two.sided", var.equal = FALSE)
Welch Two Sample t-test
data:  Y by id
t = 1.1373, df = 721, p-value = 0.2558
alternative hypothesis: true difference in means is not equal to 0
95 percent confidence interval:
 -0.01143242  0.04291397
sample estimates:
mean in group 1 mean in group 2
       1.253348        1.237607
```

(below, left: dnc-1 -Auxin; right: dnc-1 +Auxin)

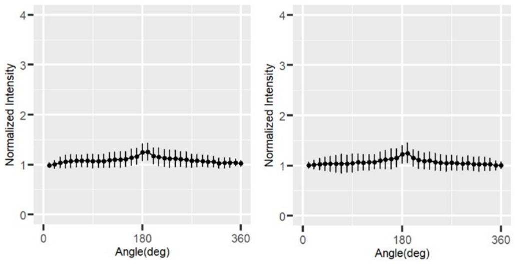

dlc-1 -/+ Aux (1 being -Aux, 2 being +Aux):

```
> df_t <- rbind(df_t1,df_t2)
> df_t$id <- factor(df_t$id)
> res.ftest <- var.test(Y ~ id, data = df_t)
> res.ftest
F test to compare two variances
data:  Y by id
F = 0.71667, num df = 465, denom df = 436, p-value = 0.0004144
alternative hypothesis: true ratio of variances is not equal to 1
95 percent confidence interval:
 0.5954561 0.8620879
sample estimates:
ratio of variances
         0.7166744
>
> t.test(Y~id, data = df_t, alternative = "two.sided", var.equal = TRUE)
Two Sample t-test
data:  Y by id
t = -4.748, df = 901, p-value = 2.388e-06
alternative hypothesis: true difference in means is not equal to 0
95 percent confidence interval:
 -0.06275017 -0.02604620
sample estimates:
```

```
mean in group 1 mean in group 2 
       1.162345        1.206744 
> t.test(Y~id, data = df_t, alternative = "two.sided", var.equal = FALSE)
Welch Two Sample t-test
data:  Y by id
t = -4.7229, df = 856.32, p-value = 2.716e-06
alternative hypothesis: true difference in means is not equal to 0
95 percent confidence interval:
 -0.06284911 -0.02594727
sample estimates:
mean in group 1 mean in group 2 
       1.162345        1.206744 

100*(1.206744-1.162345)/1.152345 = 3.852926 percent different;
```

(below, left: dlc-1 -Auxin; right: dlc-1 +Auxin)

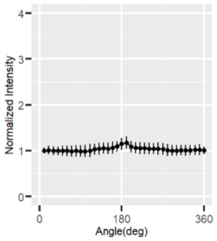

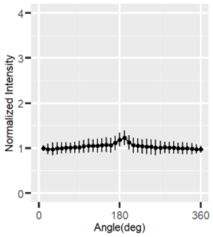

One-way ANOVA and post hoc pairwise t-tests for comparing normalized nuclear sizes; related to Fig. 3E:

```
> # Use tapply() to report summary statistics by factor levels
> tapply(dat_norm_all_1$volume, list(dat_norm_all_1$group, dat_norm_all_1$zone), function
(x) {
+   mean_x <- mean(x)
+   sd_x <- sd(x)
+   n_x <- length(x)
+   paste("Mean:", round(mean_x, 2), "SD:", round(sd_x, 2), "N:", n_x)
+ })
                       Late Pachytene             Diplotene
L4440                  "Mean: 1 SD: 0.18 N: 110" "Mean: 1.68 SD: 0.46 N: 88"
L4440, LMN-1 AID       "Mean: 1 SD: 0.25 N: 100" "Mean: 0.48 SD: 0.13 N: 131"
dnc-1 RNAi             "Mean: 1 SD: 0.27 N: 142" "Mean: 1.59 SD: 0.47 N: 81"
dnc-1 RNAi, LMN-1 AID "Mean: 1 SD: 0.19 N: 82"  "Mean: 1.52 SD: 0.41 N: 100"
lis-1 RNAi             "Mean: 1 SD: 0.26 N: 139" "Mean: 1.79 SD: 0.56 N: 103"
lis-1 RNAi, LMN-1 AID "Mean: 1 SD: 0.21 N: 112" "Mean: 1.44 SD: 0.51 N: 140"
dlc-1 RNAi             "Mean: 1 SD: 0.25 N: 113" "Mean: 1.6 SD: 0.51 N: 87"
dlc-1 RNAi, LMN-1 AID "Mean: 1 SD: 0.24 N: 98"  "Mean: 1.55 SD: 0.39 N: 108"
- Auxin                "Mean: 1 SD: 0.24 N: 120" "Mean: 1.35 SD: 0.41 N: 111"
LMN-1 AID              "Mean: 1 SD: 0.22 N: 130" "Mean: 0.42 SD: 0.22 N: 117"
SUN-1 AID              "Mean: 1 SD: 0.28 N: 126" "Mean: 1.45 SD: 0.52 N: 136"
LMN-1, SUN-1 AID       "Mean: 1 SD: 0.25 N: 160" "Mean: 1.47 SD: 0.53 N: 115"
ZYG-12 AID             "Mean: 1 SD: 0.27 N: 121" "Mean: 1.04 SD: 0.4 N: 116"
LMN-1, ZYG-12 AID      "Mean: 1 SD: 0.39 N: 124" "Mean: 1.09 SD: 0.42 N: 105"
emr-1                  "Mean: 1 SD: 0.19 N: 106" "Mean: 1.43 SD: 0.33 N: 73"
emr-1, LMN-1 AID       "Mean: 1 SD: 0.2 N: 87"   "Mean: 0.45 SD: 0.09 N: 99"
LEM-2 AID              "Mean: 1 SD: 0.17 N: 122" "Mean: 1.6 SD: 0.34 N: 97"
LEM-2, LMN-1 AID       "Mean: 1 SD: 0.18 N: 128" "Mean: 0.42 SD: 0.16 N: 156"
ieDf2_het_Ctrl_RNAi    "Mean: 1 SD: 0.23 N: 153" "Mean: 1.45 SD: 0.38 N: 99"
ieDf2_het_lmn-1_RNAi   "Mean: 1 SD: 0.23 N: 139" "Mean: 0.47 SD: 0.19 N: 129"
ieDf2_Ctrl_RNAi        "Mean: 1 SD: 0.21 N: 163" "Mean: 1.71 SD: 0.45 N: 83"
ieDf2_lmn-1_RNAi       "Mean: 1 SD: 0.23 N: 126" "Mean: 0.47 SD: 0.19 N: 101"
```

```
> data_t1 = subset(dat_norm_1, dat_norm_1$zone == "Diplotene")
> res.aov <- aov(volume~group, data = data_t1)
> summary(res.aov)
              Df Sum Sq Mean Sq F value Pr(>F)
group         17  447.5  26.326   162.9 <2e-16 ***
Residuals   1945  314.2   0.162
---
Signif. codes:  0 ‘***’ 0.001 ‘**’ 0.01 ‘*’ 0.05 ‘.’ 0.1 ‘ ’ 1
> pairwise.t.test(data_t1$volume, data_t1$group,
+                 p.adjust.method = "BH")
Pairwise comparisons using t tests with pooled SD
data:  data_t1$volume and data_t1$group
                      - Auxin LMN-1 AID SUN-1 AID LMN-1, SUN-1 AID ZYG-12 AID LMN-1, ZYG-12 AID L4440
LMN-1 AID             < 2e-16 -         -         -                -          -                 -
SUN-1 AID             0.05963 < 2e-16   -         -                -          -                 -
LMN-1, SUN-1 AID      0.03323 < 2e-16   0.76377   -                -          -                 -
ZYG-12 AID            2.2e-08 < 2e-16   3.3e-15   3.1e-15          -          -                 -
LMN-1, ZYG-12 AID     5.2e-06 < 2e-16   1.6e-11   1.1e-11          0.40562    -                 -
L4440                 8.4e-09 < 2e-16   3.3e-05   0.00022          < 2e-16    < 2e-16           -
L4440, LMN-1 AID      < 2e-16 0.30272   < 2e-16   < 2e-16          < 2e-16    < 2e-16           < 2e-16
dnc-1 RNAi            4.0e-05 < 2e-16   0.01386   0.03696          < 2e-16    < 2e-16           0.18167
dnc-1 RNAi, LMN-1 AID 0.00195 < 2e-16   0.18419   0.32447          < 2e-16    3.0e-14           0.00969
lis-1 RNAi            2.5e-15 < 2e-16   1.9e-10   6.3e-09          < 2e-16    < 2e-16           0.08788
lis-1 RNAi, LMN-1 AID 0.09022 < 2e-16   0.85319   0.62489          1.1e-14    4.7e-11           1.3e-05
dlc-1 RNAi            1.7e-05 < 2e-16   0.00870   0.02535          < 2e-16    < 2e-16           0.20281
dlc-1 RNAi, LMN-1 AID 0.00035 < 2e-16   0.07425   0.16354          < 2e-16    4.0e-16           0.02441
emr-1                 0.20498 < 2e-16   0.76336   0.58174          2.5e-10    6.3e-08           0.00010
emr-1, LMN-1 AID      < 2e-16 0.63772   < 2e-16   < 2e-16          < 2e-16    < 2e-16           < 2e-16
LEM-2 AID             9.8e-06 < 2e-16   0.00685   0.02142          < 2e-16    < 2e-16           0.18975
LEM-2, LMN-1 AID      < 2e-16 0.97147   < 2e-16   < 2e-16          < 2e-16    < 2e-16           < 2e-16
                      L4440, LMN-1 AID dnc-1 RNAi dnc-1 RNAi, LMN-1 AID lis-1 RNAi lis-1 RNAi, LMN-1 AID dlc-1
RNAi
LMN-1 AID             -                -          -                     -          -                     -
SUN-1 AID             -                -          -                     -          -                     -
LMN-1, SUN-1 AID      -                -          -                     -          -                     -
ZYG-12 AID            -                -          -                     -          -                     -
LMN-1, ZYG-12 AID     -                -          -                     -          -                     -
L4440                 -                -          -                     -          -                     -
L4440, LMN-1 AID      -                -          -                     -          -                     -
dnc-1 RNAi            < 2e-16          -          -                     -          -                     -
dnc-1 RNAi, LMN-1 AID < 2e-16          0.28264    -                     -          -                     -
lis-1 RNAi            < 2e-16          0.00166    4.9e-06               -          -                     -
lis-1 RNAi, LMN-1 AID < 2e-16          0.00790    0.12534               3.9e-11    -                     -
dlc-1 RNAi            < 2e-16          0.93586    0.23208               0.00187    0.00468               -
dlc-1 RNAi, LMN-1 AID < 2e-16          0.46564    0.72562               2.0e-05    0.04520               0.39882
emr-1                 < 2e-16          0.01473    0.14955               8.9e-09    0.89237               0.01005
emr-1, LMN-1 AID      0.63772          < 2e-16    < 2e-16               < 2e-16    < 2e-16               < 2e-16
LEM-2 AID             < 2e-16          0.93586    0.22034               0.00135    0.00346               0.99743
LEM-2, LMN-1 AID      0.24866          < 2e-16    < 2e-16               < 2e-16    < 2e-16               < 2e-16
                      dlc-1 RNAi, LMN-1 AID emr-1   emr-1, LMN-1 AID LEM-2 AID
LMN-1 AID             -                     -       -                -
SUN-1 AID             -                     -       -                -
LMN-1, SUN-1 AID      -                     -       -                -
ZYG-12 AID            -                     -       -                -
LMN-1, ZYG-12 AID     -                     -       -                -
L4440                 -                     -       -                -
L4440, LMN-1 AID      -                     -       -                -
dnc-1 RNAi            -                     -       -                -
dnc-1 RNAi, LMN-1 AID -                     -       -                -
lis-1 RNAi            -                     -       -                -
lis-1 RNAi, LMN-1 AID -                     -       -                -
dlc-1 RNAi            -                     -       -                -
dlc-1 RNAi, LMN-1 AID -                     -       -                -
emr-1                 0.06691               -       -                -
emr-1, LMN-1 AID      < 2e-16               < 2e-16 -                -
LEM-2 AID             0.38777               0.00856 < 2e-16          -
LEM-2, LMN-1 AID      < 2e-16               < 2e-16 0.59395          < 2e-16
P value adjustment method: BH
```

```
> data_t = subset(dat_norm, dat_norm$zone == "Diplotene")
> res.aov <- aov(volume~group, data = data_t)
> summary(res.aov)
             Df Sum Sq Mean Sq F value Pr(>F)
group         3 126.33   42.11   440.4 <2e-16 ***
Residuals   408  39.01    0.10
---
Signif. codes:  0 ‘***’ 0.001 ‘**’ 0.01 ‘*’ 0.05 ‘.’ 0.1 ‘ ’ 1

> pairwise.t.test(data_t$volume, data_t$group,
+                 p.adjust.method = "BH")

        Pairwise comparisons using t tests with pooled SD

data:  data_t$volume and data_t$group

                     ieDf2_het_Ctrl_RNAi ieDf2_het_lmn-1_RNAi ieDf2_Ctrl_RNAi
ieDf2_het_lmn-1_RNAi < 2e-16             -                    -
ieDf2_Ctrl_RNAi      3.8e-08             < 2e-16              -
ieDf2_lmn-1_RNAi     < 2e-16             0.96                 < 2e-16

P value adjustment method: BH
```

For comparing the percentage of nuclear collapse in each zone, post-hoc pair-wise comparison of proportions; related to Fig. 4B:

```
#samp-1 (Fig. 4B) zone 1~6 (sequential sections below); "1" ~ "5" in each section are Control RNAi - Aux, Control RNAi + Aux, samp-1(RNAi) - Aux, samp-1(RNAi) + Aux, sun-1::AID and samp-1(RNAi) + Aux

> prop.test(x=c(5,3,4, 16, 7),n=c(281, 299, 261, 259,  349))
5-sample test for equality of proportions without continuity correction
data:  c(5, 3, 4, 16, 7) out of c(281, 299, 261, 259, 349)
X-squared = 19.676, df = 4, p-value = 0.0005787
alternative hypothesis: two.sided
sample estimates:
    prop 1     prop 2     prop 3     prop 4     prop 5
0.01779359 0.01003344 0.01532567 0.06177606 0.02005731
> pairwise.prop.test(x=c(5,3,4, 16, 7),n=c(281, 299, 261, 259, 349),p.adjust.method = "BH")
Pairwise comparisons using Pairwise comparison of proportions
data:  c(5, 3, 4, 16, 7) out of c(281, 299, 261, 259, 349)
  1     2     3     4
2 1.000 -     -     -
3 1.000 1.000 -     -
4 0.039 0.018 0.039 -
5 1.000 0.953 1.000 0.039
P value adjustment method: BH


> prop.test(x=c(4, 1, 3, 63, 10),n=c(316, 306, 379, 305, 433))
5-sample test for equality of proportions without continuity correction
data:  c(4, 1, 3, 63, 10) out of c(316, 306, 379, 305, 433)
X-squared = 215.02, df = 4, p-value < 2.2e-16
alternative hypothesis: two.sided
sample estimates:
     prop 1      prop 2      prop 3      prop 4      prop 5
0.012658228 0.003267974 0.007915567 0.206557377 0.023094688
> pairwise.prop.test(x=c(4, 1, 3, 63, 10),n=c(316, 306, 379, 305, 433),p.adjust.method = "BH")
Pairwise comparisons using Pairwise comparison of proportions
data:  c(4, 1, 3, 63, 10) out of c(316, 306, 379, 305, 433)
  1       2       3       4
2 0.55    -       -       -
3 0.81    0.81    -       -
4 4.8e-14 2.3e-15 < 2e-16 -
5 0.55    0.12    0.25    2.3e-15
P value adjustment method: BH


> prop.test(x=c(6,64, 20, 175,15),n=c(405, 389, 459, 365, 429))
5-sample test for equality of proportions without continuity correction
data:  c(6, 64, 20, 175, 15) out of c(405, 389, 459, 365, 429)
X-squared = 487.98, df = 4, p-value < 2.2e-16
alternative hypothesis: two.sided
sample estimates:
    prop 1     prop 2     prop 3     prop 4     prop 5
0.01481481 0.16452442 0.04357298 0.47945205 0.03496503
> pairwise.prop.test(x=c(6,64, 20, 175,15),n=c(405, 389, 459, 365, 429),p.adjust.method = "BH")
Pairwise comparisons using Pairwise comparison of proportions
data:  c(6, 64, 20, 175, 15) out of c(405, 389, 459, 365, 429)
  1       2       3       4
2 5.2e-13 -       -       -
3 0.029   1.2e-08 -       -
4 < 2e-16 < 2e-16 < 2e-16 -
5 0.113   1.3e-09 0.627   < 2e-16
P value adjustment method: BH



> prop.test(x=c(12, 50, 10, 82, 10),n=c(326, 287, 407, 195, 354))
5-sample test for equality of proportions without continuity correction
```

```
data:  c(12, 50, 10, 82, 10) out of c(326, 287, 407, 195, 354)
X-squared = 288.68, df = 4, p-value < 2.2e-16
alternative hypothesis: two.sided
sample estimates:
    prop 1     prop 2     prop 3     prop 4     prop 5 
0.03680982 0.17421603 0.02457002 0.42051282 0.02824859 
> pairwise.prop.test(x=c(12, 50, 10, 82, 10),n=c(326, 287, 407, 195, 354),p.adjust.method
= "BH")
Pairwise comparisons using Pairwise comparison of proportions
data:  c(12, 50, 10, 82, 10) out of c(326, 287, 407, 195, 354)
  1       2       3       4      
2 5.6e-08 -       -       -      
3 0.57    3.2e-11 -       -      
4 < 2e-16 8.3e-09 < 2e-16 -      
5 0.75    1.3e-09 0.93    < 2e-16
P value adjustment method: BH


> prop.test(x=c(0, 108, 3, 151, 8),n=c(50, 147, 145, 162, 243))
5-sample test for equality of proportions without continuity correction
data:  c(0, 108, 3, 151, 8) out of c(50, 147, 145, 162, 243)
X-squared = 532.18, df = 4, p-value < 2.2e-16
alternative hypothesis: two.sided
sample estimates:
    prop 1     prop 2     prop 3     prop 4     prop 5 
0.00000000 0.73469388 0.02068966 0.93209877 0.03292181 
> pairwise.prop.test(x=c(0, 108, 3, 151, 8),n=c(50, 147, 145, 162, 243),p.adjust.method =
"BH")
Pairwise comparisons using Pairwise comparison of proportions
data:  c(0, 108, 3, 151, 8) out of c(50, 147, 145, 162, 243)
  1       2       3       4      
2 < 2e-16 -       -       -      
3 0.72    < 2e-16 -       -      
4 < 2e-16 7.6e-06 < 2e-16 -      
5 0.51    < 2e-16 0.72    < 2e-16
P value adjustment method: BH


> prop.test(x=c(0, 91, 0, 103, 6),n=c(18, 92, 19, 106, 132))
5-sample test for equality of proportions without continuity correction
data:  c(0, 91, 0, 103, 6) out of c(18, 92, 19, 106, 132)
X-squared = 328.16, df = 4, p-value < 2.2e-16
alternative hypothesis: two.sided
sample estimates:
    prop 1     prop 2     prop 3     prop 4     prop 5 
0.00000000 0.98913043 0.00000000 0.97169811 0.04545455 
> pairwise.prop.test(x=c(0, 91, 0, 103, 6),n=c(18, 92, 19, 106, 132),p.adjust.method =
"BH")
Pairwise comparisons using Pairwise comparison of proportions
data:  c(0, 91, 0, 103, 6) out of c(18, 92, 19, 106, 132)
  1      2      3      4     
2 <2e-16 -      -      -     
3 -      <2e-16 -      -     
4 <2e-16 0.78   <2e-16 -     
5 0.78   <2e-16 0.78   <2e-16
P value adjustment method: BH
```

For comparing the percentage of nuclear collapse in each zone, post hoc pair-wise comparison of proportions; related to Fig. 4D:

```
#lem-2 (Fig. 4D), zone 1~6 (sequential sections below); “1” ~ “5” in each section are
Control RNAi - Aux, Control RNAi + Aux, lem-2(RNAi) - Aux, lem-2(RNAi) + Aux, sun-1::AID
and lem-2(RNAi) + Aux
> prop.test(x=c(5,4,92,114,83),n=c(258,287,244,188,201),correct = TRUE)
5-sample test for equality of proportions without continuity correction
data:  c(5, 4, 92, 114, 83) out of c(258, 287, 244, 188, 201)
X-squared = 332.62, df = 4, p-value < 2.2e-16
alternative hypothesis: two.sided
sample estimates:
    prop 1     prop 2     prop 3     prop 4     prop 5
0.01937984 0.01393728 0.37704918 0.60638298 0.41293532
> pairwise.prop.test(x=c(5,4,92,114,83),n=c(258,287,244,188,201),p.adjust.method = "BH")
Pairwise comparisons using Pairwise comparison of proportions
data:  c(5, 4, 92, 114, 83) out of c(258, 287, 244, 188, 201)
  1       2       3       4
2 0.87194 -       -       -
3 < 2e-16 < 2e-16 -       -
4 < 2e-16 < 2e-16 5.1e-06 -
5 < 2e-16 < 2e-16 0.55608 0.00026
P value adjustment method: BH

> prop.test(x=c(9,5,42,104,14),n=c(342, 320, 376,  208, 241))
5-sample test for equality of proportions without continuity correction
data:  c(9, 5, 42, 104, 14) out of c(342, 320, 376, 208, 241)
X-squared = 362.55, df = 4, p-value < 2.2e-16
alternative hypothesis: two.sided
sample estimates:
    prop 1     prop 2     prop 3     prop 4     prop 5
0.02631579 0.01562500 0.11170213 0.50000000 0.05809129
> pairwise.prop.test(x=c(9,5,42,104,14),n=c(342, 320,376, 208,241),p.adjust.method =
"BH")
Pairwise comparisons using Pairwise comparison of proportions
data:  c(9, 5, 42, 104, 14) out of c(342, 320, 376, 208, 241)
  1       2       3       4
2 0.493   -       -       -
3 2.8e-05 2.1e-06 -       -
4 < 2e-16 < 2e-16 < 2e-16 -
5 0.094   0.017   0.043   < 2e-16
P value adjustment method: BH


> prop.test(x=c(6,82,26, 163,9),n=c(448,373,439,235, 231))
5-sample test for equality of proportions without continuity correction
data:  c(6, 82, 26, 163, 9) out of c(448, 373, 439, 235, 231)
X-squared = 619.68, df = 4, p-value < 2.2e-16
alternative hypothesis: two.sided
sample estimates:
    prop 1     prop 2     prop 3     prop 4     prop 5
0.01339286 0.21983914 0.05922551 0.69361702 0.03896104
> pairwise.prop.test(x=c(6,82,26,163,9),n=c(448, 373, 439, 235,231),p.adjust.method =
"BH")
Pairwise comparisons using Pairwise comparison of proportions
data:  c(6, 82, 26, 163, 9) out of c(448, 373, 439, 235, 231)
  1       2       3       4
2 < 2e-16 -       -       -
3 0.00063 6.3e-11 -       -
4 < 2e-16 < 2e-16 < 2e-16 -
5 0.06800 4.5e-09 0.34835 < 2e-16
P value adjustment method: BH


> prop.test(x=c(19,  91,  25,  159, 13),n=c(437, 292,318,189,127))
5-sample test for equality of proportions without continuity correction
data:  c(19, 91, 25, 159, 13) out of c(437, 292, 318, 189, 127)
X-squared = 556.4, df = 4, p-value < 2.2e-16
alternative hypothesis: two.sided
sample estimates:
```

```
    prop 1     prop 2     prop 3     prop 4     prop 5
0.04347826 0.31164384 0.07861635 0.84126984 0.10236220
> pairwise.prop.test(x=c(19,91,25,159,13),n=c(437,292,318,189,127),p.adjust.method =
"BH")
Pairwise comparisons using Pairwise comparison of proportions
data:  c(19, 91, 25, 159, 13) out of c(437, 292, 318, 189, 127)
  1       2       3       4
2 < 2e-16 -       -       -
3 0.067   8.5e-13 -       -
4 < 2e-16 < 2e-16 < 2e-16 -
5 0.026   1.3e-05 0.534   < 2e-16
P value adjustment method: BH

> prop.test(x=c(12,154,8,66,13),n=c(198, 197, 94, 76, 87))
5-sample test for equality of proportions without continuity correction
data:  c(12, 154, 8, 66, 13) out of c(198, 197, 94, 76, 87)
X-squared = 349.02, df = 4, p-value < 2.2e-16
alternative hypothesis: two.sided
sample estimates:
    prop 1     prop 2     prop 3     prop 4     prop 5
0.06060606 0.78172589 0.08510638 0.86842105 0.14942529
> pairwise.prop.test(x=c(12,154,8,66,13),n=c(198,197,94, 76, 87),p.adjust.method = "BH")
Pairwise comparisons using Pairwise comparison of proportions
data:  c(12, 154, 8, 66, 13) out of c(198, 197, 94, 76, 87)
  1      2      3      4
2 <2e-16 -      -      -
3 0.599  <2e-16 -      -
4 <2e-16 0.183  <2e-16 -
5 0.038  <2e-16 0.293  <2e-16
P value adjustment method: BH


> prop.test(x=c(1,80, 5,41,2),n=c(24, 86,  26, 43, 39))
5-sample test for equality of proportions without continuity correction
data:  c(1, 80, 5, 41, 2) out of c(24, 86, 26, 43, 39)
X-squared = 158.47, df = 4, p-value < 2.2e-16
alternative hypothesis: two.sided
sample estimates:
    prop 1     prop 2     prop 3     prop 4     prop 5
0.04166667 0.93023256 0.19230769 0.95348837 0.05128205
> pairwise.prop.test(x=c(1,80,5,41,2),n=c(24,86,26, 43, 39),p.adjust.method = "BH")
Pairwise comparisons using Pairwise comparison of proportions
data:  c(1, 80, 5, 41, 2) out of c(24, 86, 26, 43, 39)
  1       2       3       4
2 < 2e-16 -       -       -
3 0.29    2.4e-13 -       -
4 1.9e-12 1.00    7.5e-10 -
5 1.00    < 2e-16 0.24    6.3e-15
P value adjustment method: BH
```

Welch ANOVA and Games-Howell post hoc multiple comparisons test for comparing wCDI, related to Fig. 5F:

"1" "2" "3" are Control RNAi - Aux, Control RNAi + Aux, *samp-1(RNAi)* + Aux, respectively

```
> tapply(dat$wCDI, dat$group, mean)
        1         2         3 
0.4883792 0.5507192 0.5486340 
> tapply(dat$wCDI, dat$group, sd)
         1          2          3 
0.05560139 0.06995033 0.09903256 
> tapply(dat$wCDI, dat$group, length)
 1  2  3 
53 53 71 


> #perform Welch's ANOVA
> oneway.test(wCDI ~ group, data = dat, var.equal = FALSE)

        One-way analysis of means (not assuming equal variances)

data:  wCDI and group
F = 16.553, num df = 2.00, denom df = 114.45, p-value = 4.85e-07


> games_howell_test(dat, wCDI ~ group)
# A tibble: 3 x 8
  .y.   group1 group2 estimate conf.low conf.high      p.adj p.adj.signif
* <chr> <chr>  <chr>     <dbl>    <dbl>     <dbl>      <dbl> <chr>       
1 wCDI  1      2       0.0623    0.0331    0.0915 0.00000532 ****        
2 wCDI  1      3       0.0603    0.0270    0.0935 0.000107   ***         
3 wCDI  2      3      -0.00209  -0.0381    0.0339 0.99       ns          

> install.packages('pwr')
> library(pwr)
> # power analysis for sample 1 and 2
> pwr::pwr.t2n.test(n1 = c(53),
+                   n2 = c(53),
+                   sig.level = 0.05,
+                   alternative = "two.sided",
+                   power = NULL,
+                   d=(0.4883792-0.5507192)/sqrt((0.05560139^2 + 0.06995033^2)/2))

     t test power calculation 

             n1 = 53
             n2 = 53
              d = 0.9866343
      sig.level = 0.05
          power = 0.9989356
    alternative = two.sided
> # power analysis for sample 1 and 3
> pwr::pwr.t2n.test(n1 = c(53),
+                   n2 = c(71),
+                   sig.level = 0.05,
+                   alternative = "two.sided",
+                   power = NULL,
+                   d=(0.4883792-0.5486340)/sqrt((0.05560139^2 + 0.09903256^2)/2))

     t test power calculation 

             n1 = 53
             n2 = 71
              d = 0.7502902
      sig.level = 0.05
          power = 0.9838421
    alternative = two.sided
```

Unpaired two-sample two-sided t-test for comparing normalized nuclear radius, nuclear volume (derived), DAPI volume as well as DAPI integrated intensity in control or Auxin treated animals; related to Fig. S3C:

```
# Radius
> res.ftest <- var.test(length ~ zone, data = df_norm)
> res.ftest

        F test to compare two variances

data:  length by zone
F = 0.88747, num df = 79, denom df = 79, p-value = 0.597
alternative hypothesis: true ratio of variances is not equal to 1

> t.test(length~zone, data = df_norm, alternative = "two.sided", var.equal = TRUE)
Two Sample t-test
data:  length by zone
t = 11.886, df = 158, p-value < 2.2e-16
alternative hypothesis: true difference in means is not equal to 0
95 percent confidence interval:
 0.2202022 0.3079699
sample estimates:
mean in group Late Pachytene      mean in group Diplotene
                    1.000000                     0.735914

> tapply(df_norm$length, list(df_norm$zone), function(x) {
+   mean_x <- mean(x)
+   sd_x <- sd(x)
+   n_x <- length(x)
+   paste("Mean:", round(mean_x, 2), "SD:", round(sd_x, 2), "N:", n_x)
+ })
             Late Pachytene                   Diplotene
   "Mean: 1 SD: 0.14 N: 80" "Mean: 0.74 SD: 0.14 N: 80"


# Radius^3 is volume:
> res.ftest <- var.test(length ~ zone, data = df_norm3)
> res.ftest
F test to compare two variances
data:  length by zone
F = 1.2472, num df = 79, denom df = 79, p-value = 0.3282
alternative hypothesis: true ratio of variances is not equal to 1
95 percent confidence interval:
 0.7998712 1.9447723
sample estimates:
ratio of variances
          1.247224
> t.test(length~zone, data = df_norm3, alternative = "two.sided", var.equal = TRUE)
Two Sample t-test
data:  length by zone
t = 10.651, df = 158, p-value < 2.2e-16
alternative hypothesis: true difference in means is not equal to 0
95 percent confidence interval:
 0.4917448 0.7156346
sample estimates:
mean in group Late Pachytene      mean in group Diplotene
                     1.05272                      0.44903

             Late Pachytene                   Diplotene
"Mean: 1.05 SD: 0.38 N: 80" "Mean: 0.45 SD: 0.34 N: 80"


# dapi volume:
> res.ftest <- var.test(volume ~ zone, data = dat_norm)
> res.ftest

        F test to compare two variances

data:  volume by zone
F = 1.3007, num df = 57, denom df = 67, p-value = 0.3
```

```
alternative hypothesis: true ratio of variances is not equal to 1

> t.test(volume~zone, data = dat_norm, alternative = "two.sided", var.equal = TRUE)
Two Sample t-test
data:  volume by zone
t = 12.645, df = 124, p-value < 2.2e-16
alternative hypothesis: true difference in means is not equal to 0
95 percent confidence interval:
 0.3641613 0.4993231
sample estimates:
mean in group Late Pachytene      mean in group Diplotene
                   1.0000000                    0.5682578

> # Use tapply() to report summary statistics by factor levels
> tapply(dat_norm$volume, list(dat_norm$zone), function(x) {
+   mean_x <- mean(x)
+   sd_x <- sd(x)
+   n_x <- length(x)
+   paste("Mean:", round(mean_x, 2), "SD:", round(sd_x, 2), "N:", n_x)
+ })
             Late Pachytene                   Diplotene
    "Mean: 1 SD: 0.2 N: 58" "Mean: 0.57 SD: 0.18 N: 68"


# dapi integrated intensity:
> res.ftest <- var.test(intensity ~ zone, data = dat_norm)
> res.ftest

        F test to compare two variances

data:  intensity by zone
F = 0.39767, num df = 61, denom df = 63, p-value = 0.000393
alternative hypothesis: true ratio of variances is not equal to 1

> t.test(intensity~zone, data = dat_norm, alternative = "two.sided", var.equal = FALSE)
Welch Two Sample t-test
data:  intensity by zone
t = -0.5734, df = 106.76, p-value = 0.5676
alternative hypothesis: true difference in means is not equal to 0
95 percent confidence interval:
 -0.13188821  0.07271006
sample estimates:
mean in group Late Pachytene      mean in group Diplotene
                    1.000000                     1.029589

> # Use tapply() to report summary statistics by factor levels
> tapply(dat_norm$intensity, list(dat_norm$zone), function(x) {
+   mean_x <- mean(x)
+   sd_x <- sd(x)
+   n_x <- length(x)
+   paste("Mean:", round(mean_x, 2), "SD:", round(sd_x, 2), "N:", n_x)
+ })
             Late Pachytene                   Diplotene
   "Mean: 1 SD: 0.22 N: 62" "Mean: 1.03 SD: 0.35 N: 64"
```

[N = 58 (LP), 68 (Dip) for volume; N = 62 (LP), 64 (Dip) for intensity; N = 80 (LP), 80 (Dip) for radius.]

For comparing the proportion of nuclei with at least one RAD-51 focus in each zone across groups, post hoc pair wise comparison of proportions; related to Fig. S4C:

zone 1~5 (sequential sections below); “1” ~ “6” in each section are Control, SPO-11, LMN-1, LMN-1 and SPO-11 (double), LMN-1 and SPO-11 and SUN-1 (triple), and SUN-1:

```
[1] "this is for zone 1"
6-sample test for equality of proportions without continuity correction
data:  c(one_ctrl$count[k], one_spo$count[k], one_lmn$count[k], one_double$count[k],  out of c(tot_ctrl$count[k], tot_spo$count[k], tot_lmn$count[k], tot_double$count[k], one_triple$count[k], one_sun$count[k]) out of     tot_triple$count[k], tot_sun$count[k])
X-squared = 82.788, df = 5, p-value < 2.2e-16
alternative hypothesis: two.sided
sample estimates:
    prop 1     prop 2     prop 3     prop 4     prop 5     prop 6 
0.06081081 0.04545455 0.14640199 0.07843137 0.28901734 0.22775801 
Pairwise comparisons using Pairwise comparison of proportions
data:  c(one_ctrl$count[k], one_spo$count[k], one_lmn$count[k], one_double$count[k],  out of c(tot_ctrl$count[k], tot_spo$count[k], tot_lmn$count[k], tot_double$count[k], one_triple$count[k], one_sun$count[k]) out of     tot_triple$count[k], tot_sun$count[k])
  1       2       3       4       5      
2 0.66674 -       -       -       -      
3 0.01564 0.00021 -       -       -      
4 0.66674 0.21027 0.01741 -       -      
5 1.2e-06 2.0e-10 0.00021 8.2e-08 -      
6 5.6e-05 4.8e-08 0.01446 1.1e-05 0.21027
P value adjustment method: BH

[1] "this is for zone 2"
6-sample test for equality of proportions without continuity correction
data:  c(one_ctrl$count[k], one_spo$count[k], one_lmn$count[k], one_double$count[k],  out of c(tot_ctrl$count[k], tot_spo$count[k], tot_lmn$count[k], tot_double$count[k], one_triple$count[k], one_sun$count[k]) out of     tot_triple$count[k], tot_sun$count[k])
X-squared = 207.72, df = 5, p-value < 2.2e-16
alternative hypothesis: two.sided
sample estimates:
    prop 1     prop 2     prop 3     prop 4     prop 5     prop 6 
0.20851064 0.04891304 0.30373832 0.12745098 0.30000000 0.49450549 
Pairwise comparisons using Pairwise comparison of proportions
data:  c(one_ctrl$count[k], one_spo$count[k], one_lmn$count[k], one_double$count[k],  out of c(tot_ctrl$count[k], tot_spo$count[k], tot_lmn$count[k], tot_double$count[k], one_triple$count[k], one_sun$count[k]) out of     tot_triple$count[k], tot_sun$count[k])
  1       2       3       4       5      
2 6.8e-09 -       -       -       -      
3 0.01345 < 2e-16 -       -       -      
4 0.01817 0.00061 7.9e-08 -       -      
5 0.03296 2.5e-16 0.99158 2.4e-06 -      
6 1.3e-10 < 2e-16 1.1e-06 < 2e-16 2.2e-05
P value adjustment method: BH

[1] "this is for zone 3"
6-sample test for equality of proportions without continuity correction
data:  c(one_ctrl$count[k], one_spo$count[k], one_lmn$count[k], one_double$count[k],  out of c(tot_ctrl$count[k], tot_spo$count[k], tot_lmn$count[k], tot_double$count[k], one_triple$count[k], one_sun$count[k]) out of     tot_triple$count[k], tot_sun$count[k])
X-squared = 910.61, df = 5, p-value < 2.2e-16
alternative hypothesis: two.sided
sample estimates:
    prop 1     prop 2     prop 3     prop 4     prop 5     prop 6 
0.71897810 0.03389831 0.84318182 0.24800000 0.33070866 0.91772152 
Pairwise comparisons using Pairwise comparison of proportions
data:  c(one_ctrl$count[k], one_spo$count[k], one_lmn$count[k], one_double$count[k],  out of c(tot_ctrl$count[k], tot_spo$count[k], tot_lmn$count[k], tot_double$count[k], one_triple$count[k], one_sun$count[k]) out of     tot_triple$count[k], tot_sun$count[k])
  1       2       3       4       5      
2 < 2e-16 -       -       -       -      
3 0.00011 < 2e-16 -       -       -      
4 < 2e-16 6.2e-16 < 2e-16 -       -      
5 < 2e-16 < 2e-16 < 2e-16 0.02978 -      
6 5.7e-10 < 2e-16 0.00353 < 2e-16 < 2e-16
```

```
P value adjustment method: BH

[1] "this is for zone 4"
6-sample test for equality of proportions without continuity correction
data:  c(one_ctrl$count[k], one_spo$count[k], one_lmn$count[k], one_double$count[k],  out
of c(tot_ctrl$count[k], tot_spo$count[k], tot_lmn$count[k], tot_double$count[k],
one_triple$count[k], one_sun$count[k]) out of     tot_triple$count[k], tot_sun$count[k])
X-squared = 827.93, df = 5, p-value < 2.2e-16
alternative hypothesis: two.sided
sample estimates:
    prop 1     prop 2     prop 3     prop 4     prop 5     prop 6
0.56744186 0.06936416 0.89830508 0.21660650 0.36111111 1.00000000
Pairwise comparisons using Pairwise comparison of proportions
data:  c(one_ctrl$count[k], one_spo$count[k], one_lmn$count[k], one_double$count[k],  out
of c(tot_ctrl$count[k], tot_spo$count[k], tot_lmn$count[k], tot_double$count[k],
one_triple$count[k], one_sun$count[k]) out of     tot_triple$count[k], tot_sun$count[k])
  1       2       3       4       5
2 < 2e-16 -       -       -       -
3 < 2e-16 < 2e-16 -       -       -
4 4.1e-15 2.1e-07 < 2e-16 -       -
5 0.00077 8.9e-14 < 2e-16 0.00536 -
6 < 2e-16 < 2e-16 6.8e-07 < 2e-16 < 2e-16
P value adjustment method: BH

[1] "this is for zone 5"
6-sample test for equality of proportions without continuity correction
data:  c(one_ctrl$count[k], one_spo$count[k], one_lmn$count[k], one_double$count[k],  out
of c(tot_ctrl$count[k], tot_spo$count[k], tot_lmn$count[k], tot_double$count[k],
one_triple$count[k], one_sun$count[k]) out of     tot_triple$count[k], tot_sun$count[k])
X-squared = 327.26, df = 5, p-value < 2.2e-16
alternative hypothesis: two.sided
sample estimates:
    prop 1     prop 2     prop 3     prop 4     prop 5     prop 6
0.17391304 0.04705882 0.66666667 0.11206897 0.30379747 0.98809524
Pairwise comparisons using Pairwise comparison of proportions
data:  c(one_ctrl$count[k], one_spo$count[k], one_lmn$count[k], one_double$count[k],  out
of c(tot_ctrl$count[k], tot_spo$count[k], tot_lmn$count[k], tot_double$count[k],
one_triple$count[k], one_sun$count[k]) out of     tot_triple$count[k], tot_sun$count[k])
  1       2       3       4       5
2 0.0019  -       -       -       -
3 3.7e-13 < 2e-16 -       -       -
4 0.2813  0.0738  < 2e-16 -       -
5 0.0738  9.4e-08 4.2e-07 0.0019  -
6 < 2e-16 < 2e-16 5.0e-08 < 2e-16 < 2e-16
P value adjustment method: BH


# sample sizes: zone 1~5 (sequential sections below); the six numbers in each section are
(from left to right) Control, SPO-11, LMN-1, LMN-1 and SPO-11 (double), LMN-1 and SPO-11
and SUN-1 (triple), and SUN-1:

[1] "this is for zone 1"
[1] "# of nuclei with at least one RAD-51 focus:"
[1]  9 11 59 20 50 64
[1] "# of total nuclei:"
[1] 148 242 403 255 173 281

[1] "this is for zone 2"
[1] "# of nuclei with at least one RAD-51 focus:"
[1]  49  18 130  39  69 135
[1] "# of total nuclei:"
[1] 235 368 428 306 230 273

[1] "this is for zone 3"
[1] "# of nuclei with at least one RAD-51 focus:"
[1] 197  12 371  93  84 290
[1] "# of total nuclei:"
[1] 274 354 440 375 254 316
```

```
[1] "this is for zone 4"
[1] "# of nuclei with at least one RAD-51 focus:"
[1] 122  24 318  60  39 247
[1] "# of total nuclei:"
[1] 215 346 354 277 108 247

[1] "this is for zone 5"
[1] "# of nuclei with at least one RAD-51 focus:"
[1]  16   8 104  13  24  83
[1] "# of total nuclei:"
[1]  92 170 156 116  79  84
```

For comparing the percentage of X-chromosome pairing or complete synapsis; related to Fig. S6C:

```
#Pairing:
> res1
2-sample test for equality of proportions with continuity correction
data:  c(9, 30) out of c(201, 175)
X-squared = 14.808, df = 1, p-value = 0.000119
alternative hypothesis: two.sided
95 percent confidence interval:
 -0.1947299 -0.0585750
sample estimates:
    prop 1     prop 2
0.04477612 0.17142857
> res2
2-sample test for equality of proportions with continuity correction
data:  c(150, 224) out of c(274, 248)
X-squared = 79.374, df = 1, p-value < 2.2e-16
alternative hypothesis: two.sided
95 percent confidence interval:
 -0.4291007 -0.2824604
sample estimates:
   prop 1    prop 2
0.5474453 0.9032258
> res3
2-sample test for equality of proportions with continuity correction
data:  c(297, 269) out of c(307, 273)
X-squared = 1.2829, df = 1, p-value = 0.2574
alternative hypothesis: two.sided
95 percent confidence interval:
 -0.045824466  0.009981916
sample estimates:
   prop 1    prop 2
0.9674267 0.9853480
> res4
2-sample test for equality of proportions with continuity correction
data:  c(258, 172) out of c(263, 172)
X-squared = 1.8463, df = 1, p-value = 0.1742
alternative hypothesis: two.sided
95 percent confidence interval:
 -0.040324288  0.002301475
sample estimates:
   prop 1    prop 2
0.9809886 1.0000000
> res5
2-sample test for equality of proportions with continuity correction
data:  c(132, 59) out of c(135, 63)
X-squared = 1.1058, df = 1, p-value = 0.293
alternative hypothesis: two.sided
95 percent confidence interval:
 -0.03551592  0.11805560
sample estimates:
   prop 1    prop 2
0.9777778 0.9365079
> res1$p.value
[1] 0.000119012
> res2$p.value
[1] 5.141022e-19
> res3$p.value
[1] 0.2573633
> res4$p.value
[1] 0.1742091
> res5$p.value
[1] 0.2930057



## synapsis:
2-sample test for equality of proportions with continuity correction
```

```
data:  c(0, 5) out of c(209, 192)
X-squared = 3.5994, df = 1, p-value = 0.0578
alternative hypothesis: two.sided
95 percent confidence interval:
 -0.053565110  0.001481777
sample estimates:
    prop 1     prop 2 
0.00000000 0.02604167 
> res7
2-sample test for equality of proportions with continuity correction
data:  c(52, 131) out of c(280, 238)
X-squared = 73.308, df = 1, p-value < 2.2e-16
alternative hypothesis: two.sided
95 percent confidence interval:
 -0.4464952 -0.2829166
sample estimates:
   prop 1    prop 2 
0.1857143 0.5504202 
> res8
2-sample test for equality of proportions with continuity correction
data:  c(254, 259) out of c(307, 286)
X-squared = 7.1089, df = 1, p-value = 0.00767
alternative hypothesis: two.sided
95 percent confidence interval:
 -0.13579083 -0.02067485
sample estimates:
   prop 1    prop 2 
0.8273616 0.9055944 
> res9
2-sample test for equality of proportions with continuity correction
data:  c(248, 153) out of c(261, 178)
X-squared = 9.8798, df = 1, p-value = 0.001671
alternative hypothesis: two.sided
95 percent confidence interval:
 0.02845383 0.15282819
sample estimates:
   prop 1    prop 2 
0.9501916 0.8595506 
> res10
2-sample test for equality of proportions with continuity correction
data:  c(61, 26) out of c(135, 64)
X-squared = 0.20501, df = 1, p-value = 0.6507
alternative hypothesis: two.sided
95 percent confidence interval:
 -0.1126318  0.2038355
sample estimates:
   prop 1    prop 2 
0.4518519 0.4062500 
> res6$p.value
[1] 0.05780018
> res7$p.value
[1] 1.109453e-17
> res8$p.value
[1] 0.007670096
> res9$p.value
[1] 0.001671028
> res10$p.value
[1] 0.6507078
```

Two-way ANOVA on the mean and relative standard deviation of ZYG-12::GFP intensity, related to Fig. S7A,B:

```
> # Use tapply() to report summary statistics by factor levels
> # for mean
> tapply(df_norm$mean, list(df_norm$zone, df_norm$group), function(x) {
+   mean_x <- mean(x)
+   sd_x <- sd(x)
+   n_x <- length(x)
+   paste("Mean:", round(mean_x, 2), "SD:", round(sd_x, 2), "N:", n_x)
+ })
    - Aux                            + Aux
TZ  "Mean: 1 SD: 0.19 N: 30"    "Mean: 1 SD: 0.21 N: 30"
MP  "Mean: 1.31 SD: 0.17 N: 30" "Mean: 1.15 SD: 0.23 N: 30"
LP  "Mean: 1.29 SD: 0.31 N: 30" "Mean: 0.79 SD: 0.3 N: 30"
Dip "Mean: 1.14 SD: 0.35 N: 30" "Mean: 0.77 SD: 0.34 N: 30"

> # for relative standard deviation
> tapply(df_all$coef_v, list(df_all$zone, df_all$group), function(x) {
+   mean_x <- mean(x)
+   sd_x <- sd(x)
+   n_x <- length(x)
+   paste("Mean:", round(mean_x, 2), "SD:", round(sd_x, 2), "N:", n_x)
+ })
    - Aux                            + Aux
TZ  "Mean: 0.42 SD: 0.1 N: 30"  "Mean: 0.41 SD: 0.13 N: 30"
MP  "Mean: 0.15 SD: 0.05 N: 30" "Mean: 0.21 SD: 0.05 N: 30"
LP  "Mean: 0.18 SD: 0.05 N: 30" "Mean: 0.21 SD: 0.07 N: 30"
Dip "Mean: 0.16 SD: 0.04 N: 30" "Mean: 0.25 SD: 0.09 N: 30"

> res.aov3 <- aov(mean ~ zone * group, data = df_norm)
> summary(res.aov3)
             Df Sum Sq Mean Sq F value   Pr(>F)
zone          3  2.647   0.882  12.073 2.26e-07 ***
group         1  3.985   3.985  54.530 2.73e-12 ***
zone:group    3  2.183   0.728   9.954 3.38e-06 ***
Residuals   232 16.956   0.073
---
Signif. codes:  0 ‘***’ 0.001 ‘**’ 0.01 ‘*’ 0.05 ‘.’ 0.1 ‘ ’ 1
> res.aov4 <- aov(coef_v ~ zone * group, data = df_norm)
> summary(res.aov4)
             Df Sum Sq Mean Sq F value   Pr(>F)
zone          3 2.2901  0.7634 126.651  < 2e-16 ***
group         1 0.1279  0.1279  21.212 6.78e-06 ***
zone:group    3 0.0779  0.0260   4.306  0.00559 **
Residuals   232 1.3983  0.0060
---
Signif. codes:  0 ‘***’ 0.001 ‘**’ 0.01 ‘*’ 0.05 ‘.’ 0.1 ‘ ’ 1
```

one-way ANOVA and post hoc pairwise t-tests for comparing mean intensity of V5 staining per nucleus; related to Fig. S12B

```
# CeCL6, CeCL31, CeCL39, CeCL41 are lmn-1::AID::V5, lmn-1::AID::V5 and sun-1::AID::V5, lmn-1::AID::V5 and HA::AID::zyg-12, lmn-1::AID::V5 and sun-1::AID::V5 and spo-11::AID::3xFlag, respectively
```

```
> tapply(df$X1, df$X2, mean)
 CeCL6_Ctrl   CeCL6_Aux CeCL31_Ctrl  CeCL31_Aux CeCL39_Ctrl  CeCL39_Aux CeCL41_Ctrl  CeCL41_Aux 
  40783.370   -1389.921   48642.771    1132.837   56927.873   -1337.068   42032.135   -1333.961 
> tapply(df$X1, df$X2, sd)
 CeCL6_Ctrl   CeCL6_Aux CeCL31_Ctrl  CeCL31_Aux CeCL39_Ctrl  CeCL39_Aux CeCL41_Ctrl  CeCL41_Aux 
  12133.419    3255.423   17092.548    2205.869   28170.082    5342.293   17125.763    5929.677 
> tapply(df$X1, df$X2, length)
 CeCL6_Ctrl   CeCL6_Aux CeCL31_Ctrl  CeCL31_Aux CeCL39_Ctrl  CeCL39_Aux CeCL41_Ctrl  CeCL41_Aux 
         30          30          30          30          30          30          30          30 
> 
> res.aov <- aov(df$X1 ~ df$X2)
> summary(res.aov)
             Df    Sum Sq   Mean Sq F value Pr(>F)    
df$X2         7 1.423e+11 2.033e+10   101.3 <2e-16 ***
Residuals   232 4.656e+10 2.007e+08                   
---
Signif. codes:  0 '***' 0.001 '**' 0.01 '*' 0.05 '.' 0.1 ' ' 1
> pairwise.t.test(df$X1, df$X2,
+                 p.adjust.method = "BH")

	Pairwise comparisons using t tests with pooled SD 

data:  df$X1 and df$X2 

            CeCL6_Ctrl CeCL6_Aux CeCL31_Ctrl CeCL31_Aux CeCL39_Ctrl CeCL39_Aux CeCL41_Ctrl
CeCL6_Aux   < 2e-16    -         -           -          -           -          -          
CeCL31_Ctrl 0.046      < 2e-16   -           -          -           -          -          
CeCL31_Aux  < 2e-16    0.584     < 2e-16     -          -           -          -          
CeCL39_Ctrl 2.6e-05    < 2e-16   0.036       < 2e-16    -           -          -          
CeCL39_Aux  < 2e-16    0.999     < 2e-16     0.584      < 2e-16     -          -          
CeCL41_Ctrl 0.821      < 2e-16   0.096       < 2e-16    9.9e-05     < 2e-16    -          
CeCL41_Aux  < 2e-16    0.999     < 2e-16     0.584      < 2e-16     0.999      < 2e-16    

P value adjustment method: BH 
```

Pairwise Mann-Whitney test for comparing germline apoptosis using CED-1::GFP; related to Fig. S13A

ctrl, syp-1, syp-1-het, zyg-12-AID, zyg-12-ctrl are: *wild type*, *syp-1(me17)*, *syp-1(me17)/nT1*, *AID::zyg-12* +Auxin, *AID::zyg-12* -Auxin, respectively

```
> tapply(dat$apoptosis, dat$group, mean)
       ctrl       syp-1   syp-1-het  zyg-12-AID zyg-12-ctrl 
   11.90909    43.00000    18.33333    27.00000    16.00000 
> tapply(dat$apoptosis, dat$group, sd)
       ctrl       syp-1   syp-1-het  zyg-12-AID zyg-12-ctrl 
   3.986340   13.883443    2.875181    7.571878    7.905694 
> tapply(dat$apoptosis, dat$group, length)
       ctrl       syp-1   syp-1-het  zyg-12-AID zyg-12-ctrl 
         11           9           6          10          13 



        Pairwise comparisons using Wilcoxon rank sum test 

data:  apoptosis and group 

            ctrl   syp-1  syp-1-het zyg-12-AID
syp-1       0.0011 -      -         -         
syp-1-het   0.0092 0.0065 -         -         
zyg-12-AID  0.0011 0.0161 0.0412    -         
zyg-12-ctrl 0.3225 0.0017 0.3225    0.0113    

P value adjustment method: BH
```

group	apoptosis
ctrl	15
ctrl	13
ctrl	8
ctrl	16
ctrl	10
ctrl	10
ctrl	14
ctrl	18
ctrl	10
ctrl	13
ctrl	4
syp-1-het	17
syp-1-het	16
syp-1-het	18
syp-1-het	17
syp-1-het	18
syp-1-het	24

syp-1	26
syp-1	56
syp-1	40
syp-1	53
syp-1	20
syp-1	62
syp-1	37
syp-1	50
syp-1	43
zyg-12-ctrl	10
zyg-12-ctrl	5
zyg-12-ctrl	22
zyg-12-ctrl	27
zyg-12-ctrl	17
zyg-12-ctrl	15
zyg-12-ctrl	9
zyg-12-ctrl	9

zyg-12-ctrl	13
zyg-12-ctrl	9
zyg-12-ctrl	25
zyg-12-ctrl	17
zyg-12-ctrl	30
zyg-12-AID	17
zyg-12-AID	23
zyg-12-AID	26
zyg-12-AID	25
zyg-12-AID	22
zyg-12-AID	35
zyg-12-AID	17
zyg-12-AID	33
zyg-12-AID	38
zyg-12-AID	34

Mann-Whitney test for compairing germline apoptosis using acridine orange; related to Fig. S13B

```
> tapply(dat$count, dat$group, mean)
SUN-1 Ctrl 12hr  SUN-1 AID 12hr
       5.947368        6.333333
> tapply(dat$count, dat$group, sd)
SUN-1 Ctrl 12hr  SUN-1 AID 12hr
       2.778363        3.086975
> tapply(dat$count, dat$group, length)
SUN-1 Ctrl 12hr  SUN-1 AID 12hr
             19              18


> wilcox.test(count~group, data = dat, exact = FALSE, alternative = "two.sided")

        Wilcoxon rank sum test with continuity correction

data:  count by group
W = 161, p-value = 0.7705
alternative hypothesis: true location shift is not equal to 0
```

count	group
6	SUN-1 AID 12hr
15	SUN-1 AID 12hr
7	SUN-1 AID 12hr
7	SUN-1 AID 12hr
5	SUN-1 AID 12hr
9	SUN-1 AID 12hr
8	SUN-1 AID 12hr
9	SUN-1 AID 12hr
9	SUN-1 AID 12hr
3	SUN-1 AID 12hr
3	SUN-1 AID 12hr
7	SUN-1 AID 12hr
3	SUN-1 AID 12hr
4	SUN-1 AID 12hr
6	SUN-1 AID 12hr
2	SUN-1 AID 12hr
5	SUN-1 AID 12hr
6	SUN-1 AID 12hr
7	SUN-1 Ctrl 12hr
7	SUN-1 Ctrl 12hr
6	SUN-1 Ctrl 12hr
3	SUN-1 Ctrl 12hr
6	SUN-1 Ctrl 12hr
7	SUN-1 Ctrl 12hr
13	SUN-1 Ctrl 12hr
6	SUN-1 Ctrl 12hr
10	SUN-1 Ctrl 12hr
7	SUN-1 Ctrl 12hr
4	SUN-1 Ctrl 12hr
8	SUN-1 Ctrl 12hr
1	SUN-1 Ctrl 12hr
6	SUN-1 Ctrl 12hr
5	SUN-1 Ctrl 12hr
2	SUN-1 Ctrl 12hr
3	SUN-1 Ctrl 12hr
5	SUN-1 Ctrl 12hr
7	SUN-1 Ctrl 12hr

Statistics for comparing the extent of asymmetry, L4440/*samp-1(RNAi)* in EP, linear regression/ANOVA, "Y" being normalized intensity, "X" being angles from 1-180° (combined from 0-360°), "1" being L4440, "2" being samp-1 RNAi; related to Fig. S17B:

Sample size: # of nuclei
L4440, n = 32; *samp-1(RNAi)*, n = 32;

```
> fit <- lm(Y ~ X * id,data=df_fit)
> anova(fit)
Analysis of Variance Table
Response: Y
            Df  Sum Sq Mean Sq F value    Pr(>F)
X            1  46.980  46.980  936.15 < 2.2e-16 ***
id           1  11.414  11.414  227.45 < 2.2e-16 ***
X:id         1   9.651   9.651  192.31 < 2.2e-16 ***
Residuals 6280 315.161   0.050
---
Signif. codes:  0 '***' 0.001 '**' 0.01 '*' 0.05 '.' 0.1 ' ' 1
> print(fit)
Call:
lm(formula = Y ~ X * id, data = df_fit)
Coefficients:
(Intercept)            X           id         X:id
  1.1572230    0.0005914    0.2182465   -0.0014932
```

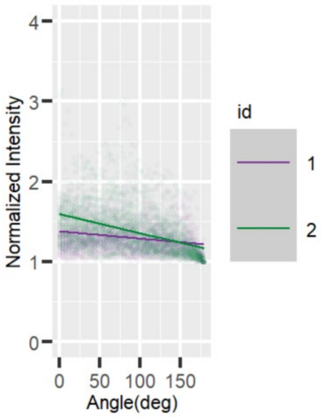

Statistics for comparing the extent of asymmetry, L4440/*lem-2(RNAi)* in EP, linear regression/ANOVA, "Y" being normalized intensity, "X" being angles from 1-180° (combined from 0-360°), "1" being L4440, "2" being lem-2 RNAi; related to Fig. S17D:

Sample size: # of nuclei
L4440, n = 51; *lem-2(RNAi)*, n = 35;

```
Analysis of Variance Table
Response: Y
            Df Sum Sq Mean Sq F value    Pr(>F)
X            1  67.01  67.006 1801.59 < 2.2e-16 ***
id           1  27.54  27.538  740.43 < 2.2e-16 ***
X:id         1  20.63  20.632  554.72 < 2.2e-16 ***
Residuals 9055 336.78   0.037
---
Signif. codes:  0 '***' 0.001 '**' 0.01 '*' 0.05 '.' 0.1 ' ' 1
> print(fit)
Call:
lm(formula = Y ~ X * id, data = df_fit)
Coefficients:
(Intercept)            X           id         X:id
  1.0606633    0.0009627    0.2777642   -0.0018549

OR:
> res.aov3 <- aov(Y ~ X * id, data = df_fit)
> summary(res.aov3)
              Df Sum Sq Mean Sq F value Pr(>F)
X              1   67.0   67.01  1801.6 <2e-16 ***
id             1   27.5   27.54   740.4 <2e-16 ***
X:id           1   20.6   20.63   554.7 <2e-16 ***
Residuals   9055  336.8    0.04
---
Signif. codes:  0 '***' 0.001 '**' 0.01 '*' 0.05 '.' 0.1 ' ' 1
```

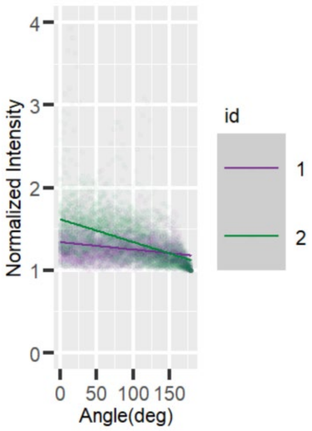

Statistics for comparing nuclear size; Related to Fig. S18B:

```
# “1, 2, 3” are Control RNAi -Auxin, Control RNAi +Auxin, and samp-1(RNAi) + Auxin

> tapply(dat$diameter, dat$group, mean)
       1        2        3 
3.150633 3.203191 3.283186 
> tapply(dat$diameter, dat$group, sd)
        1         2         3 
0.3222013 0.5183622 0.6213325 
> tapply(dat$diameter, dat$group, length)
  1   2   3 
 79  94 113 


> #perform Welch's ANOVA
> oneway.test(diameter ~ group, data = dat, var.equal = FALSE)

	One-way analysis of means (not assuming equal variances)

data:  diameter and group
F = 1.8829, num df = 2.00, denom df = 185.96, p-value = 0.155


> games_howell_test(dat, diameter ~ group)
# A tibble: 3 x 8
  .y.      group1 group2 estimate conf.low conf.high p.adj p.adj.signif
* <chr>    <chr>  <chr>     <dbl>    <dbl>     <dbl> <dbl> <chr>       
1 diameter 1      2        0.0526  -0.100      0.205 0.695 ns          
2 diameter 1      3        0.133   -0.0300     0.295 0.134 ns          
3 diameter 2      3        0.0800  -0.107      0.267 0.571 ns          
```
